# *MECP2* mutations rewire human ESC fate and bias cortical lineage commitment

**DOI:** 10.1016/j.stemcr.2026.102895

**Published:** 2026-04-23

**Authors:** Marion Guillon, Margaux Brin, Elodie Gabet, Justine Gromaire, Mathéa Bernard, Laetitia Laurent, Théo Rabin, Lisa Bianchin, Marie Veziano, Julie Kloda, Alexia Bernard, Laila Asali, Yi Liu, Anthony Flamier

**Affiliations:** 1 Centre de recherche Azrieli du CHU Sainte-Justine, Montreal, QC, Canada; 2 Regenerative Medicine Program, Ottawa Hospital Research Institute, Ottawa, ON, Canada; 3 Department of Cellular and Molecular Medicine, Faculty of Medicine, University of Ottawa, Ottawa, ON, Canada; 4 Department of Neurosciences, Université de Montréal, Montreal, QC, Canada

**Keywords:** MECP2, Rett syndrome, pluripotency, EMX1, transcriptomics, neurodevelopment, cerebral organoids, lineage specification, stem cell differentiation

## Abstract

Rett syndrome arises from loss-of-function mutations in the X-linked chromatin regulator MECP2, yet the earliest molecular derailments in development are poorly defined. Using isogenic human embryonic stem cell (hESC) models carrying three patient-derived *MECP2* mutations, we followed the transcriptome from pluripotency through neuroectoderm, neural stem/progenitor stages. Developmental stage dominated transcriptional variance, but mutants shared a secondary program enriched for synaptic-membrane and extracellular matrix genes. Single-cell/bulk profiling at the embryonic stem cell (ESC) stage revealed partial naïve-like drift, marked by the up-regulation of the naïve-enriched factor *ZFP42*/REX1 and related markers in *MECP2*-mutant lines. Among convergently dysregulated genes, the cortical determinant *EMX1* showed an abnormal developmental trajectory, early repression followed by overshoot, and was consistently altered across independent Rett PSC models. Single-nucleus RNA-seq of cerebral organoids uncovered allele-specific yet convergent disturbances in cortical lineage allocation. These data chart a continuous developmental trajectory for *MECP2*-mutant cells and nominate naïve-like drift and mis-timed *EMX1* expression as tractable entry points for dissecting Rett pathogenesis.

## Highlights

• MECP2 mutations induce an early naïve-like transcriptional drift in hESCs
• EMX1 shows a conserved abnormal developmental trajectory across Rett models
• Shared transcriptional programs emerge during neural induction in MECP2 mutants
• Rett cerebral organoids display mutation-specific shifts in lineage allocation

## Graphical abstract

**Figure:**
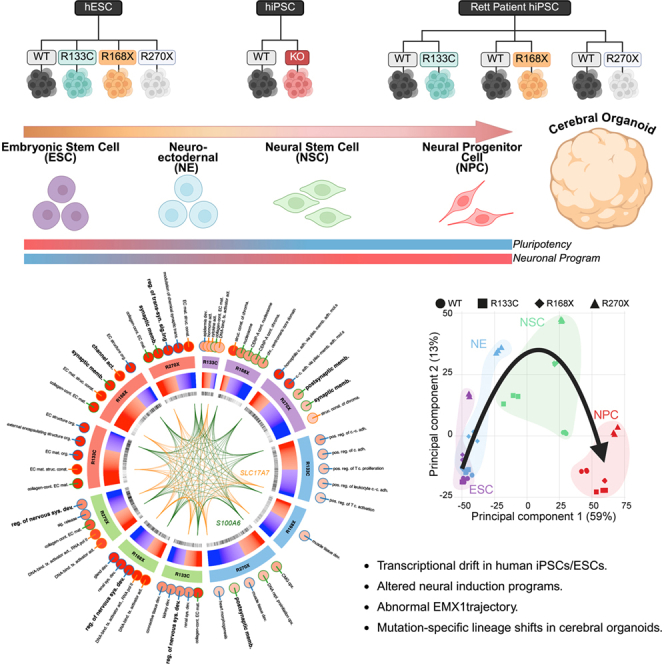

## Teaser

In this article, Flamier and colleagues show that *MECP2* mutations perturb human neurodevelopment from the pluripotent stage onward. Using isogenic hESC and organoid models, they identify an early naïve-like transcriptional drift, abnormal EMX1 timing, and convergent defects in cortical lineage allocation, revealing continuous developmental vulnerability in Rett syndrome.

## Introduction

Rett syndrome (RTT) is a severe X-linked neurodevelopmental disorder that affects ≈1 in 10,000 girls worldwide and, in rare surviving males, presents with an even more severe encephalopathy (Liu et al., 2025b; Petriti et al., 2023). After a seemingly normal first 6–18 months, infants enter a protracted period of neuro-regression marked by loss of purposeful hand use and speech, seizures, gait ataxia, autistic features, and severe intellectual disability (Guy et al., 2001; Liao, 2019; Liu et al., 2025a, 2025b; Moog et al., 2003; Neul and Zoghbi, 2004; Petriti et al., 2023; Raspa et al., 2025). Structural MRI and postmortem studies consistently show reduced total brain volume, cortical thinning, simplified dendritic arbors, and a shift in excitatory-inhibitory balance, suggesting that pathogenic processes likely begin during early corticogenesis and extend into postnatal synaptic maturation (Armstrong et al., 1995; Chao et al., 2007; Dani et al., 2005; Ip et al., 2018; Reiss et al., 1993).

More than 95% of classic RTT cases arise from *de novo* loss-of-function mutations in *MECP2*, which encodes methyl-CpG-binding protein 2 (MECP2). (Amir et al., 1999) Historically, MECP2 was viewed as a canonical methyl-DNA reader that blankets the neuronal genome and recruits co-repressor complexes to silence transcription (Nan et al., 1998). Recent molecular studies on adult mouse cortex and human embryonic stem cell (ESC)-derived neurons refine this view: MECP2 binds preferentially to discrete enhancer-like elements, termed MECP2-binding hotspots (MBHs), and can do so even in regions with relatively low CpG methylation (Bajikar et al., 2025; Y. Li et al., 2013; Liu et al., 2024; Liu et al., 2025b; Mishra et al., 2025). Clusters of MBHs cooperate to the dampen transcription of genes enriched for neuronal functions, revealing an intragenic, partly methylation-independent mode of repression that likely complements the classical methylation-dependent mechanism (Liu et al., 2024; Mishra et al., 2025). We and others also found that MECP2 binds both unmethylated and methylated cytosines to act as both a transcriptional repressor (through its NCoR domain) and activator (through RNA polymerase II recruitment) in neurons (Chahrour et al., 2008; Li et al., 2013; Liu et al., 2024; Lyst et al., 2013; Sharifi et al., 2024). However, the mechanistic context of these findings remains unclear in early development. Mammalian ESCs and blastocyst-stage embryos are globally hypomethylated, a state long thought to preclude meaningful MECP2 engagement and thus to spare pluripotent cells from RTT-associated lesions (Guo et al., 2014). The discovery that MECP2 can also bind hypomethylated regions to regulate transcription now raises the possibility that MECP2 deficiency could perturb transcriptional programs as early as the hypomethylated ESC stage, well before neurons are specified (Liu et al., 2024, 2025b). Determining whether such early dysregulation contributes to later cortical deficits is therefore a central unanswered question that we address in this study.

To interrogate this possibility in a continuous human model, we used isogenic human male hESC lines harboring three recurrent RTT mutations (R133C, R168X, and R270X) (Neul et al., 2008) and performed longitudinal profiling across four matched developmental milestones—pluripotent ESC, neuro-ectoderm (NE), neural stem cell (NSC) and neural progenitor cell (NPC)—and in long-term cerebral organoids. We further used RTT patient-derived induced pluripotent stem cell (iPSC) lines to validate our observations. By integrating bulk and single-cell RNA sequencing with gene-set enrichment and machine-learning classifiers, we uncovered a stage-persistent, MECP2-dependent transcriptional program that elevates naïve-enriched stem cell markers and increases ESC proliferation without full conversion to naive pluripotency, enhances synaptic-membrane pathways during neural induction, and imposes an abnormal developmental trajectory of the cortical radial-glia determinant EMX1. In three-month cerebral organoids, these early changes are accompanied by allele-specific but convergent alterations in cortical lineage allocation, with shifted proportions of glutamatergic neuron lineage, inhibitory neurons, and glial populations. These data reveal discrete molecular lesions that precede overt neuronal dysfunction and provide new developmental entry points for mechanistic dissection in RTT.

## Results

### Stage-resolved transcriptomics reveals early, genotype-specific disruptions in MECP2-mutant differentiation

**Figure 1:**
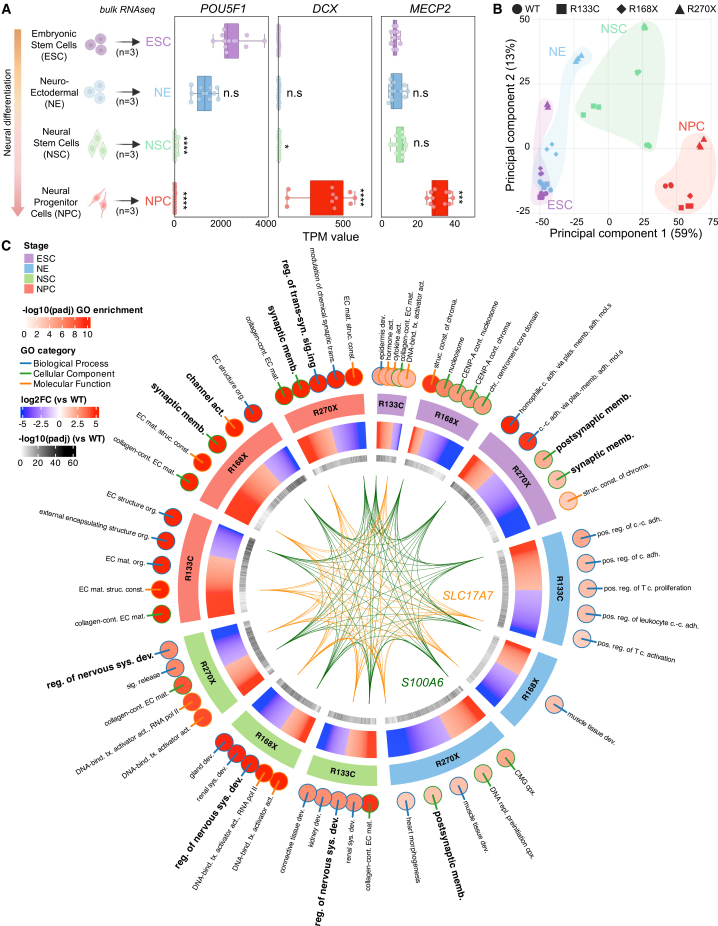
*Stage-resolved transcriptomics reveal early gene-network disruption in MECP2-mutant hESC differentiation* (A) Expression dynamics of key developmental markers across the four matched stages analyzed on 4 RTT hES cell lines (WT, R133C, R168X et R270X). Box-and-scatter plots show transcripts per million (TPM; *n* = 3 biological replicates per stage and genotype) for the pluripotency factor *POU5F1*/OCT4, the early neuronal marker *DCX*, and *MECP2* itself. Tukey-adjusted post-hoc significance relative to the ESC stage (^∗^*p* < 0.05, ^∗∗∗^*p* < 0.001, and ^∗∗∗∗^*p* < 0.0001). (B) Principal component analysis of the complete bulk RNA-seq dataset. (C) Circular multi-layer GO enrichment map integrating all three mutants. Outer colored dots show the top 5 GO terms significantly enriched at each stage (ESC, NE, NSC, NPC) and for each genotype versus WT (neural GO terms in bold); color scale encodes log2 fold-change versus WT (red, up-regulated; blue, down-regulated). Inner ring indicates –log10 padj of differential gene expression. The center chord diagram links the two transcripts (*S100A6*, green chords; *SLC17A7*, orange chords) that are consistently dysregulated in every genotype at every stage.

To test whether MECP2 loss perturbs human neurodevelopment from its earliest hypomethylated state, we profiled three previously described CRISPR-edited, isogenic hESC lines carrying common RTT mutations (R133C, R168X, and R270X) alongside WT controls as they were sequentially coaxed through ESC (day 0), NE (day 3), NSC (day 7) and NPC (day 21) stages (Figure 1A).(Liu et al., 2024) As expected, the pluripotency factors plunged after the ESC stage, whereas neuronal markers became detectable at NSC and rose thereafter, confirming efficient lineage induction (Figures 1A and S1A–S1C). *MECP2* itself remained low but readily measurable at ESCs (5–10 TPM), corroborating published observations (Figure 1A) (Li et al., 2013). *MECP2* increased modestly at NE/NSC and surged at NPC (Figure 1A). This suggests that residual embryonic expression could already influence transcriptional programs. Principal-component analysis showed that PC1 (59% variance) stratified samples by developmental stage, validating the differentiation axis, whereas PC2 (13%) captured mutation-specific variance that emerged progressively: R270X diverged from WT at the ESC stage, R168X detached by NE, and all mutants formed distinct genotype clusters by NSC/NPC (Figure 1B). GO enrichment revealed significant dysregulation beginning at ESCs, most strikingly in R270X, where synaptic-membrane genes were already mis-expressed (Figure 1C). NSC samples displayed enrichment for the “regulation of nervous-system development,” and NPCs showed broad perturbation of synaptic and ion-channel terms (Figure 1C). We observed common gene dysregulations across genotypes and stages (Figures 1C and S1D). Two transcripts were consistently altered in every genotype at every stage: *S100A6*, a calcium-binding protein implicated in cytoskeletal dynamics and stem-cell proliferation, and *SLC17A7*/*VGLUT1*, the principal vesicular glutamate transporter essential for excitatory neurotransmission (Figure 1C) (Cheret et al., 2021; Donato et al., 2017; Wang et al., 2023). Early dysregulation of *S100A6* suggests cytoskeletal or proliferative defects could precede neurogenesis, whereas persistent mis-expression of *SLC17A7* foreshadows later synaptic dysfunction (Cheret et al., 2021; Jurewicz et al., 2020; Li et al., 2017). Collectively, these findings suggest that *MECP2* mutations impose transcriptomic disturbances as early as the blastocyst-like ESC stage, raising the possibility that altered lineage trajectories, not only neuronal maturation defects, contribute to RTT pathogenesis.

### Machine-learning models accurately decode developmental stage and highlight early neuronal mis-timing in MECP2 mutants

**Figure 2:**
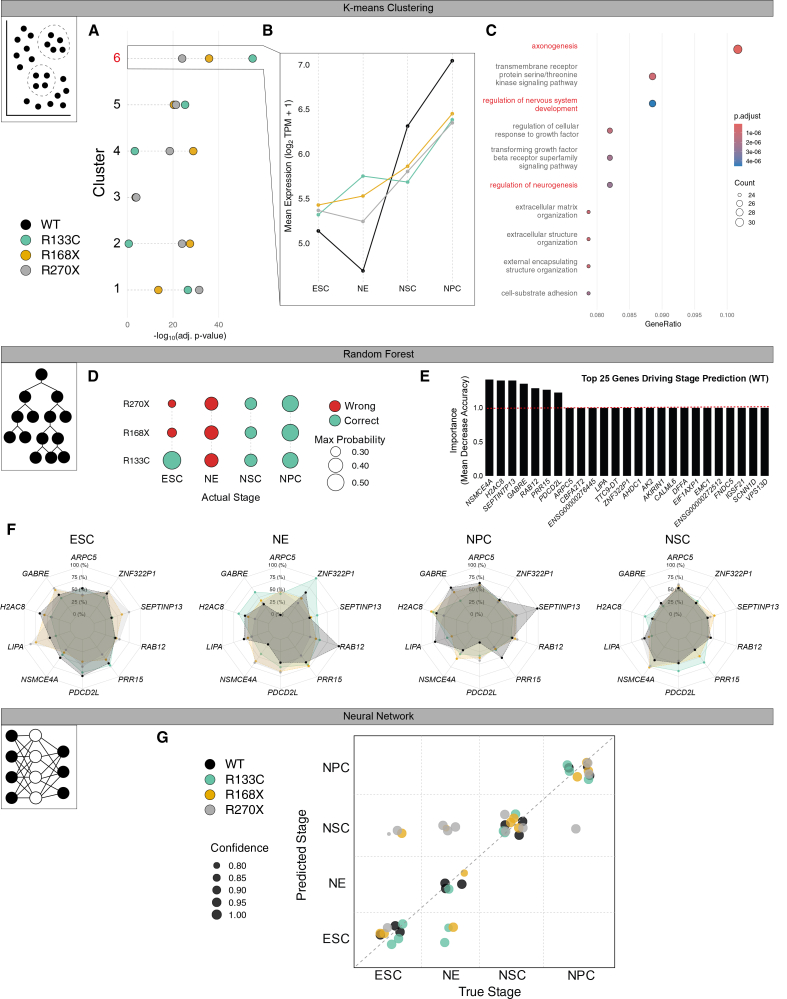
Machine-learning classifiers expose premature neuronal program activation in MECP2 mutants (A) Adjusted *p*-values (–log_10_ scale) for the six gene clusters identified by K-means (left margin indicates cluster number). (B) Mean expression profile (log_2_ TPM +1) of the genes represented in cluster 6 across the four sampled stages and the four genotypes. Genotypes are color-coded as in the inset legend. (C) Dot plot of GO terms enriched in Cluster 6. Dot diameter corresponds to the number of genes annotated to the term; color encodes Benjamini-Hochberg adjusted *p*-value(D) Confusion matrix displays random-forest stage predictions for each genotype. Circle size denotes the classifier’s maximum posterior probability; color indicates correct or incorrect assignment. (E) Bar chart of the top 25 genes ranked by mean decrease in accuracy when permuted, obtained from the model trained on WT samples. (F) Radar plots of the ten most informative transcripts (E) show their median expression at each stage for WT and the three *MECP2*-mutant lines. (G) Scatterplot compares predicted versus true developmental stages for all samples. Point color denotes genotype; point size reflects the model’s confidence (posterior probability). The dashed diagonal marks perfect agreement.

We asked whether *MECP2* mutations create transcriptomic shifts large enough for detection by machine-learning and, if so, which genes drive those shifts. To this end, we analyzed our bulk RNA sequencing matrix with three complementary algorithms: Unsupervised k-means clustering served for pattern discovery; a supervised random-forest ensemble offered interpretable prediction; a feedforward neural network provided high-capacity validation. K-means partitioned genes into six clusters (Figures 2A, S2A, and S2B). Cluster 6 showed a steady increase in expression from the ESC/NE stage to the NSC and NPC stages (Figures 2B and S2B). Notably, this trajectory was the most significantly altered in the three mutant lines (Figures 2A and 2B). Gene-Ontology analysis linked this cluster to axonogenesis (e.g., *CACNA1F*, *CNTN2*, and *DLX5*) and regulation of nervous-system development, implying that neuronal programs are engaged unusually early in *MECP2*-deficient pluripotent cells (Figure 2C). The random forest, trained on labeled samples, achieved more than 95% cross-validated accuracy (Figure 2D). Feature-importance scores highlighted ten transcripts that dominate stage discrimination (Figure 2E). For example, *GABRE,* encoding the ε-subunit of the GABA-A receptor shaping inhibitory tone, was elevated in mutants at ESC and NE but normalized or fell below wild-type at NSC and NPC (Figure 2F) (Nguyen and Nicoll, 2018; Sigel and Steinmann, 2012). *PDCD2L*, with a similar profile, plays a role in neurodevelopment, particularly in programmed cell death (PCD) and ribosomal biogenesis (Figure 2F). (Li et al., 2023a) PCD is essential for stem cell maintenance and neurodevelopment, as it involves the elimination of a subset of neurons to refine neural circuits (Guo et al., 2024). Finally, *NSMCE4A*, the most important gene of this classifier, is part of the SMC5/6 complex, which is key for chromosome architecture and genomic stability during neurogenesis (Atkins et al., 2020; Horváth et al., 2020). The gene expression profile mirrors Cluster 6 and suggests premature activation followed by later exhaustion of inhibitory-synapse genes (Figure 2B). A feedforward neural network trained on the 2,000 most variable genes reproduced stage calls with all WT samples on the diagonal of true versus predicted stage (Figure 2G). This model suggests that the R270X line exhibited the most divergent alignment from the WT trajectory, particularly in the NE stage. Convergence across k-means, random forest, and neural network indicates that the developmental stage still dominates variance, yet *MECP2* mutations superimpose an early surge and later collapse of neuronal and synaptic transcripts. This temporal misalignment helps automated models flag mutant ESCs and underscores the need to dissect transcriptional disruption at the blastocyst-equivalent stage.

### MECP2 loss promotes a naïve-like transcriptional program and increased proliferation in human ESCs

**Figure 3:**
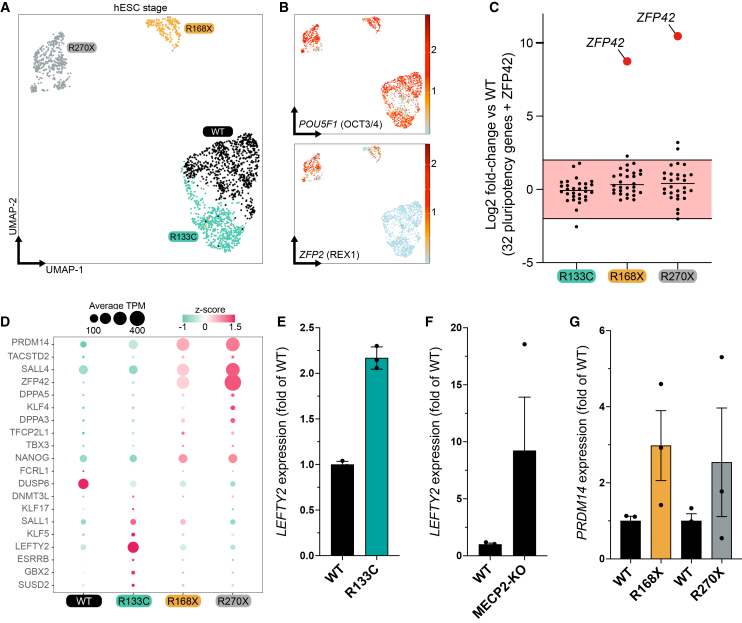
MECP2 loss drives a naïve-like transcriptional shift and hyperproliferation in human ESCs (A) UMAP projection of 2,925 single hESC transcriptomes colored by genotype (WT, R133C, R168X, R270X) at the pluripotent stage. (B) Feature plots show the per-cell log-normalized expression of the core pluripotency factor *POU5F1*/OCT4 (top) and the naïve-associated marker *ZFP42*/*REX1* (bottom) on the same UMAP embedding. (C) Dot plot summarizes log_2_ fold-change (mutant vs. WT) for a panel of 32 canonical pluripotency genes (black dots); red dots highlight *ZFP42* values. Computed from the bulk RNA sequencing matrix. (D) Dot plot displays average expression (dot size) and *Z* score (color scale) of the 20 naïve-enriched stem-cell markers across WT and mutant hESCs. Computed from the bulk RNA sequencing matrix. (E) Quantitative RT-PCR validation of *LEFTY2* expression in patient-derived isogenic iPSC lines expressing either *MECP2-WT* or *MECP2-R133C* transcripts. Bars represent mean ± s.e.m. (F) Quantitative RT-PCR validation of *LEFTY2* expression in a WT iPSC line and its isogenic line KO for *MECP2* alleles. Bars represent mean ± s.e.m. (G) Quantitative RT-PCR validation of *LEFTY2* expression in patient-derived isogenic iPSC lines expressing either *MECP2-WT*, *MECP2-R168X,* or *MECP2-R270X* transcripts. Bars represent mean ± s.e.m.

To determine whether the early transcriptional disruption at the pluripotent stage arises from heterogeneous differentiation states, we performed single-cell RNA sequencing on undifferentiated hESCs carrying WT, R133C, R168X, or R270X MECP2 alleles. UMAP embedding revealed four genotype-specific clusters: WT and R133C cells co-localized, whereas R168X and R270X formed distinct groupings that recapitulate our bulk RNA-seq observations (Figure 3A); the absence of subclusters within each genotype supports cellular homogeneity (Figure 3A). The expression of the core pluripotency factor *POU5F1* was uniform, yet truncating mutants (R168X and R270X) exhibited striking upregulation of *ZFP42*/REX1 (Figure 3B). ZFP42 encodes a zinc-finger protein primarily expressed in undifferentiated stem cells (Masui et al., 2008; Shi et al., 2006) and is enriched in naive relative to primed pluripotency, while remaining detectable at lower levels in primed ESCs (Ghimire et al., 2018; Marks et al., 2012; Takahashi et al., 2017; Theunissen et al., 2016). Consistent with this, WT hESCs show low but measurable *ZFP42* expression, whereas R168X and R270X display a marked increase that is reproduced in two independent CRISPR-edited clones (Figures S3A and S3B). A survey of 32 canonical pluripotency genes revealed no significant dysregulation, excluding a broad shift in pluripotency identity (Figure 3C). InferCNV analysis of the single-cell transcriptomes detected no copy-number alterations, and long-read whole-genome sequencing confirmed that all lines retain the expected on-target genotype (Figures S4A and S5). Thus, elevated *ZFP42* in truncating mutants is best interpreted as a naïve-like transcriptional bias within an otherwise intact pluripotent state, rather than as evidence of pluripotency loss or genomic instability.

We next asked whether this naïve-like signature extends beyond ZFP42. Interrogation of the top 20 naïve-enriched markers revealed that most are upregulated in *MECP2*-mutant hESCs, with *PRDM14* and *SALL4* particularly elevated in R168X and R270X, while R133C selectively increases a distinct subset of naïve-associated genes (Figure 3D). To test whether these changes generalize across pluripotent backgrounds, we examined naive markers in additional iPSC models. *LEFTY2*, a naïve-associated TGFβ ligand, was increased in R133C relative to its isogenic WT control and similarly elevated in an independent iPSC line in which *MECP2* was knocked out by CRISPR (Figures 3E and 3F). *PRDM14* was likewise upregulated in iPSCs carrying R168X or R270X relative to their respective isogenic controls (Figure 3G). Together, these data indicate that loss of *MECP2*, particularly truncating alleles, reproducibly induces a partial naïve-like transcriptional drift across distinct human pluripotent contexts.

To functionally probe naive identity, we transferred WT and mutant hESCs from standard mTeSR medium to a LIF-based, FGF/TGFβ-free medium that supports self-renewal of bona fide naive human PSCs. Under these conditions, neither WT nor mutant colonies maintained long-term self-renewal: Cells rapidly lost compact morphology and differentiated in all genotypes (Figures S3C and S3D). This confirms that *MECP2*-mutant hESCs do not fulfill functional criteria for naive pluripotency. Finally, live-cell imaging of colony expansion revealed that all three *MECP2*-mutant lines proliferate faster than WT over 72 h, whereas DNA-content flow cytometry showed broadly similar G0/G1, S, and G2/M fractions across genotypes (Figures S4B and S4C). Thus, M*ECP2* loss in hESCs is associated with a modest increase in proliferative capacity and a biased, but incomplete, acquisition of naïve-like transcriptional features, highlighting ZFP42 and related markers as focal nodes for mechanistic investigation.

### Convergent EMX1 repression in MECP2-mutant stem cells associates with cortical lineage imbalance

**Figure 4:**
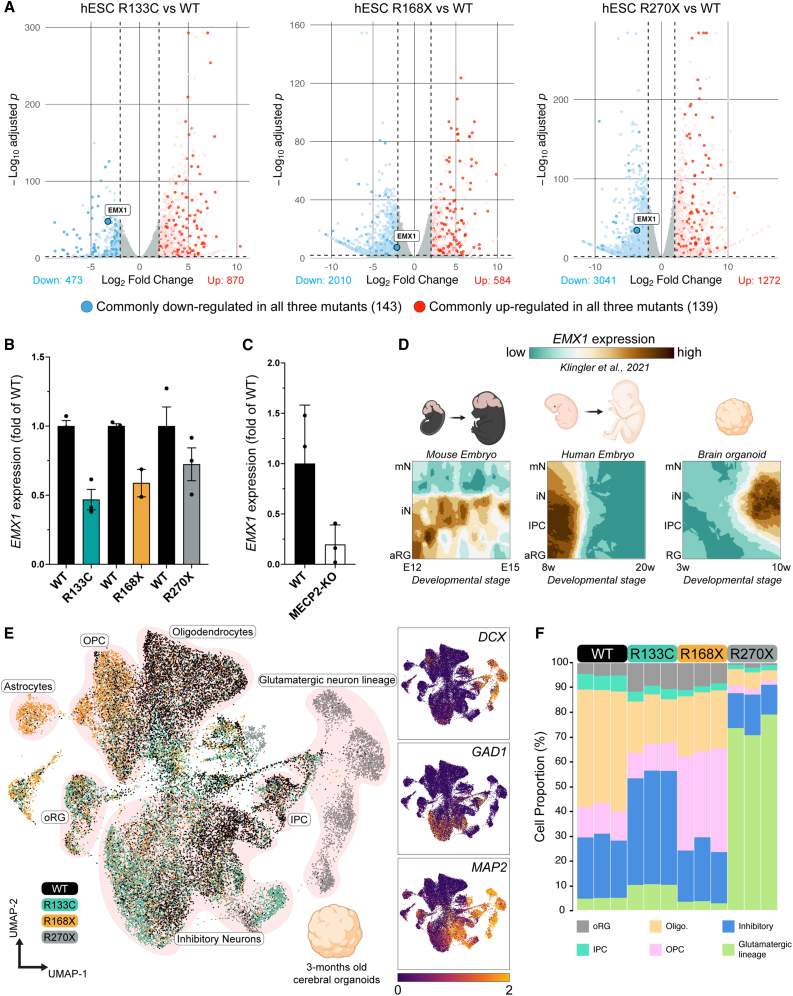
Convergent EMX1 repression links early defects to reduced excitatory-neuron output in Rett organoids (A) Volcano plots of differential expression (log_2_ fold-change vs. –log_10_ adjusted p) for each mutant hESC line relative to WT. Genes down-regulated in all three mutants are highlighted in blue (*n* = 143) and up-regulated genes in red (*n* = 139). The cortical progenitor determinant *EMX1* is indicated in each plot. Computed from the bulk RNA sequencing matrix. (B) Quantitative RT-PCR validation of *EMX1* expression in patient-derived isogenic iPSC lines expressing either *MECP2-WT*, *MECP2-R133C*, *MECP2-R168X,* or *MECP2-R270X* transcripts. Bars represent mean ± s.e.m. (C) Quantitative RT-PCR validation of *EMX1* expression in a WT iPSC line and its isogenic line KO for *MECP2* alleles. Bars represent mean ± s.e.m. (D) Reference single-cell atlases show the spatial localization of *EMX1* transcripts (teal-brown scale) in mouse embryonic cortex (E12–E15), human fetal cortex (8–20 weeks), and 10-week brain organoids. aRG, apical radial glia; IPC, intermediate progenitor cell; iN, immature neuron; mN, mature neuron. (E) Left, UMAP embedding of 25,132 single nuclei from three-month cerebral organoids (*n* = 3 per genotype), colored by genotype and annotated into six major populations: outer radial glia (oRG), intermediate progenitor cells (IPC), excitatory neurons, inhibitory neurons, oligodendrocyte, oligodendrocyte progenitor cells (OPC), and astrocytes. Right, feature plots of lineage markers *DCX* (excitatory), *GAD1* (inhibitory), and *MAP2* (pan-neuronal). (F) Stacked bar chart displays the proportional composition of each cell type per organoid across WT and the three mutant genotypes.

To pinpoint early, mutation-shared transcriptional defects that could prefigure downstream developmental abnormalities, we first performed bulk differential expression analysis on undifferentiated hESCs carrying the R133C, R168X or R270X *MECP2* alleles. This comparison yielded two compact, convergent gene sets with 143 transcripts down-regulated and 139 up-regulated in all mutants relative to WT (Figure 4A). Among the most strongly repressed genes was *EMX1*, a homeobox transcription factor that specifies dorsal telencephalic progenitors (Figure 4A) (Chou et al., 2009; O’Leary et al., 2007). Quantitative RT-PCR at the ESC stage confirmed significant *EMX1* reduction in all three mutant lines relative to WT, and this pattern was reproduced in two independent CRISPR-edited hESC clone series (Figures 4B and S6). *EMX1* expression was likewise decreased in an isogenic iPSC line in which *MECP2* was fully knocked out (Figure 4C), indicating that *EMX1* downregulation is a recurrent feature of *MECP2* loss across distinct human pluripotent backgrounds. To place EMX1 within a developmental framework, we mined reference single-cell atlases spanning mouse embryonic cortex (E12–E15), human fetal cortex (8–20 weeks), and human brain organoids (10 weeks) (Klingler et al., 2021). In all three systems, *EMX1* transcripts are restricted to apical radial glia and nascent glutamatergic neurons, positioning *EMX1* near the apex of cortical excitatory lineage specification (Figure 4D). This observation prompted us to ask whether early EMX1 dysregulation is accompanied by altered lineage allocation at later stages. We therefore generated three-month unguided cerebral organoids from each genotype and profiled them using single-nucleus RNA sequencing, an age when glutamatergic and GABAergic neurons, as well as multiple glial lineages, are readily detectable. UMAP projection separated nuclei into six canonical populations (outer radial glia (oRG), intermediate progenitor cells (IPCs), oligodendrocyte lineage, oligodendrocyte progenitor cells (OPCs), glutamatergic neuron lineage, and inhibitory neurons) validated by the expression of established markers including *SATB2*, *DCX*, *SLC17A7*, *NEUROD2/6*, *TBR1*, *GAD1,* and *PDGFRA* (Figures 4E and S7). Cell-type quantification revealed pronounced, mutation-specific shifts in lineage allocation compared with WT (Figure 4F). Control organoids were dominated by oligodendrocyte-lineage and OPC populations, with relatively modest fractions of inhibitory and glutamatergic neuron lineage cells. In contrast, R133C organoids exhibited an expanded inhibitory compartment and reduced oligodendroglial representation; R168X organoids accumulated OPC/oligodendrocyte progenitors while retaining very few glutamatergic lineage cells; and R270X organoids showed a marked predominance of glutamatergic neuron lineage cells with comparatively sparse glial populations. These data indicate that *MECP2* mutations perturb cortical cell-type balance in distinct, allele-specific patterns. Together with the convergent *EMX1* repression observed in pluripotent cells, these findings delineate a coherent trajectory in which early MECP2-dependent transcriptional lesions, including mis-timed *EMX1* expression, are associated with biased cortical lineage outcomes in Rett-associated genotypes.

## Discussion

By integrating stage-resolved bulk and single-cell transcriptomics with functional assays in isogenic human ESCs, Rett patient-derived iPSCs, and long-term cerebral organoids, we identify a shared, MECP2-dependent transcriptional program that is already perturbed at the pluripotent stage. Three convergent features emerge. First, a discrete set of 282 transcripts is consistently mis-regulated across three representative *MECP2* mutations, indicating that MECP2 influences a restricted yet coherent gene network even before neural induction. Second, truncating alleles (R168X, R270X) elicit a partial naïve-like molecular signature, marked by *ZFP42*, *PRDM14,* and *SALL4* elevation and accompanied by increased proliferative capacity, without full conversion to bona fide naive pluripotency. Third, all mutants display an abnormal developmental trajectory of the dorsal telencephalic determinant *EMX1*, with early repression in pluripotent cells and altered expression at later precursor stages, and these early lesions are associated with mutation-specific shifts in glutamatergic, inhibitory, and glial lineage proportions in three-month cerebral organoids. Collectively, our data situate the origin of RTT-relevant defects at the pluripotent/early progenitor stages and outline trajectories through which early transcriptional noise may culminate in cortical-circuit imbalance.

### Early transcriptional disruption and its developmental implications

The detection of genotype-specific variance on principal component 2 as early as the ESC stage (Figure 1B) indicates that MECP2 influences transcription prior to neural induction, in a context where global DNA methylation levels are lower than in differentiated neurons. This is consistent with recent reports that MECP2 can bind enhancer-like hotspots and hypomethylated regions, but our study does not directly measure DNA methylation or MECP2 occupancy and therefore cannot distinguish methylation-dependent from methylation-independent mechanisms (Liu et al., 2024; Mishra et al., 2025). The convergent mis-regulation of S100A6 and SLC17A7 across all stages (Figure 1C) further suggests that cytoskeletal organization, cell-state stability, and glutamatergic signaling are primed for later dysfunction before lineage commitment occurs. Our machine-learning classifiers, trained on bulk RNA-seq trajectories, readily decoded developmental stage and highlighted a subset of neuronal genes whose expression followed an “early-low-late-high” pattern in mutants compared with WT (Figures 2 and S2), suggesting mistimed engagement of neurogenic programs. Such temporal misalignment may help reconcile seemingly contradictory findings in mouse models in which *Mecp2* loss produces both premature and delayed aspects of neuronal maturation (Chen et al., 2015; Smrt et al., 2007). A full understanding of these dynamics will require time-resolved chromatin profiling and base-resolution methylome maps in the same lines, ideally coupled to MECP2 ChIP/CUT&Tag, to test whether the observed transcriptomic shifts reflect direct MECP2 binding or secondary remodeling of the epigenetic landscape.

### A partial shift toward a naïve-like pluripotent state

The up-regulation of *ZFP42*, *PRDM14*, *TACSTD2,* and other naïve-enriched markers, together with the increased growth rate of mutant colonies, points to a partial drift from a purely primed toward a more naïve-like pluripotent state. Naive human PSCs are characterized by the elevated expression of these factors, altered metabolic profiles, and distinct epigenetic features, including global DNA hypomethylation and reduced H3K27me3 deposition (Theunissen et al., 2016). In our models, we observe robust induction of *ZFP42*/REX1 and *PRDM14* in truncating mutants, reproducible across two independent CRISPR-edited hESC clones and mirrored in *MECP2*-KO and mutant iPSC lines (Figures 3, S3, and S6), as well as a modest but consistent proliferation advantage (Figure S4C). At the same time, canonical pluripotency factors remain stable, inferCNV and long-read WGS reveal no recurrent structural abnormalities (Figures S4 and S5), and *MECP2*-mutant hESCs fail to self-renew in LIF-based, FGF/TGFβ-free conditions that support bona fide naive PSCs (Figures S3C and S3D). Taken together, these observations argue that *MECP2* loss induces a naïve-like transcriptional bias within an otherwise intact pluripotent framework, rather than triggering a complete reset to a ground-state pluripotency. Whether this bias reflects a cell-autonomous role of MECP2 in stabilizing the primed state, or arises from subtle culture-selection advantages conferred by increased proliferation, remains to be determined. Future work should combine functional assays of naive competence (e.g., trophectoderm potential, transposon activation profiles), global DNA/histone methylation measurements, and single-cell lineage tracing to refine how MECP2 shapes the primed-naïve spectrum in human pluripotent cells.

### EMX1 as an early bottleneck for cortical excitatory fate

Among the 143 convergently down-regulated genes, *EMX1* stands out because of its highly restricted expression in apical radial glia and nascent glutamatergic neurons across mouse, human fetal cortex, and organoid atlases (Figure 4D). In our system, *EMX1* transcripts are reduced in *MECP2*-mutant hESCs and in two independent CRISPR-edited clone series, as well as in *MECP2*-KO iPSCs (Figures 4B, 4C, and S6), and follow an abnormal neuronal trajectory compared with WT. Although our current data are limited to mRNA, the consistency of *EMX1* repression across alleles and pluripotent backgrounds suggests that EMX1 lies within a core MECP2-dependent developmental program. Single-nucleus RNA-seq in three-month cerebral organoids further shows that MECP2 mutations are associated with allele-specific but convergent perturbations of cortical lineage composition, with altered proportions of glutamatergic neuron lineage, inhibitory neurons, and oligodendrocyte-lineage cells (Figures 4E, 4F, and S7). These findings are compatible with mouse studies in which *Emx1* loss biases cortical progenitors toward ventral or glial fates (Shinozaki et al., 2002) but do not by themselves prove that EMX1 mediates the organoid phenotypes. We therefore view EMX1 as a candidate early bottleneck for cortical excitatory fate in *MECP2*-deficient cells. Demonstrating causality will require stage-specific *EMX1* gain- and loss-of-function in *MECP2*-mutant ESCs and organoids, coupled to protein-level readouts and long-term lineage tracing. Single-cell multi-omics combining chromatin accessibility, MECP2 occupancy, and EMX1 transcription will also be important to distinguish direct from indirect regulation.

### Translational outlook

On the therapeutic front, the partial naïve-like drift and early EMX1 dysregulation raise the possibility that interventions targeting chromatin state or lineage-bias pathways might complement post-natal gene-replacement strategies. Epigenetic drugs or small molecules that stabilize primed pluripotency, modulate MECP2-regulated enhancer networks, or fine-tune EMX1 and related cortical fate determinants could, in principle, normalize aspects of lineage allocation if applied within appropriate developmental windows. Whether such approaches are feasible and safe *in vivo* remains an open question, particularly given the challenges of timing, mosaic X-inactivation in female patients, and potential off-target effects on other brain regions. Our work provides a framework and candidate molecular nodes (naïve-like regulators and EMX1) for future mechanistic and preclinical studies.

### Limitations of the study

The concept that neurodevelopmental disorders originate from perturbations in pluripotent or early progenitor states is gaining traction in Fragile X, 22q11.2 deletion syndrome, and autism spectrum disorder models (Ardhanareeswaran et al., 2017; C. Li et al., 2023; Schafer et al., 2019). Our findings position RTT within this framework and suggest that therapeutic windows may extend into very early developmental stages. However, translating these insights to patients will require caution. Our work relies on human ESCs, iPSCs and cerebral organoids, which offer genetically controlled systems but do not fully reproduce the *in vivo* environment. Morphogen gradients, vascularization, long-range connectivity, and systemic influences are only partially modeled, and our organoid analyses were performed at a single late time point (three months). Additional time points and *in vivo* validation in models carrying the same *MECP2* alleles will be important to confirm how early the described defects arise and how they evolve over development.

A second limitation is that many of our conclusions are based on transcriptomic readouts. We did not yet combine these datasets with genome-wide measurements of DNA and histone modifications or direct MECP2 occupancy, so the precise epigenetic mechanisms underlying the observed changes remain unresolved. Likewise, EMX1 is currently supported as a candidate early bottleneck for cortical excitatory fate by convergent mRNA-level data across *MECP2*-mutant ESC and iPSC models, including lines with controlled X chromosome inactivation, but we have not yet performed stage-specific EMX1 gain- or loss-of-function, protein-level quantification, or multi-omic assays that would establish causality and direct regulation.

Finally, the naïve-like drift and lineage imbalances we describe are inferred from transcriptional signatures, growth kinetics, and snRNA-seq-based cell-type annotation, without the systematic validation of naive functional properties or quantitative immunohistochemistry on matched organoids. Future studies combining epigenomic profiling, protein-level analyses, functional rescue experiments, and electrophysiological characterization will be required to fully define how early MECP2-dependent lesions translate into cortical circuit dysfunction in RTT.

## Resource availability

### Lead contact

Lead contact: Anthony Flamier, anthony.flamier@umontreal.ca.

### Materials availability

All unique, stable reagents generated in this study are available from the lead contact upon reasonable request, contingent on the completion of a Materials Transfer Agreement.

### Data and code availability

• Genomic data are available through a public repository (bulk RNAseq: GSE303838; scRNAseq: GSE303813; snRNAseq: GSE303977).
• All other raw data and codes are available upon request to the lead contact.

## Acknowledgments

We thank Dr. Rudolf Jaenisch and his team for sharing key reagents and providing strategic guidance. We thank Drs. Graziella Di Cristo, Elsa Rossignol, and Serge McGraw for their insightful guidance; Basma Benabdallah and the CHU Sainte-Justine iPSC core facility; and Nicholas Geoffrion and the bioinformatics core facility for essential technical support. This work was funded by the Canada Brain Research Fund (CBRF)—a partnership between Health Canada and the 10.13039/100009408Brain Canada Foundation (Future Leaders Program); the 10.13039/501100005155Azrieli Foundation; the 10.13039/501100000024Canadian Institutes of Health Research; the Canadian Stem Cell Network Jump Start ECR Program; the 10.13039/501100023447CHU Sainte-Justine Foundation; the Fonds de Recherche du Québec-Santé (10.13039/501100000156FRQS); and the 10.13039/100016499Rett Syndrome Research Trust (for providing Rett iPSC lines). Additional support was provided by the Fonds UdeM pour le partenariat CHU Sainte-Justine -Institut Imagine en épilepsie de l’enfant. We also thank the International Rett Syndrome Foundation (IRSF) Research Independence Award (to Y.L.).

## Author contributions

A.F. conceived the study, secured funding, and provided overall scientific leadership and project coordination (conceptualization, funding acquisition, project administration). M.G., M.B. (Guillon), J.G., L.L. (Laurent), T.R., E.G., M.B. (Brin), J.K., A.B., L.B., M.V., and A.F. each made substantial contributions to the generation, curation, and quality control of the datasets and to the execution of the experimental work; collectively, they performed the majority of wet-lab experiments, validated key findings, and produced the primary data visualizations and figure materials (investigation, data curation, validation, visualization). Y.L. provided essential reagents and cell resources, maintained and quality-controlled cell lines, and contributed directly to experimental execution (resources, investigation). L.L. (Laurent), L.A., and A.F. provided day-to-day scientific oversight, supervised personnel and experimental workflows, and contributed critical interpretation and strategic guidance throughout the study (supervision). A.F. drafted the manuscript, and all authors contributed to manuscript development by critically revising the text, figures, and interpretation, and approving the final submitted version (writing – original draft; writing – review and editing). All authors read and approved the manuscript.

CRediT taxonomy: conceptualization: A.F. methodology: A.F.; M.G.; M.B. (Guillon); J.G.; L.L. (Laurent); T.R.; E.G.; M.B. (Brin); J.K.; A.B.; L.B.; M.V.; Y.L.; L.A. investigation: M.G.; M.B. (Guillon); J.G.; L.L. (Laurent); T.R.; E.G.; M.B. (Brin); J.K.; A.B.; L.B.; M.V.; Y.L.; A.F. resources: Y.L.; A.F. data curation: M.G.; M.B. (Guillon); J.G.; L.L. (Laurent); T.R.; E.G.; M.B. (Brin); J.K.; A.B.; L.B.; M.V.; A.F. formal analysis: A.F.; M.G.; M.B. (Guillon); J.G.; L.L. (Laurent); T.R.; E.G.; M.B. (Brin); J.K.; A.B.; L.B.; M.V. validation: M.G.; M.B. (Guillon); J.G.; L.L. (Laurent); T.R.; E.G.; M.B. (Brin); J.K.; A.B.; L.B.; M.V.; A.F. visualization: M.G.; M.B. (Guillon); J.G.; L.L. (Laurent); T.R.; E.G.; M.B. (Brin); J.K.; A.B.; L.B.; M.V.; A.F. supervision: A.F.; L.L. (Laurent); L.A. project administration: A.F. funding acquisition: A.F. writing – original draft: A.F. writing – review and editing: All authors.

## Declaration of interests

A.F. is a co-founder and shareholder of StemAxon. The other authors declare no competing interests.

## Declaration of generative AI and AI-assisted technologies in the writing process

During the preparation of this work, the authors used OpenAI ChatGPT Large Language Model o3 to enhance the clarity of the text. The authors reviewed and edited the content and take full responsibility.

## STAR★Methods

### Key resources table

**Table:** 

REAGENT or RESOURCE	SOURCE	IDENTIFIER
**Antibodies**		

Anti-GAPDH antibody EPR16891	Abcam	ab181602; RRID: AB_2630358
Donkey Anti-Mouse IgG H&L (HRP)	Abcam	ab205724
Donkey Anti-Rabbit IgG H&L (HRP)	Abcam	ab205722; RRID: AB_2904602
MAP2 (D5G1) Rabbit Monoclonal Antibody	Cell signaling	8707; RRID: AB_2722660
Nestin (E4O9E) Rabbit Monoclonal Antibody	Cell signaling	73349
Purified anti-Oct4 (Oct3) Antibody	Bio Legend	653702; RRID: AB_2561767

**Chemicals, peptides, and recombinant proteins**		

Complete Cell Media w/15% FBS Serum and LIF	Sigma-Aldrich	ES-101-B
Corning Matrigel Basement Membrane Matrix, LDEV-free	Corning	354234
Corning Matrigel Growth Factor Reduced (GFR) Basement Membrane Matrix, Phenol Red-free, LDEV-free	Corning	356231
Dimethyl sulfoxide	Sigma-Aldrich	D8418
DMEM/F12	Wisent Inc	319-090 CL
Gentle Cell Dissociation Reagent	Stem Cell Technologies	100-0485
KnockOut Serum Replacement	Gibco	10828010
mTeSR Plus	Stem Cell Technologies	100-0276
mTeSR Plus Supplement	Stem Cell Technologies	100-0275
Pen Strep Glutamine (100X)	Gibco	10378-016
Plasmocin Prophylactic	InvivoGen	ant-mpp
PowerUp SYBR Green Master Mix for qPCR	Applied Biosystems	A25742
QIAshredder	Qiagen	79656
qScript cDNA SuperMix	Quantabio	95048-100
ReleSR	Stem Cell Technologies	100-0483
STEMDiff SMADI Neural Induction Supplement	Stem Cell Technologies	08580
STEMDiff Neural Induction	Stem Cell Technologies	05835
Trypan blue Trypan blue	Thermo Fisher	T10282
UltraPure DNase/RNase-Free Distilled Water	Invitrogen™	10977-015
Y-27632 (Dihydrochloride)	Stem Cell Technologies	72304

**Critical commercial assays**		

Cell Cycle Analysis Kit	Abcam	ab287852
DNA 1000 Kit	Agilent Technologies	5067-1504
EVERCODE Cell Fixation v2	Parse Bioscience Inc	ECF2101
EVERCODE Nuclei Fixation v2	Parse Bioscience Inc	ECF2103
EVERCODE WT Mini v2	Parse Bioscience Inc	ECW02115
EVERCODE WT v2	Parse Bioscience Inc	ECW02135
NEBNext High Input Poly(A) mRNA Isolation Module	New England Bioloabs	E3370S
NEBNext Ultra II Directional RNA Library Prep Kit for Illumina	New England Bioloabs	E7760L
PureLink RNA Mini Kit	Thermo Fisher Scientific	12183018A
QuantiFluor dsDNA system	Promega Corporation	E2670
RNeasy Mini Kit	Qiagen	74106
STEMDiff Cerebral Organoid Kit	Stem Cell Technologies	08570

**Oligonucleotides**		

MECP2-KO sgRNA target sequences - forward	Millipore-Sigma	CATCATACATGGGTCCCCGG
MECP2-KO sgRNA target sequences - reverse	Millipore-Sigma	CCGAGTCTCTGTTGTCCTGG
qPCR_EMX1_F	Millipore-Sigma	ACCGGGACCCTCTCCATTT
qPCR_EMX1_R	Millipore-Sigma	GCTTCTGCCGTTTGTACTTTGT
qPCR_ZFP42_F	Millipore-Sigma	AGAAACGGGCAAAGACAAGAC
qPCR_ZFP42_R	Millipore-Sigma	GCTGACAGGTTCTATTTCCGC
qPCR_XIST_F	Millipore-Sigma	CCAGGCAATCTGCTCTGGAA
qPCR_XIST_R	Millipore-Sigma	ATGCTGACTACCCAAAGCCC
qPCR_POU5F1_F	Millipore-Sigma	CCCCAGGGCCCCATTTTGGTACC
qPCR_POU5F1_R	Millipore-Sigma	ACCTCAGTTTGAATGCATGGGAGAGC
qPCR_RPLP0_F	Millipore-Sigma	AGCCCAGAACACTGGTCTC
qPCR_RPLP0_R	Millipore-Sigma	ACTCAGGATTTCAATGGTGCC
qPCR_LEFTY2_F	Millipore-Sigma	GGCCCAGTATGTAGTCCTGC
qPCR_LEFTY2_R	Millipore-Sigma	TCCATGCCGAACACCAGC
qPCR_PRDM14_F	Millipore-Sigma	AATCATTGGTGGCGACAACGA
qPCR_PRDM14_R	Millipore-Sigma	CCCGTACAGAACGAAGTGCAG

**Guide RNAs**		

KO-MECP2 sgRNA-1	EditCo	UGGUGGGCUGAUGGCUGCAC
KO-MECP2 sgRNA-2	EditCo	UCUUCACCUUUUUAAACUUG
KO-MECP2 sgRNA-3	EditCo	GGAAGAAAAGUCAGAAGACC

**Deposited data**		

RNA sequencing	This study	GEO: GSE303838
Single-nucleus RNA sequencing	This study	GEO: GSE303977
Single-cell RNA sequencing	This study	GEO: GSE303813

**Experimental models: Cell lines**		

hESC-WIBR1 R133C MECP2-GFP	Liu et al., (2024)	N/A
hESC-WIBR1 R168X MECP2-GFP	Liu et al., (2024)	N/A
hESC-WIBR1 R270X MECP2-GFP	Liu et al., (2024)	N/A
hESC-WIBR1 WT MECP2-GFP	Liu et al., (2024)	N/A
hiPSC PGP-1	EditCo	N/A
hiPSC WT-M	Wogram et al., (2025)	N/A
hiPSC WT-F	This study	N/A
hiPSC p.R133C	RSRT iPSC Collection Coriell	N/A
hiPSC p.R168X	RSRT iPSC Collection Coriell	N/A
hiPSC p.R270X	RSRT iPSC Collection Coriell	N/A

**Software and algorithms**		

DESeq2	Love et al., (2014)	https://bioconductor.org/packages/release/bioc/html/DESeq2.html; RRID:SCR_015687
GraphPad Prism GraphPad Software V.10	N/A	https://www.graphpad.com/scientific-software/prism/; RRID:SCR_002798
Fiji image processing package.76	Schindelin et al., (2012)	http://fiji.sc; RRID:SCR_002285
featureCounts	Liao et al., (2014)	https://subread.sourceforge.net/featureCounts.html; RRID:SCR_012919
STAR	Dobin et al., (2013)	https://hbctraining.github.io/Intro-to-rnaseq-hpc-O2/lessons/03_alignment.html; (RRID:SCR_004463)
FASTQC	N/A	https://www.bioinformatics.babraham.ac.uk/projects/fastqc/; (RRID:SCR_014583)
SAMtools	Li et al., (2009)	https://www.htslib.org/; RRID:SCR_002105
Seurat V.5	Hao et al., (2024)	https://satijalab.org/seurat/; (RRID:SCR_016341)
Trim Galore!	N/A	https://github.com/FelixKrueger/TrimGalore; RRID:SCR_011847
clusterProfiler	Yu et al., (2012)	https://bioconductor.org/packages/devel/bioc/html/clusterProfiler.html; (RRID:SCR_016884)
RandomForest Package in R	Breiman (2001)	https://cran.r-project.org/web/packages/randomForest/index.html; (RRID:SCR_015718)
Keras	N/A	https://github.com/rstudio/keras; (RRID:SCR_026159)
TensorFlow	N/A	https://cran.r-project.org/web/packages/tensorflow/index.html; (RRID:SCR_016345)

### Experimental model and study participant details

This study used established human pluripotent stem cell (hPSC) lines and did not recruit living human participants. Three CRISPR/Cas9-edited male WIBR1 hESC lines carrying *MECP2* Rett-associated variants (R133C, R168X, R270X) were analyzed alongside the isogenic WT parental line. Three female patient-derived iPSC lines harboring distinct *MECP2* variants (R133C, R168X, R270X) were obtained from the RSRT iPSC Collection (Coriell Institute). In addition, two control iPSC lines, one male and one female, were included. The experimental design therefore comprised a male isogenic hESC series together with female patient-derived iPSC lines and male and female control iPSC lines (hiPSC WT-M and hiPSC WT-F). The study was not powered or structured to formally test sex- or gender-associated differences in outcomes; therefore, sex-dependent effects could not be systematically assessed and represent a limitation for generalizability beyond the specific lines analyzed. All experimental procedures involving hPSC lines were reviewed and approved by institutional ethics committee (CHU Sainte-Justine).

### Method details

#### Pluripotent stem cell culture

Human embryonic stem cells (hESCs) WIBR1 were maintained under feeder-free conditions. Three CRISPR/Cas9-edited male hESC lines carrying the recurrent Rett-syndrome mutations R133C, R168X, and R270X were used alongside the isogenic wild-type (WT) parental line as previously reported (Liu et al., 2024). In addition, three female patient-derived induced pluripotent stem cell (iPSC) lines harbouring distinct *MECP2* variants (R133C, R168X, R270X) were obtained from the RSRT iPSC Collection (Coriell Institute). For routine culture, cells were thawed onto tissue-culture plates coated with hESC-qualified Matrigel (Corning, #354277) and maintained in mTeSR Plus medium (Basal Medium #100-0274 supplemented 1:5 with mTeSR Plus 5× Supplement, #100-2075; STEMCELL Technologies). Cultures were incubated at 37°C in 5 % CO_2_ and medium was refreshed every 24 h. Cells were passaged every 3–4 days using ReLeSR (STEMCELL Technologies, #100-0483); 10 μM Y-27632 ROCK inhibitor (STEMCELL Technologies, #72308) was added for 24h after passaging to enhance survival. All cell lines were used between passages 20-35 and cultured at a split ratio of 1:6 to 1:10. Freezing and thawing was done using mFreSR reagent (STEMCELL Technologies, #05855) according to manufacturer protocol. Cell lines were monitored daily for evidence of visible contamination and tested every 10 passages for mycoplasma contamination (PCR assay).

#### Generation of MECP2 KO iPS cells

PGP-1 iPS cells have been transfected with RNP complex using a mix of 3 gRNA from Editco according to their protocol. Briefly, RNP complexes were assembled during 10 min at room temperature with 7.5:1 sgRNA to Cas9 ratio (i.e. 250pmol sgRNA and 34pmol Cas9). Then 500.000 PGP-1 iPS cells were mixed with the RNP in Lonza P3 Nucleofector solution (Lonza; #V4XP-3032) and transfected using the 4D-nucleofector. Cells were then transferred in a 6-well plate coated with Matrigel hESC (corning; #354277) with mTeSR supplemented with rock inhibitor (10μM). Media was change 24-hr post nucleofection and cells were incubated for 4 days.

DNA were extracted using Quick Extract solution (Mandel scientific; #QE0905T) and amplified by PCR using primers designed against regions of MECP2 flanking the sgRNA target sequences to generate an amplicon of 417 bp. PCR products were sequenced using Sanger Sequencing (Genome Quebec) and indels were identified using ICE analysis software (ICE CRISPR Analysis. 2025. v3.0. EditCo Bio).

#### Neuronal differentiation

hESCs were plated on three well of a 6-well plate coated with Growth Factor Reduced Matrigel using ReLeSR (STEMCELL Technologies, #100-0483). After 24h (around 70% confluence) cells were incubated with Neural induction media with SMAD inhibitors (STEMCELL Technologies, #08580) with daily full media change. Differentiated cells were stopped 3 days (Neuroectodermal stage), 7 days (Neural stem cell stage) and 21 days (Neural progenitor cell stage) post induction.

#### Cell-growth assay

Individual hESC lines were seeded onto 6-well plates pre-coated with hESC-qualified Matrigel (Corning, #354277). After a 24-h attachment period, plates were transferred to an Incucyte S3 live-cell imager and kept under standard culture conditions (37°C, 5 % CO_2_). Nine non-overlapping phase-contrast images per well were captured every 4 h for 72 h. For each well three independent colonies of comparable initial size across genotypes were manually selected, and its perimeter was measured at every time point in ImageJ. Growth curves were generated by plotting the perimeter of the same colony over the 72-h imaging window.

#### Unguided cerebral-organoid differentiation

Unguided cortical organoids were generated from WT, R133C, R168X and R270X hESCs with the STEMdiff Cerebral Organoid Kit (STEMCELL Technologies, #08570). Briefly, hESCs were dissociated to single cells with ReLeSR, counted, and seeded at 9 × 10^3^ cells per well in low-attachment, V-bottom 96-well plates supplied with the kit (day 0). Aggregates were cultured in Embryoid Body Medium for 5 days, with half-medium changes on days 2 and 4, to promote uniform spheroid formation. On day 5, spheroids were transferred to neural induction medium in ultra-low-attachment 24-well plates (three spheroids per well). After 48 h (day 7), each spheroid was embedded in a 30-μl dome of growth-factor-reduced Matrigel and moved to maturation medium in a Celtron orbital shaker (INFORS HT, #I69222) operating at 75 rpm inside a humidified incubator (37°C, 5 % CO_2_). Medium was exchanged every 3–4 days for the duration of the culture. To limit central hypoxia and necrosis, organoids were sectioned under sterile conditions beginning at day 60. Using a Leica VT1000 S vibratome fitted with a sterile blade, each organoid was bisected or trisected (300–400 μm slices) every 2–3 weeks and individual fragments were returned to the shaker in fresh maturation medium. Cultures were harvested at 4 months for downstream single-nucleus RNA-seq and histological analyses.

#### Reverse-transcription quantitative PCR (RT-qPCR)

Total RNA was isolated from each hiPSC line in triplicate (n = 3 biological replicates per genotype) with the RNeasy Mini Kit (Qiagen, #74106) on a QIAcube automated workstation. For cDNA synthesis, 500 ng of RNA were reverse-transcribed using qScript cDNA SuperMix (Quantabio, #95048-100) on a SimpliAmp thermal cycler, following the manufacturer’s protocol. The resulting cDNA was diluted 1:8 with nuclease-free water and used as template for SYBR Green qPCR. Reactions (10 μL total) were assembled in 96-well plates as follows: 5 μL PowerUp SYBR Green Master Mix (Thermo Fisher Scientific), 0.5 μL forward primer (10 μM), 0.5 μL reverse primer (10 μM), 3 μL UltraPure DNase/RNase-free distilled water, and 1 μL diluted cDNA.

Thermal cycling conditions followed the Master Mix guidelines (two-step protocol with melt-curve analysis). All samples were run in technical triplicate, and relative expression was calculated by the ΔΔCt method after normalization to the geometric mean of *RPLP0*.

#### Bulk RNA sequencing

Total RNA was extracted in biological triplicate (n = 3 per line) with the PureLink RNA Mini Kit (Invitrogen, #12183018A) at four matched time points: day 0, day 3, day 7 and day 21 for WT and the three point-mutant lines. Polyadenylated RNA was purified from each triplicate with the NEBNext Poly(A) mRNA Magnetic Isolation Module (New England Biolabs, #E3370S). One microlitre of the eluate was quantified on a NanoDrop spectrophotometer. In total, 48 barcoded libraries were prepared with the NEBNext Ultra II Directional RNA Library Prep Kit for Illumina (NEB, #E7760L) according to the manufacturer’s instructions. Libraries were sequenced on Illumina NovaSeq X Plus instruments using paired-end 50-bp reads (PE50) with at least 40 million reads per sample.

#### Bulk RNA sequencing analysis

Raw paired-end FASTQ files were quality-checked with FastQC (v0.11.9) and summarised with MultiQC. Adapter sequences and bases with Phred < 20 were removed using Trimmomatic (v0.39; settings ILLUMINACLIP:2:30:10, SLIDINGWINDOW:4:20, MINLEN:36). Cleaned reads were aligned to the GRCh38/hg38 reference (Ensembl 109 annotation) with STAR (v2.7.11a) in two-pass mode (--twopassMode Basic) and sorted BAMs were produced (--outSAMtype BAM SortedByCoordinate). Gene-level counts were obtained with featureCounts (v2.0.2; options -p -B -C --primary -s 0) and imported into R (4.3.2). Low-abundance features (row-sums ≤ 1) were discarded, and a DESeq2 (v1.40.2) object was created with the design formula ∼ Genotype + Time. After dispersion estimation and Wald testing, contrasts were extracted for each mutant versus WT at each time point; genes with log_2_FC > 2 and Benjamini–Hochberg–adjusted p < 0.01 were considered differentially expressed. Variance-stabilised counts (vst, blind = FALSE) were used for principal-component analysis (plotPCA) and sample–sample correlation heat-maps (Pearson r, ggplot2 v3.4.4). Differential-expression result tables were exported per comparison, summarised, and filtered lists were subjected to Gene-Ontology over-representation analysis with clusterProfiler (v4.8.3; enrichGO, OrgDb = org.Hs.eg.db v3.17.0, ont = “ALL”, padj < 0.05). Transcript-per-million (TPM) values were calculated from raw counts and gene lengths (featureCounts “Length” column) and log_2_-transformed for marker-gene heat-maps (pheatmap v1.0.12, viridis palette). In Figure 3C, the 32 canonical pluripotency genes based on Ghosh et al., 2020 are: *C1orf210*, *CLDN6*, *ESRP1*, *GLB1L3*, *GYLTL1B*, *HES3*, *KCNG3*, *L1TD1*, *LCK*, *LIN28A*, *MATK*, *NANOG*, *POU5F1*, *PPP1R16B*, *PRDM14*, *PRSS8*, *SCNN1A*, *SLC7A3*, *TDGF1*, *TRIM71*, *VRTN*, *ZSCAN10*, *AC007326.1*, *APELA*, *BEND4*, *CAMKV*, *DPPA4*, *HLA-DOA*, *HTR3A*, *KCNK5*, *PTPRZ1*, *SYT6*.

#### Deep learning models

##### K-means clustering

Gene-level TPM values were imported into R (v4.3.2) and filtered to the 10 000 most variable transcripts across all samples (coefficient of variation). Expression values were log_2_-transformed and centred before clustering with the base kmeans() function, specifying k = 6, nstart = 25 and the default Euclidean distance. Cluster membership was merged with sample metadata for downstream visualisation of cluster-specific trajectories and Gene-Ontology enrichment.

##### Random-forest stage classifier

For supervised classification, only WT samples were used for training. The TPM matrix was transposed so that rows corresponded to samples and columns to genes; metadata columns (Sample, Genotype, Stage) were removed from the feature set. A factor response vector encoded the four developmental stages (ESC, NE, NSC, NPC). A random-forest model was trained with the randomForest package (v4.7-1.1) using ntree = 500, default mtry, and importance = TRUE. Model performance was inspected via the out-of-bag error rate and a confusion matrix. Variable importance (mean decrease in accuracy) was extracted and plotted for the top 25 genes. The trained classifier was applied to mutant samples (R133C, R168X, R270X) to obtain both hard stage calls and class-probability distributions; results were visualised with ggplot2 (v3.4.4). Mis-classifications were summarized and prediction confidence values (maximum class probability) were compared across genotypes.

##### Feed-forward neural-network classifier

A deep neural network was implemented with the keras R interface (keras v2.13.0; TensorFlow v2.14 backend). WT TPM values were log_2_(x + 1) transformed, gene-wise z-scored (mean subtraction, division by s.d.), and clipped to ±5. The network architecture comprised an input layer matching the number of genes, two hidden dense layers (64 and 32 ReLU units) each followed by 0.30 dropout, and a 4-unit soft-max output layer. Categorical stage labels were one-hot encoded. The model was compiled with categorical cross-entropy loss, the legacy Adam optimizer (learning rate = 5 × 10^-4^), and accuracy as the metric. Training proceeded for up to 200 epochs with batch_size = 4, using a 25 % validation split; early-stopping (patience = 10) and ReduceLROnPlateau (patience = 5) callbacks prevented over-fitting. After convergence, the model weights with the lowest training loss were retained. Mutant samples were pre-processed with the same scaling parameters and predicted to yield both class labels and confidence scores.

#### Single-cell RNA sequencing

Human ESCs (WT, R133C, R168X and R270X) were harvested at the pluripotent stage, dissociated with Gentle Cell Dissociation (Stem Cell Technologies) and resuspended in ice-cold PBS + 0.04 % BSA. For each genotype, cells from three independent wells were pooled, yielding four fixed suspensions in total. Fixation was performed immediately with the EVERCODE Cell Fixation v2 Kit (Parse Biosciences, #ECF2101) according to the manufacturer’s protocol. Cell density and viability were assessed by mixing 10 μL of the suspension with 10 μL 0.4 % Trypan Blue (Thermo Fisher, #T10282) and counting on a Countess 3 Automated Cell Counter; all samples exceeded 90 % viability and were adjusted to 1 × 10^6^ cells mL^-1^. Two single-cell libraries were constructed from the four fixed samples using the EVERCODE WT Mini v2 Kit (Parse Biosciences, #ECW02115), which employs a split-pool combinatorial indexing workflow comprising four rounds of barcoding followed by cDNA synthesis and amplifications. Final libraries were purified with AMPure XP beads, and double-stranded DNA concentration was measured fluorometrically with a Qubit 4 and the QuantiFluor dsDNA System (Promega, #E2670). Both libraries were sequenced on an Illumina NovaSeq 6000 S4 flow-cell using paired-end 150-bp reads (PE150).

#### Single-nucleus RNA sequencing

Cerebral organoids were harvested on day 90 from three independent cultures per genotype, and all reagents, consumables and centrifuges were pre-cooled to 4°C. Organoids were transferred to a chilled Dounce containing 700 μL homogenization buffer (NIM1: 250 mM sucrose, 25 mM KCl, 5 mM MgCl_2_, 10 mM Tris-HCl pH 8, plus 1 mM DTT, 0.40 U μL^-1^ RNase-In, 0.20 U μL^-1^ Superase-In and 0.1 % Triton X-100) and gently dissociated with 5 loose-pestle and 10 tight-pestle strokes, then adjusted to 1 mL with the same buffer. Lysis efficiency was confirmed by 1:1 Trypan Blue staining, after which lysates were passed through a 40 μm strainer into pre-cooled 15 mL tubes, centrifuged (600 g, 4 min, 4°C), washed once in PBS containing 0.2 U μL^-1^ RNase-In, and re-pelleted under the same conditions. Nuclei were resuspended in 200 μL PBS/RNase-In, re-filtered (40 μm), counted with a Countess 3 Automated Cell Counter (10 μL nuclei + 10 μL 0.4 % Trypan Blue; Thermo Fisher #T10282), and immediately fixed using the Evercode Nuclei Fixation v2 kit (Parse Biosciences, #ECF2103). Eight single-nucleus libraries were then prepared with the EVERCODE™ WT v2 kit (Parse Biosciences, #ECW02135) according to the manufacturer’s split-pool protocol. Library yield was quantified with a Qubit fluorometer and QuantiFluor dsDNA system (Promega #E2670), and fragment size distribution verified on an Agilent 2100 Bioanalyzer using the DNA 1000 kit (#5067-1504). Libraries were sequenced on an Illumina NovaSeq 6000 S4 flow-cell using paired-end 150-bp reads (PE150).

#### Single-cell and single-nucleus RNA-seq data processing and analysis

Raw paired-end FASTQ files from hESC single-cell (EVERCODE WT-Mini v2) and 3-month organoid single-nucleus (EVERCODE WT v2) libraries were inspected with FastQC (v0.11.9). Demultiplexing, read trimming, alignment to GRCh38/hg38 and transcript counting were performed with the Parse Biosciences command-line pipeline (commit 2024-02-20); the merge module combined the four sub-libraries per run to produce a gene–cell count matrix (.mtx), feature list and cell-level metadata.

Down-stream analyses were conducted in R (4.3.2) with Seurat (v5.0.1). Matrices were imported with ReadParseBio, and cells with >15,000 detected genes, >100,000 UMIs or >15 % mitochondrial UMIs were removed. Counts were log-normalised (NormalizeData, scale.factor = 10,000), 2,000 variable features were selected (FindVariableFeatures, method = “vst”), and expression values were centered and scaled (ScaleData). Dimensionality reduction used PCA (RunPCA); the first 30 principal components (PCs) were retained based on the elbow plot. A shared-nearest-neighbour graph was constructed (FindNeighbors, dims = 1:30) and Louvain clusters identified at resolution = 0.30 (FindClusters). Clusters were reordered by hierarchical tree building (BuildClusterTree) and visualised with UMAP (RunUMAP, dims = 1:30). Cluster markers were identified with FindAllMarkers (min.pct = 0.25, logfc.threshold = 0.25) and visualised by violin, dot, and feature plots. To assess large-scale genomic integrity in the hESC single-cell datasets, inferred copy-number variation (CNV) profiles were generated with inferCNV (R package, default parameters) using WT hESCs as the reference “normal” population. Genes were ordered by genomic position, expression values were smoothed across neighbouring genes, and denoised heatmaps were inspected for clonal gains or losses along chromosomes.

#### Whole-genome long-read sequencing (Nanopore)

Genomic DNA was extracted from hESC pellets using the DNeasy Blood & Tissue Kit (Qiagen) according to the manufacturer’s instructions. High-molecular-weight DNA (≈1–2 μg per sample) was used to prepare Oxford Nanopore sequencing libraries with the appropriate ligation-based library preparation kit, including native barcoding to allow multiplexing. Four barcoded samples were pooled and loaded on a single P2 flow cell (Oxford Nanopore Technologies) for sequencing. Basecalling was performed with the ONT Guppy pipeline using high-accuracy settings, and reads with low quality were discarded. Filtered reads were aligned to the GRCh38/hg38 reference genome with minimap2, and structural variants as well as large-scale copy-number changes were surveyed using standard long-read SV calling tools.

#### Spontaneous differentiation assay

hESC lines were seeded onto 2 wells of a 12-well plate previously coated with hESC-qualifed Matrigel (Corning, #354277). After a 72-h growth period with medium changes, the medium was removed for either Complete Cell Media w/15% FBS Serum and LIF (Sigma-Aldrich, #ES-101-B) or mTeSR Plus medium (Basal Medium #100-0274 supplemented 1:5 with mTeSR Plus 5× Supplement, #100-2075; STEMCELL Technologies). Then, the plate was transferred to an Incucyte S3 live-cell imager with culture condition as described previously. Nine-overlapping phase-contrast images per well were capture every 30 minutes for 6 h.

#### Western blot

Total proteins were extracted from WT, R133C, R168X and R270X hES cells at 0 day (ESC), 3 days (NE), 7 days (NSC) and 21 days (NPC) post induction using a RIPA Lysis Buffer, 10X (Sigma-Aldrich, #20-188) containing protease inhibitor cocktail (Sigma #S8820). Denatured proteins (10 μg for NESTIN and 20 μg for MAP2 and POU5F1) were separated by 3-8% Tris-Acetate or 4-12% Bis-Tris gel electrophoresis followed by transfer to nitrocellulose membranes. The membranes were blocked in 5% skim milk for one hour at room temperature, followed by incubation with an anti-NESTIN (CST #73349; 1/1,000), anti-MAP2 antibody (CST #8707; 1/1,000), or anti-POU5F1 antibody (Bio Legend 653702; 1/500) overnight at 4°C. The membranes were then incubated with the secondary antibody, donkey anti-rabbit (ab205722 at 1/10,000) or donkey anti-mouse (ab205724 at 1/10,000) for one hour at room temperature. The band intensities were quantified by densitometry using Image Lab 5.1 software (Bio-Rad). Protein levels were expressed as a ratio of protein-specific band density to GAPDH protein level (Abcam #ab181602 at 1/200,000).

#### Cell cycle analysis

The cell cyle of each hESC cell lines was performed using the Cell Cycle Analysis Kit (Abcam, ab287852). Briefly, the hESC cells were harvest using ReLeSR (Stem Cell Technlologies, #100-0483) and centrifuge at 400 x g for 5 minutes. The cell pellet was washed in cold 1X Cell Cycle Assay Buffer, then centrifuge again at 400 x g for 5 min. The cells were fixed using 70% Ethanol and incubate for 30 min at 4°C, then centrifuge at 400 x g for 5 min. The cell pellet was washed in cold 1X Cell Cycle Assay Buffer and centrifuge at 400 x g for 5 min. The pellets were resuspended in the Staining solution and incubate for 30min. The cell cycle stage was analysed with a BD FACSAria™ Fusion Cell Sorter (Plateforme cytométrie at Sainte-Justine hospital).

### Quantification and statistical analysis

All statistical procedures, n values, definitions of center, dispersion metrics, and exact *P* or adjusted *P* values are reported in the corresponding figure legends, main text, or Method subsections. A consolidated summary of analytical strategies is provided below.

**Table:** 

Experiment	Software	Statistical test or model	Definition of *n*	Measures of center ± dispersion	Multiple-test correction	Location
RT-qPCR (Figures 3D and 4B)	Prism 10 (GraphPad)	One-way ANOVA with Dunnett post-hoc (mutant vs WT)	Independent iPSC or hESC cultures	Mean ± s.e.m.	—	Figures 3D and 4B legend
Cell-growth (Figure 3H)	Incucyte S3 software + Prism 10	Two-way repeated-measures ANOVA	Colonies tracked over time (9 fields × 3 wells × 3 experiments per genotype)	Mean ± s.e.m.	—	Figure 3H legend
Cell-cycle phase counts (Figure 3G)	Seurat 5.0.1	Pearson χ^2^ goodness-of-fit vs WT	Single cells passing QC (exact cell counts in legend)	Proportion (% of cells)	—	Figures 3F and 3G legend
Bulk rna-seq DE (Figures 1 and 4)	DESeq2 1.40.2	Wald test (mutant vs WT)	Independent differentiations (3 per genotype & stage)	log_2_ fold-change	Benjamini–Hochberg padj < 0.01 &	log_2_FC
GO enrichment (Figures 1C and 2C)	clusterProfiler 4.8.3	Hypergeometric test	Gene universe = all expressed genes	GeneRatio, –log_10_ padj	Benjamini–Hochberg padj < 0.05	Methods → “Bulk RNA-seq analysis”
Machine learning (Figure 2)	R 4.3.2: stats::kmeans, randomForest 4.7-1.1, keras 2.13/TensorFlow 2.14	—	See below	OOB error, prediction probability	5-fold CV for RF; 25 % validation split for NN	Figure 2 legends

#### Randomization, blinding, and sample-size considerations

hESC and iPSC lines were plated and differentiated in parallel under identical conditions; wells were assigned randomly to imaging positions in Incucyte assays, and image acquisition/analysis were automated. Organoid batches were generated from separate vials per genotype to preserve biological replication. Investigators were not blinded to genotype during cell culture but downstream bioinformatic pipelines were scripted and executed without manual intervention. For exploratory *in vitro* systems we did not perform *a priori* power calculations; instead, sample sizes (three independent differentiations per condition, ≥2 900 single cells per scRNA-seq sample, three organoids per genotype for snRNA-seq) reflect field standards and our prior experience achieving reproducible effect sizes.

#### Inclusion and exclusion criteria

For RT-qPCR and growth assays all biological replicates were included. sc/snRNA-seq data were filtered to retain nuclei with <15 % mitochondrial RNA and 500 ≤ nFeature_RNA ≤ 15 000; doublets detected by index hopping were excluded. Bulk RNA-seq genes with ≤1 read across all samples were removed prior to DESeq2.

#### Definition of significance

Unless otherwise specified, P < 0.05 (after correction where applicable) was considered significant. Exact P, padj, or FDR values are provided in figure panels or legends.

## Supplemental information

Document S1. Figures S1–S7

Document S2. Article plus supplemental information
