# Supplementary material for: MECP2 mutations rewire human ESC fate and bias cortical lineage commitment
Source: Stem Cell Reports. 2026 Apr 23;21(5):102895. doi: 10.1016/j.stemcr.2026.102895 (PMC13163216; doi:10.1016/j.stemcr.2026.102895)
Supplement: Document S1. Figures S1–S7 [file mmc1.pdf]

Stem Cell Reports, Volume *21*

Supplemental Information

MECP2 mutations rewire human ESC fate and bias cortical lineage commitment

Marion Guillon, Margaux Brin, Elodie Gabet, Justine Gromaire, Mathéa Bernard, Laetitia Laurent, Théo Rabin, Lisa Bianchin, Marie Veziano, Julie Kloda, Alexia Bernard, Laila Asali, Yi Liu, and Anthony Flamier

SUPPLEMENTAL INFORMATION

Document S1. Supplemental Figures S1–S7.

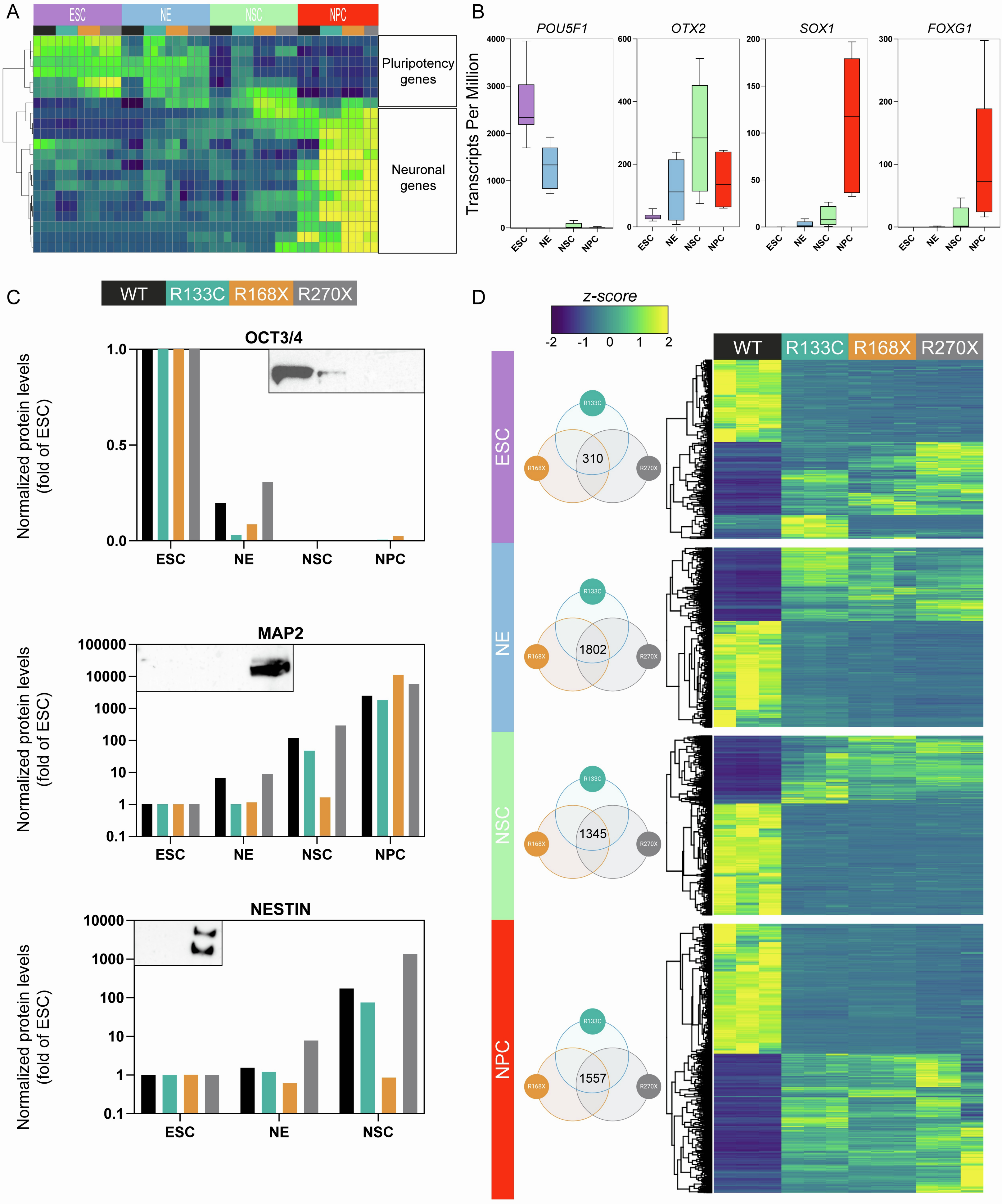
A
ESC
NE
NSC
NPC
Pluripotency genes
Neuronal genes
B
POU5F1
OTX2
SOX1
FOXG1
Transcripts Per Million
C
WT
R133C
R168X
R270X
OCT3/4
MAP2
NESTIN
Normalized protein levels (fold of ESC)
D
z-score
-2
-1
0
1
2
310
1802
1345
1557

Figure S1

Figure S1.

A. Z-score heat-map of curated pluripotency (top block) and neuronal (bottom block) marker genes across the four differentiation stages (ESC, NE, NSC, NPC; color bar above) for WT and the three mutant hESC lines (key below). Replicate columns are ordered by hierarchical clustering (Ward's method, Euclidean distance).

B. Average expression (TPM value) for four stage specific markers, extracted from bulk RNA-seq data.

C. Average protein abundance by Western-blot for three stage specific markers for each stage of differentiation.

D. For each stage, Venn diagrams (left) enumerate significantly dysregulated genes common to all mutants (Wald test, DESeq2; $\log_2 FC > 2$, $padj < 0.01$). The corresponding union set is visualized as a heat-map (right; same scaling as in A) with rows clustered by Pearson correlation.

A

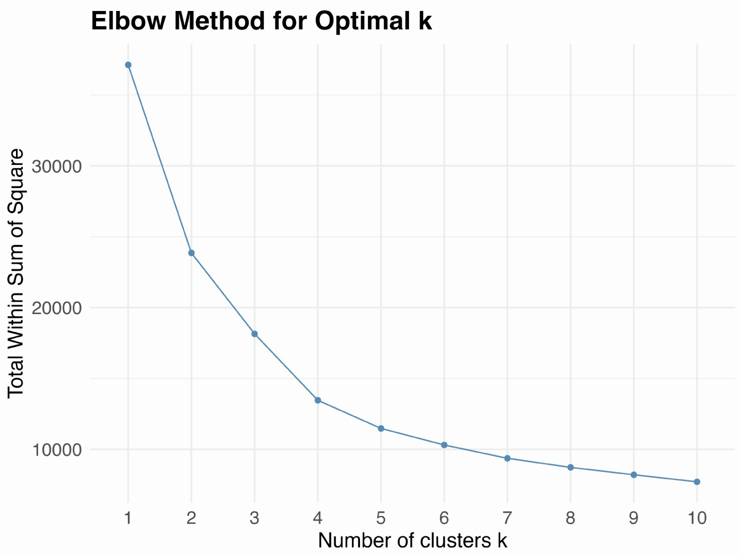

Elbow Method for Optimal k
Total Within Sum of Square
30000
20000
10000
1
2
3
4
5
6
7
8
9
10
Number of clusters k

B

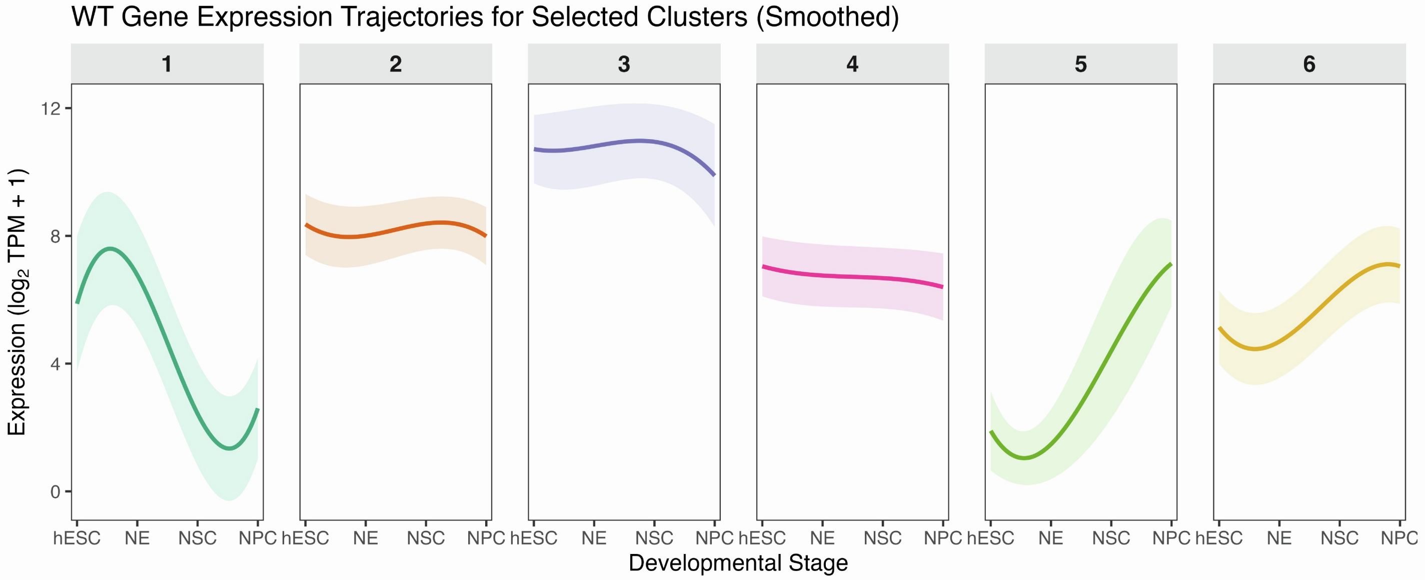

WT Gene Expression Trajectories for Selected Clusters (Smoothed)
1
2
3
4
5
6
Expression (log_2 TPM + 1)
12
8
4
0
hESC
NE
NSC
NPC
Developmental Stage

Figure S2

Figure S2.
A. Elbow plot of total within-cluster sum of squares across k = 1–10; k = 6 was chosen as the inflection point for all further k-means analyses.
B. Smoothed (loess) expression trajectories of WT genes belonging to the six selected clusters (shaded 95 % confidence band; y-axis, $\log_2$ TPM + 1; x-axis, developmental stage).

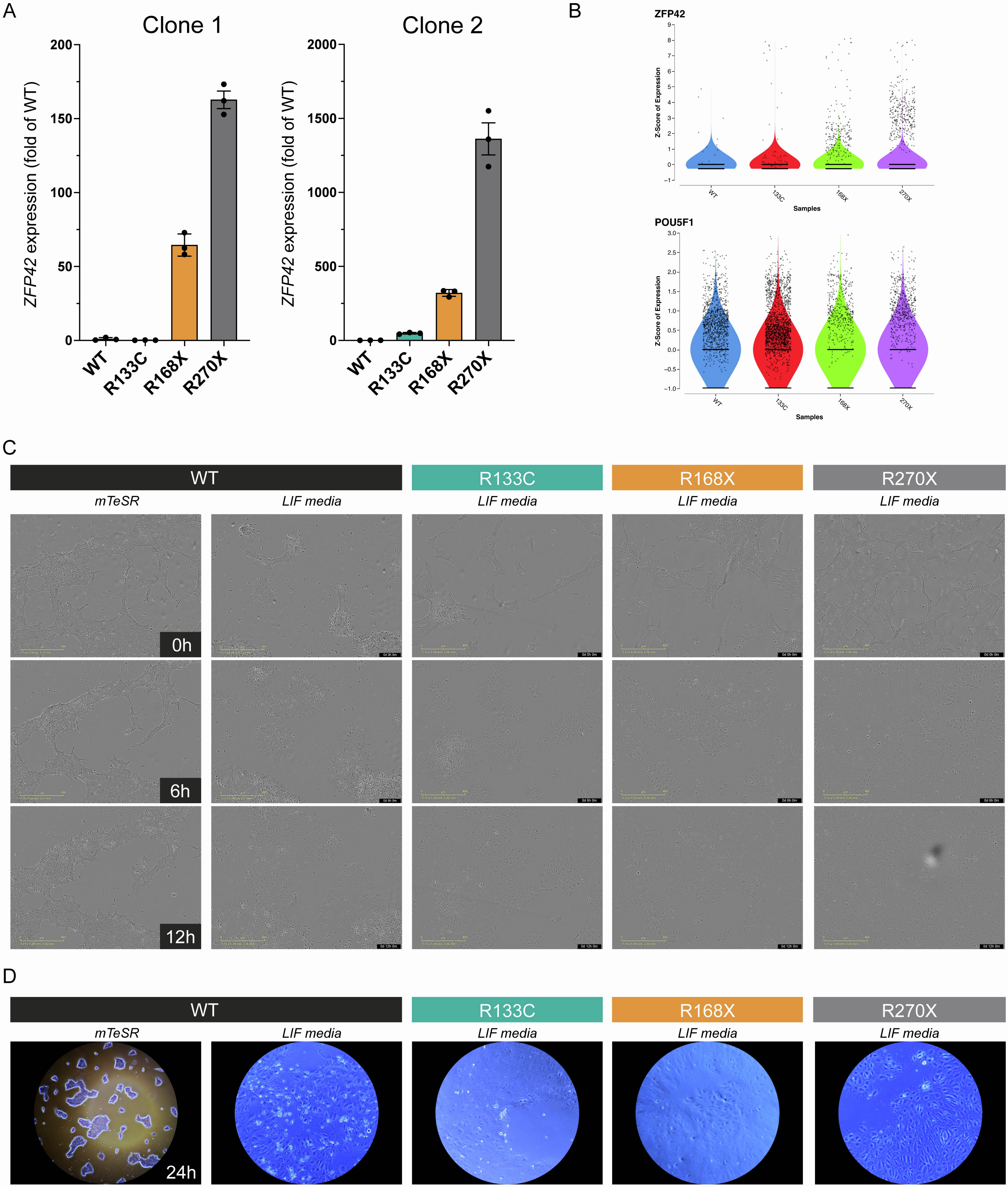
A
Clone 1
Clone 2
ZFP42 expression (fold of WT)
0
50
100
150
200
500
1000
1500
2000
WT
R133C
R168X
R270X
B
ZFP42
POU5F1
Z-Score of Expression
Samples
133C
168X
270X
C
mTeSR
LIF media
0h
6h
12h
D
24h

Figure S3

Figure S3.
A. Quantitative RT–PCR validation of *ZFP42/REX1* expression in two independent CRISPR-edited hESC clone series (Clone 1 and Clone 2) expressing *MECP2-WT, MECP2-R133C, MECP2-R168X* or *MECP2-R270X*. Expression is shown as fold change relative to the corresponding WT line (set to 1). Bars represent mean ± s.e.m.
B. Violin plots showing single-cell expression distributions of *ZFP42* and *POU5F1* across WT, R133C, R168X and R270X hESCs from scRNA-seq; each dot represents one cell.
C. Phase-contrast images of WT hESCs maintained in mTeSR and MECP2-mutant hESCs transferred to LIF-based medium, taken 0, 6 and 12 h after medium switch, illustrating loss of compact pluripotent colony morphology under LIF conditions.
D. hESC colonies after 24 h in mTeSR (WT) or LIF-based medium (WT and MECP2-mutant lines), showing failure of both WT and mutant hESCs to sustain naïve-like self-renewal in LIF conditions.

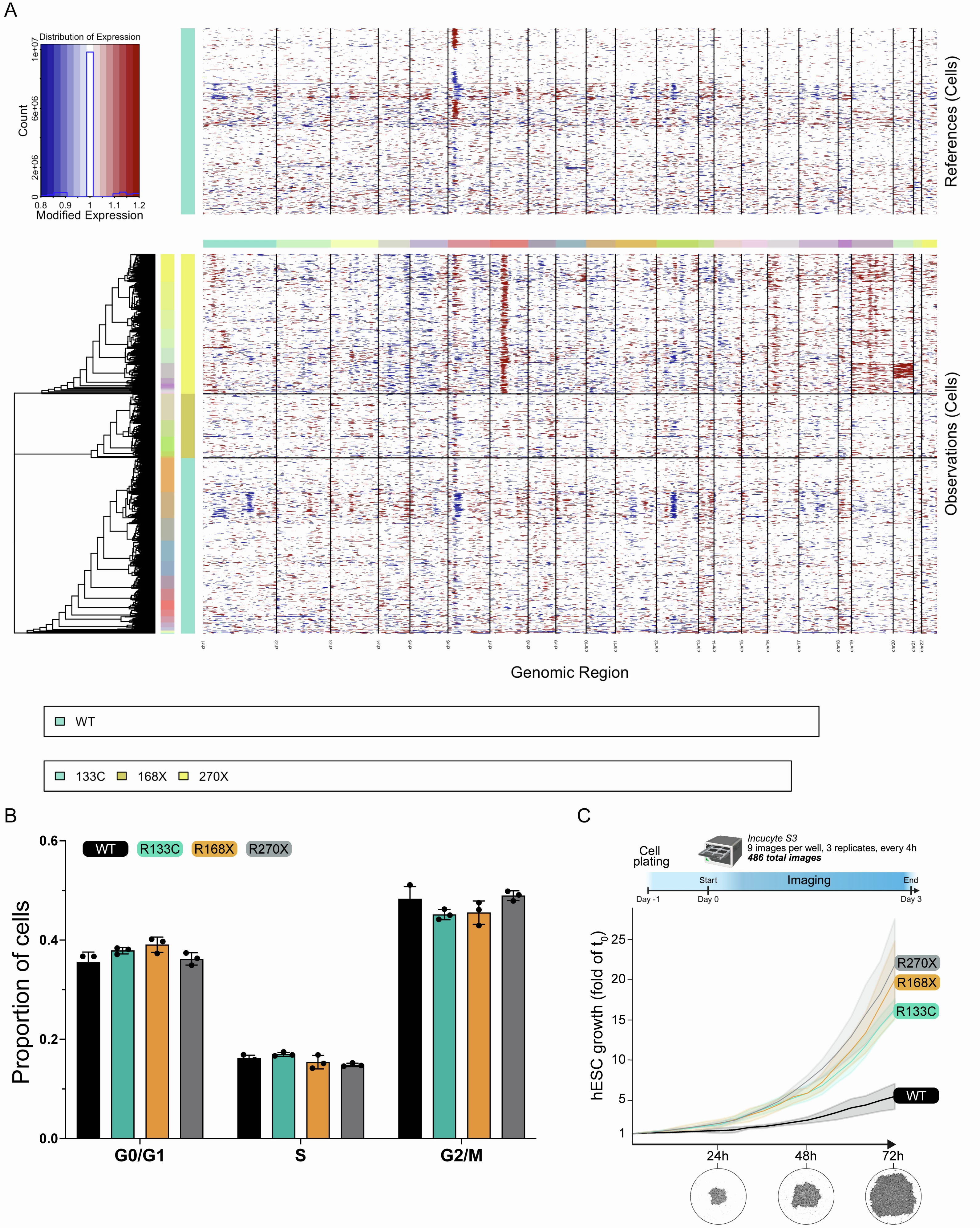

A
Distribution of Expression
Count
Modified Expression
References (Cells)
Observations (Cells)
Genomic Region
WT
133C
168X
270X
B
Proportion of cells
WT
R133C
R168X
R270X
G0/G1
S
G2/M
C
Cell plating
Incucyte S3
9 images per well, 3 replicates, every 4h
486 total images
Start
Imaging
End
Day -1
Day 0
Day 3
hESC growth (fold of t0)
R270X
R168X
R133C
WT
24h
48h
72h

Figure S4

Figure S4.
A. Top panel, reference WT cells; bottom panel, combined R133C, R168X and R270X observations. Each column represents a genomic region ordered chromosomally from 1 to Y (black tick marks) and each row a single cell, clustered by Euclidean distance. Red denotes inferred gains, blue denotes losses (scale bar, left). Color strips mark genotype identity of observation cells. Dendrogram shows hierarchical relationships among mutant cells.
B. Cell-cycle distribution of WT, R133C, R168X and R270X hESCs determined by flow cytometry. Cells were fixed, DNA content was measured by FACS, and the proportions of cells in G0/G1, S and G2/M phases were quantified. Bars represent mean ± s.e.m. of independent experiments.
C. Live-cell imaging proliferation assay, growth curves of hESC colonies over 72 h recorded on an Incucyte S3 (9 images/well, N=3); inset photographs illustrate colony morphology at 24, 48 and 72 h.

A

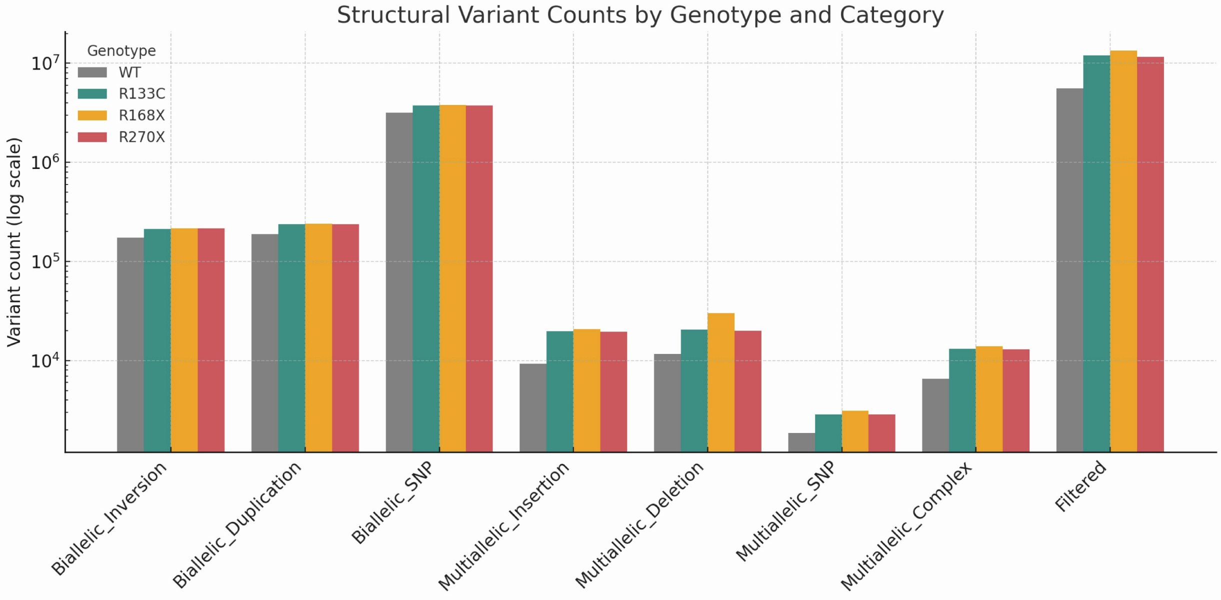
Structural Variant Counts by Genotype and Category
Genotype
WT
R133C
R168X
R270X
Variant count (log scale)
10^7
10^6
10^5
10^4
Biallelic_Inversion
Biallelic_Duplication
Biallelic_SNP
Multiallelic_Insertion
Multiallelic_Deletion
Multiallelic_SNP
Multiallelic_Complex
Filtered

B

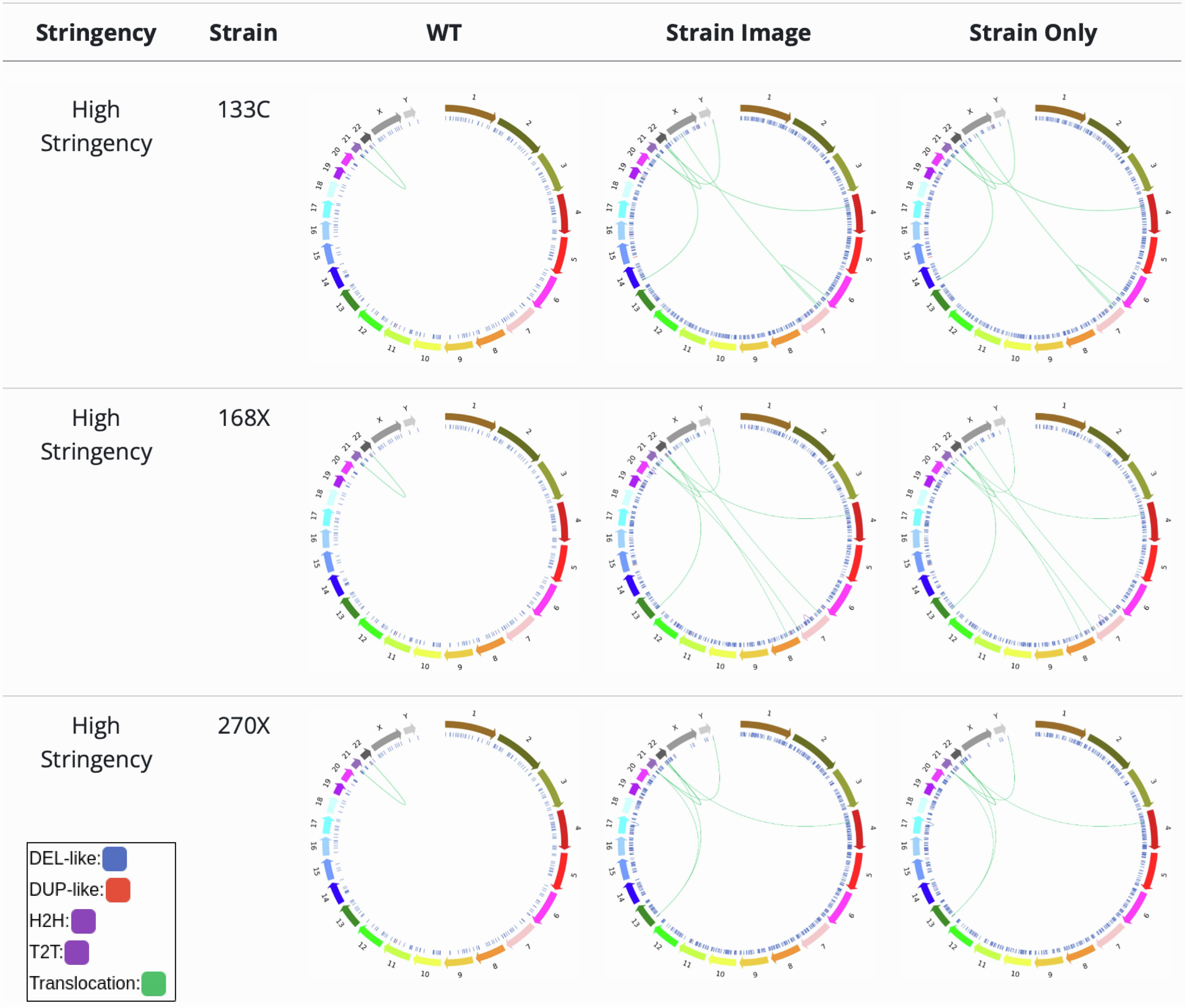
Stringency
Strain
WT
Strain Image
Strain Only
High Stringency
133C
High Stringency
168X
High Stringency
270X
DEL-like:
DUP-like:
H2H:
T2T:
Translocation:

Figure S5

Figure S5.
A. Grouped bar chart of structural-variant (SV) counts binned by type ($\log_{10}$ scale) for WT and mutants at high-stringency Sniffles2 settings (≥8 supporting reads, ≥150 bp).
B. Circos plots of SVs retained after comparison with the WT call-set ("Strain Only") for each mutant. Outer track, chromosomes 1–22, X, Y; inner chords, high-confidence SVs colored by class (legend, bottom left). Central column shows WT reference, confirming removal of shared variants.

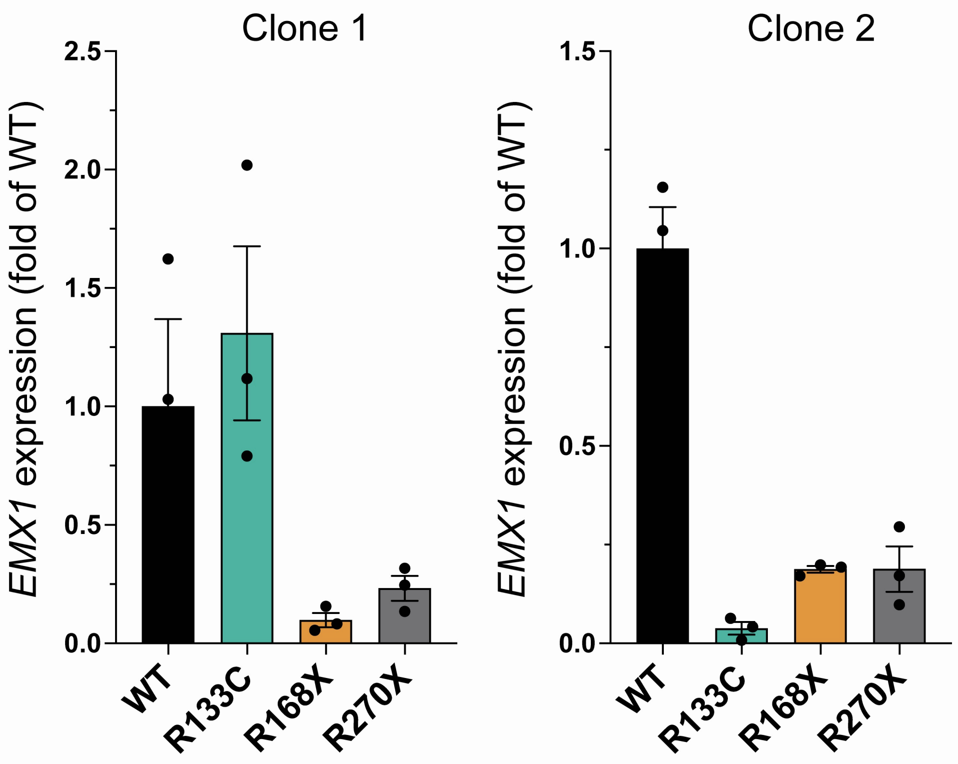

Clone 1
Clone 2
EMX1 expression (fold of WT)
2.5
2.0
1.5
1.0
0.5
0.0
WT
R133C
R168X
R270X

Figure S6

Figure S6.

Quantitative RT–PCR validation of *EMX1* expression in two independent CRISPR-edited hESC clone series (Clone 1 and Clone 2) expressing *MECP2-WT, MECP2-R133C, MECP2-R168X* or *MECP2-R270X* at the ESC stage. Expression is shown as fold change relative to the corresponding WT line (set to 1). Bars represent mean ± s.e.m.

A

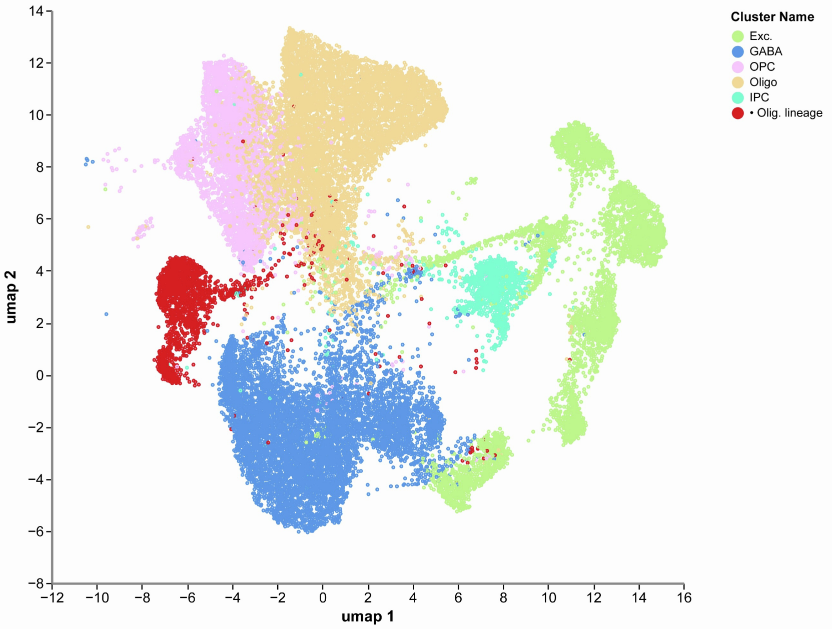
Cluster Name
Exc.
GABA
OPC
Oligo
IPC
• Olig. lineage
umap 1
umap 2

B

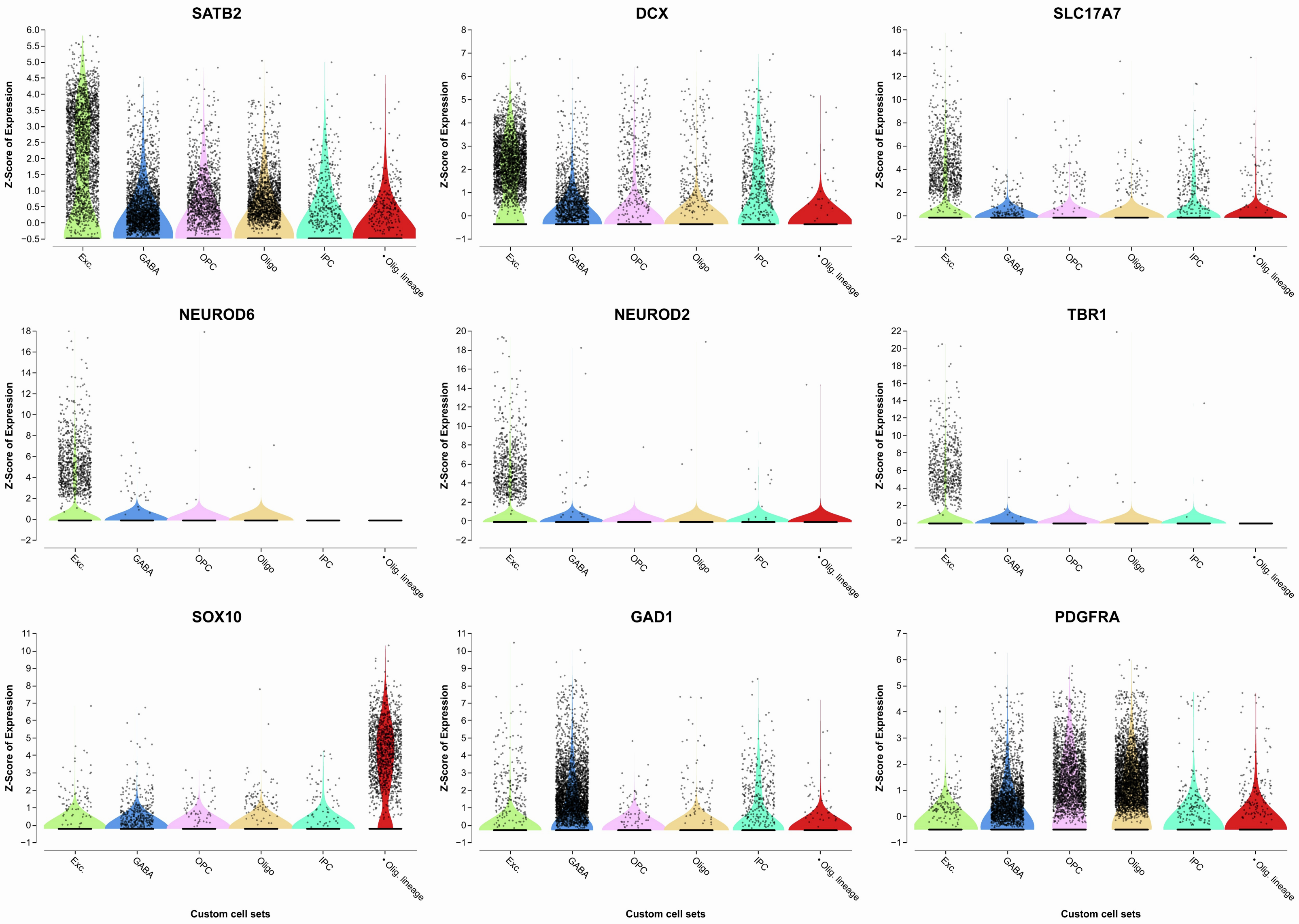
SATB2
DCX
SLC17A7
NEUROD6
NEUROD2
TBR1
SOX10
GAD1
PDGFRA
Z-Score of Expression
Exc.
GABA
OPC
Oligo
IPC
• Olig. lineage
Custom cell sets

Figure S7

Figure S7.

A. UMAP visualization of single-nucleus RNA-seq profiles from 3-month unguided cerebral organoids (all genotypes combined). Cells are colored according to the final cell-type categories used in Figure 4 (glutamatergic/excitatory neuron lineage, inhibitory/GABAergic neurons, oligodendrocyte progenitor cells, oligodendrocyte lineage and broader glial/precursor populations).

B. Violin plots showing expression of canonical marker genes used to assign these identities: *SATB2*, *DCX* and *SLC17A7* for glutamatergic neuron lineage, *NEUROD6*, *NEUROD2* and *TBR1* for cortical excitatory projection neurons, *SOX10* and *PDGFRA* for oligodendrocyte/OPC lineages, and *GAD1* for inhibitory/GABAergic neurons. Each dot represents a single nucleus.
