# Supplementary material for: MECP2 mutations rewire human ESC fate and bias cortical lineage commitment
Source: Stem Cell Reports. 2026 Apr 23;21(5):102895. doi: 10.1016/j.stemcr.2026.102895 (PMC13163216; doi:10.1016/j.stemcr.2026.102895)
Supplement: Document S2. Article plus supplemental information [file mmc2.pdf]

STEM CELL REPORTS
Article

OPEN ACCESS

# *MECP2* mutations rewire human ESC fate and bias cortical lineage commitment

Marion Guillon,[1,5] Margaux Brin,[1,5] Elodie Gabet,[1] Justine Gromaire,[1] Mathéa Bernard,[1] Laetitia Laurent,[1] Théo Rabin,[1] Lisa Bianchin,[1] Marie Veziano,[1] Julie Kloda,[1] Alexia Bernard,[1] Laila Asali,[1] Yi Liu,[2,3] and Anthony Flamier[1,4,6,*]

[1]Centre de recherche Azrieli du CHU Sainte-Justine, Montreal, QC, Canada
[2]Regenerative Medicine Program, Ottawa Hospital Research Institute, Ottawa, ON, Canada
[3]Department of Cellular and Molecular Medicine, Faculty of Medicine, University of Ottawa, Ottawa, ON, Canada
[4]Department of Neurosciences, Université de Montréal, Montreal, QC, Canada
[5]These authors contributed equally
[6]Lead contact
*Correspondence: anthony.flamier@umontreal.ca
https://doi.org/10.1016/j.stemcr.2026.102895

## SUMMARY

Rett syndrome arises from loss-of-function mutations in the X-linked chromatin regulator MECP2, yet the earliest molecular derailments in development are poorly defined. Using isogenic human embryonic stem cell (hESC) models carrying three patient-derived *MECP2* mutations, we followed the transcriptome from pluripotency through neuroectoderm, neural stem/progenitor stages. Developmental stage dominated transcriptional variance, but mutants shared a secondary program enriched for synaptic-membrane and extracellular matrix genes. Single-cell/bulk profiling at the embryonic stem cell (ESC) stage revealed partial naïve-like drift, marked by the up-regulation of the naïve-enriched factor *ZFP42*/REX1 and related markers in *MECP2*-mutant lines. Among convergently dysregulated genes, the cortical determinant *EMX1* showed an abnormal developmental trajectory, early repression followed by overshoot, and was consistently altered across independent Rett PSC models. Single-nucleus RNA-seq of cerebral organoids uncovered allele-specific yet convergent disturbances in cortical lineage allocation. These data chart a continuous developmental trajectory for *MECP2*-mutant cells and nominate naïve-like drift and mis-timed *EMX1* expression as tractable entry points for dissecting Rett pathogenesis.

## INTRODUCTION

Rett syndrome (RTT) is a severe X-linked neurodevelopmental disorder that affects ≈1 in 10,000 girls worldwide and, in rare surviving males, presents with an even more severe encephalopathy (Liu et al., 2025b; Petriti et al., 2023). After a seemingly normal first 6–18 months, infants enter a protracted period of neuro-regression marked by loss of purposeful hand use and speech, seizures, gait ataxia, autistic features, and severe intellectual disability (Guy et al., 2001; Liao, 2019; Liu et al., 2025a, 2025b; Moog et al., 2003; Neul and Zoghbi, 2004; Petriti et al., 2023; Raspa et al., 2025). Structural MRI and postmortem studies consistently show reduced total brain volume, cortical thinning, simplified dendritic arbors, and a shift in excitatory-inhibitory balance, suggesting that pathogenic processes likely begin during early corticogenesis and extend into postnatal synaptic maturation (Armstrong et al., 1995; Chao et al., 2007; Dani et al., 2005; Ip et al., 2018; Reiss et al., 1993).

More than 95% of classic RTT cases arise from *de novo* loss-of-function mutations in *MECP2*, which encodes methyl-CpG-binding protein 2 (MECP2). (Amir et al., 1999) Historically, MECP2 was viewed as a canonical methyl-DNA reader that blankets the neuronal genome and recruits co-repressor complexes to silence transcription (Nan et al., 1998). Recent molecular studies on adult mouse cortex and human embryonic stem cell (ESC)-derived neurons refine this view: MECP2 binds preferentially to discrete enhancer-like elements, termed MECP2-binding hotspots (MBHs), and can do so even in regions with relatively low CpG methylation (Bajikar et al., 2025; Y. Li et al., 2013; Liu et al., 2024; Liu et al., 2025b; Mishra et al., 2025). Clusters of MBHs cooperate to the dampen transcription of genes enriched for neuronal functions, revealing an intragenic, partly methylation-independent mode of repression that likely complements the classical methylation-dependent mechanism (Liu et al., 2024; Mishra et al., 2025). We and others also found that MECP2 binds both unmethylated and methylated cytosines to act as both a transcriptional repressor (through its NCoR domain) and activator (through RNA polymerase II recruitment) in neurons (Chahrour et al., 2008; Li et al., 2013; Liu et al., 2024; Lyst et al., 2013; Sharifi et al., 2024). However, the mechanistic context of these findings remains unclear in early development. Mammalian ESCs and blastocyst-stage embryos are globally hypomethylated, a state long thought to preclude meaningful MECP2 engagement and thus to spare pluripotent cells from RTT-associated lesions (Guo et al., 2014). The discovery that MECP2 can also bind hypomethylated regions to regulate transcription now raises the possibility that


Published by Elsevier Inc. on behalf of International Society for Stem Cell Research.
This is an open access article under the CC BY license (http://creativecommons.org/licenses/by/4.0/).

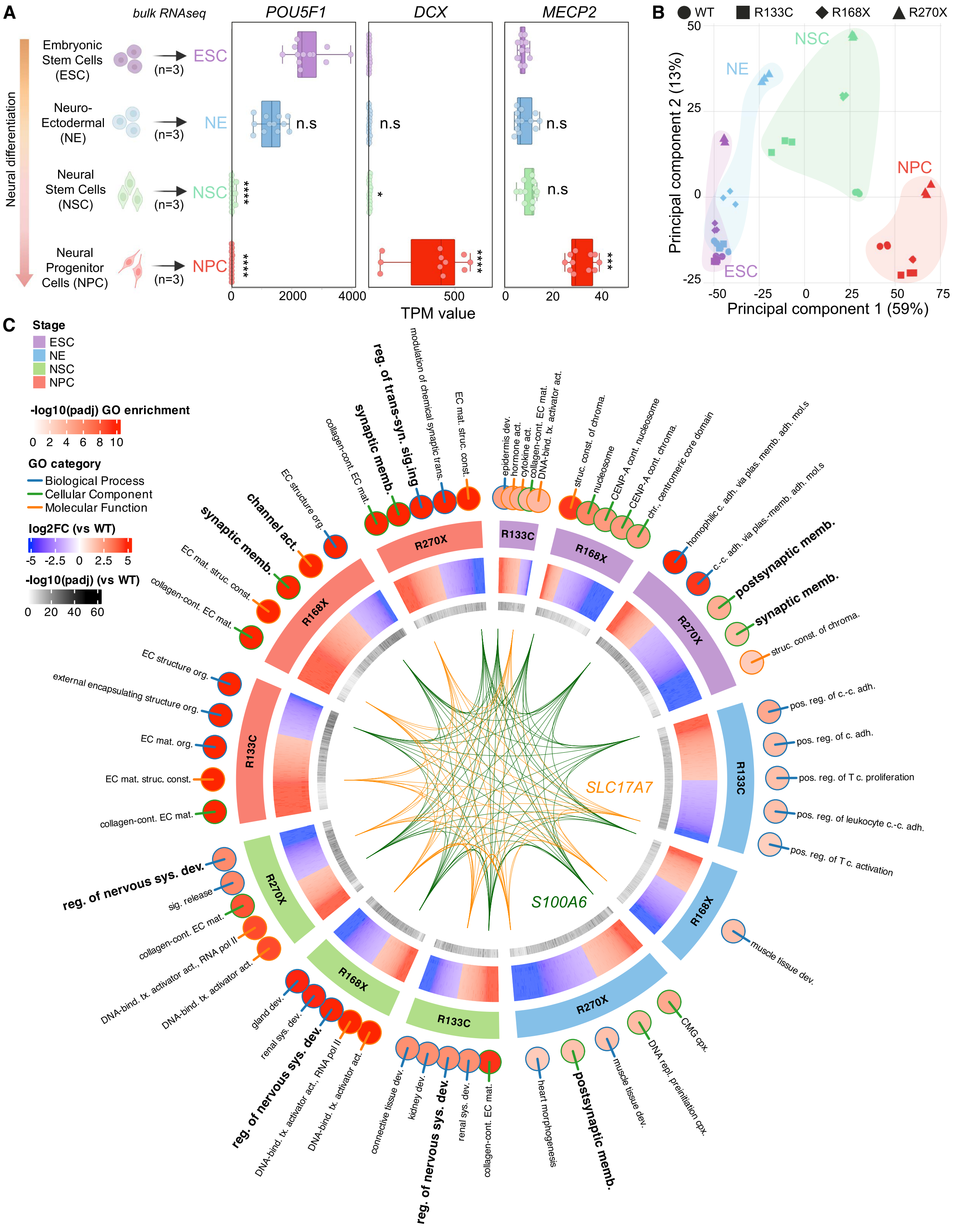


(legend on next page)

MECP2 deficiency could perturb transcriptional programs as early as the hypomethylated ESC stage, well before neurons are specified (Liu et al., 2024, 2025b). Determining whether such early dysregulation contributes to later cortical deficits is therefore a central unanswered question that we address in this study.

To interrogate this possibility in a continuous human model, we used isogenic human male hESC lines harboring three recurrent RTT mutations (R133C, R168X, and R270X) (Neul et al., 2008) and performed longitudinal profiling across four matched developmental milestones—pluripotent ESC, neuro-ectoderm (NE), neural stem cell (NSC) and neural progenitor cell (NPC)—and in long-term cerebral organoids. We further used RTT patient-derived induced pluripotent stem cell (iPSC) lines to validate our observations. By integrating bulk and single-cell RNA sequencing with gene-set enrichment and machine-learning classifiers, we uncovered a stage-persistent, MECP2-dependent transcriptional program that elevates naïve-enriched stem cell markers and increases ESC proliferation without full conversion to naive pluripotency, enhances synaptic-membrane pathways during neural induction, and imposes an abnormal developmental trajectory of the cortical radial-glia determinant EMX1. In three-month cerebral organoids, these early changes are accompanied by allele-specific but convergent alterations in cortical lineage allocation, with shifted proportions of glutamatergic neuron lineage, inhibitory neurons, and glial populations. These data reveal discrete molecular lesions that precede overt neuronal dysfunction and provide new developmental entry points for mechanistic dissection in RTT.

## RESULTS

### Stage-resolved transcriptomics reveals early, genotype-specific disruptions in MECP2-mutant differentiation

To test whether MECP2 loss perturbs human neurodevelopment from its earliest hypomethylated state, we profiled three previously described CRISPR-edited, isogenic hESC lines carrying common RTT mutations (R133C, R168X, and R270X) alongside WT controls as they were sequentially coaxed through ESC (day 0), NE (day 3), NSC (day 7) and NPC (day 21) stages (Figure 1A).(Liu et al., 2024) As expected, the pluripotency factors plunged after the ESC stage, whereas neuronal markers became detectable at NSC and rose thereafter, confirming efficient lineage induction (Figures 1A and S1A–S1C). *MECP2* itself remained low but readily measurable at ESCs (5–10 TPM), corroborating published observations (Figure 1A) (Li et al., 2013). *MECP2* increased modestly at NE/NSC and surged at NPC (Figure 1A). This suggests that residual embryonic expression could already influence transcriptional programs. Principal-component analysis showed that PC1 (59% variance) stratified samples by developmental stage, validating the differentiation axis, whereas PC2 (13%) captured mutation-specific variance that emerged progressively: R270X diverged from WT at the ESC stage, R168X detached by NE, and all mutants formed distinct genotype clusters by NSC/NPC (Figure 1B). GO enrichment revealed significant dysregulation beginning at ESCs, most strikingly in R270X, where synaptic-membrane genes were already mis-expressed (Figure 1C). NSC samples displayed enrichment for the "regulation of nervous-system development," and NPCs showed broad perturbation of synaptic and ion-channel terms (Figure 1C). We observed common gene dysregulations across genotypes and stages (Figures 1C and S1D). Two transcripts were consistently altered in every genotype at every stage: *S100A6*, a calcium-binding protein implicated in cytoskeletal dynamics and stem-cell proliferation, and *SLC17A7*/*VGLUT1*, the principal vesicular glutamate transporter essential for excitatory neurotransmission (Figure 1C) (Cheret et al., 2021; Donato et al., 2017; Wang et al., 2023). Early dysregulation of *S100A6* suggests cytoskeletal or proliferative defects could precede neurogenesis, whereas persistent mis-expression of *SLC17A7* foreshadows later synaptic dysfunction (Cheret et al., 2021; Jurewicz et al., 2020; Li et al., 2017). Collectively, these findings suggest that *MECP2* mutations impose transcriptomic disturbances as early as the blastocyst-like ESC stage, raising the possibility that altered lineage trajectories, not only neuronal maturation defects, contribute to RTT pathogenesis.

**Figure 1.** ***Stage-resolved transcriptomics reveal early gene-network disruption in MECP2-mutant hESC differentiation***

(A) Expression dynamics of key developmental markers across the four matched stages analyzed on 4 RTT hES cell lines (WT, R133C, R168X et R270X). Box-and-scatter plots show transcripts per million (TPM; $n = 3$ biological replicates per stage and genotype) for the pluripotency factor *POU5F1*/OCT4, the early neuronal marker *DCX*, and *MECP2* itself. Tukey-adjusted post-hoc significance relative to the ESC stage (*$p < 0.05$, ***$p < 0.001$, and ****$p < 0.0001$).

(B) Principal component analysis of the complete bulk RNA-seq dataset.

(C) Circular multi-layer GO enrichment map integrating all three mutants. Outer colored dots show the top 5 GO terms significantly enriched at each stage (ESC, NE, NSC, NPC) and for each genotype versus WT (neural GO terms in bold); color scale encodes log2 fold-change versus WT (red, up-regulated; blue, down-regulated). Inner ring indicates −log10 padj of differential gene expression. The center chord diagram links the two transcripts (*S100A6*, green chords; *SLC17A7*, orange chords) that are consistently dysregulated in every genotype at every stage.

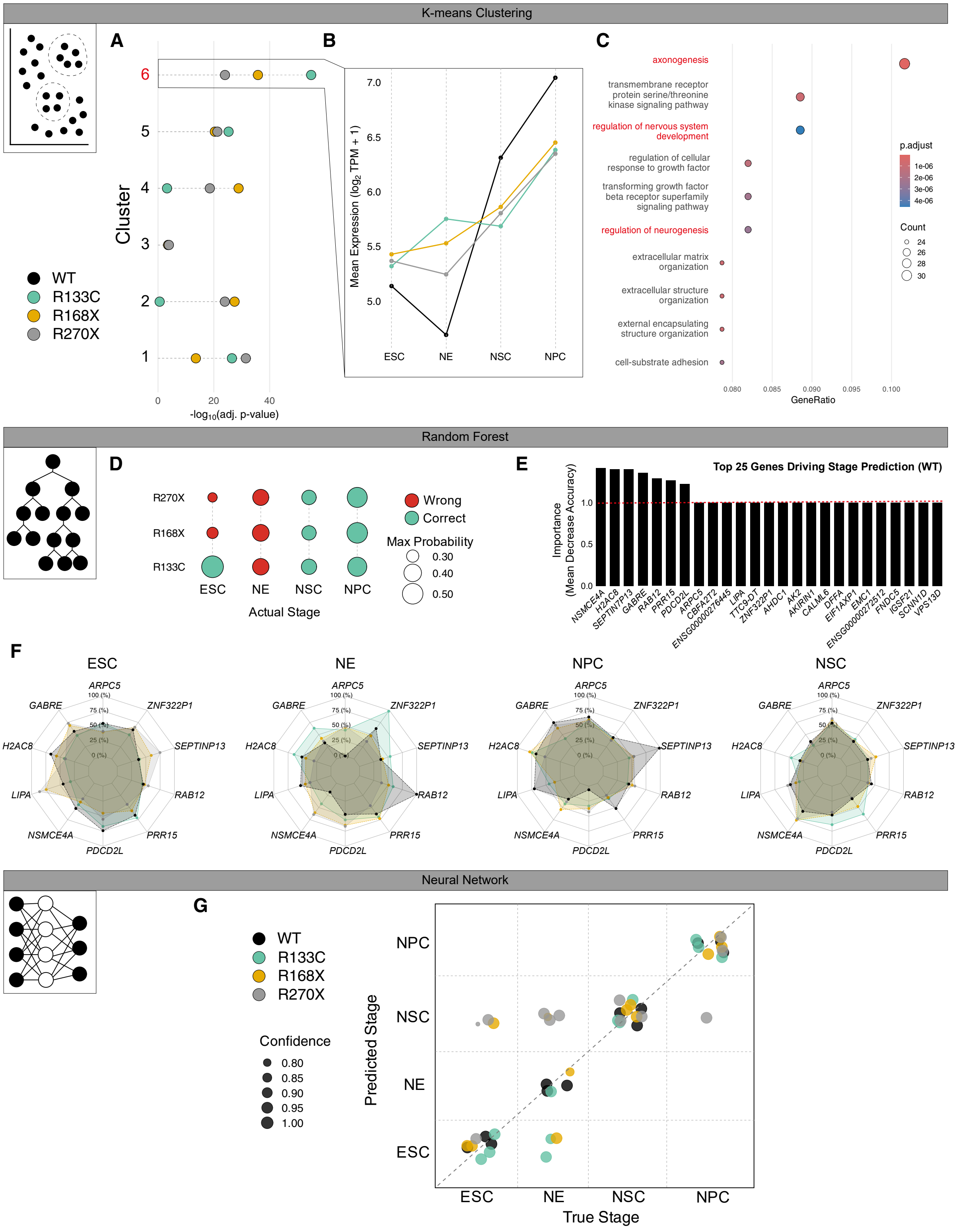


**Figure 2. Machine-learning classifiers expose premature neuronal program activation in MECP2 mutants**

(A) Adjusted *p*-values ($-\log_{10}$ scale) for the six gene clusters identified by K-means (left margin indicates cluster number).

(B) Mean expression profile ($\log_2$ TPM +1) of the genes represented in cluster 6 across the four sampled stages and the four genotypes. Genotypes are color-coded as in the inset legend.

*(legend continued on next page)*

## Machine-learning models accurately decode developmental stage and highlight early neuronal mis-timing in MECP2 mutants

We asked whether *MECP2* mutations create transcriptomic shifts large enough for detection by machine-learning and, if so, which genes drive those shifts. To this end, we analyzed our bulk RNA sequencing matrix with three complementary algorithms: Unsupervised k-means clustering served for pattern discovery; a supervised random-forest ensemble offered interpretable prediction; a feedforward neural network provided high-capacity validation. K-means partitioned genes into six clusters (Figures 2A, S2A, and S2B). Cluster 6 showed a steady increase in expression from the ESC/NE stage to the NSC and NPC stages (Figures 2B and S2B). Notably, this trajectory was the most significantly altered in the three mutant lines (Figures 2A and 2B). Gene-Ontology analysis linked this cluster to axonogenesis (e.g., *CACNA1F*, *CNTN2*, and *DLX5*) and regulation of nervous-system development, implying that neuronal programs are engaged unusually early in *MECP2*-deficient pluripotent cells (Figure 2C). The random forest, trained on labeled samples, achieved more than 95% cross-validated accuracy (Figure 2D). Feature-importance scores highlighted ten transcripts that dominate stage discrimination (Figure 2E). For example, *GABRE,* encoding the ε-subunit of the GABA-A receptor shaping inhibitory tone, was elevated in mutants at ESC and NE but normalized or fell below wild-type at NSC and NPC (Figure 2F) (Nguyen and Nicoll, 2018; Sigel and Steinmann, 2012). *PDCD2L*, with a similar profile, plays a role in neurodevelopment, particularly in programmed cell death (PCD) and ribosomal biogenesis (Figure 2F). (Li et al., 2023a) PCD is essential for stem cell maintenance and neurodevelopment, as it involves the elimination of a subset of neurons to refine neural circuits (Guo et al., 2024). Finally, *NSMCE4A*, the most important gene of this classifier, is part of the SMC5/6 complex, which is key for chromosome architecture and genomic stability during neurogenesis (Atkins et al., 2020; Horváth et al., 2020). The gene expression profile mirrors Cluster 6 and suggests premature activation followed by later exhaustion of inhibitory-synapse genes (Figure 2B). A feedforward neural network trained on the 2,000 most variable genes reproduced stage calls with all WT samples on the diagonal of true versus predicted stage (Figure 2G). This model suggests that the R270X line exhibited the most divergent alignment from the WT trajectory, particularly in the NE stage. Convergence across k-means, random forest, and neural network indicates that the developmental stage still dominates variance, yet *MECP2* mutations superimpose an early surge and later collapse of neuronal and synaptic transcripts. This temporal misalignment helps automated models flag mutant ESCs and underscores the need to dissect transcriptional disruption at the blastocyst-equivalent stage.

## MECP2 loss promotes a naïve-like transcriptional program and increased proliferation in human ESCs

To determine whether the early transcriptional disruption at the pluripotent stage arises from heterogeneous differentiation states, we performed single-cell RNA sequencing on undifferentiated hESCs carrying WT, R133C, R168X, or R270X MECP2 alleles. UMAP embedding revealed four genotype-specific clusters: WT and R133C cells co-localized, whereas R168X and R270X formed distinct groupings that recapitulate our bulk RNA-seq observations (Figure 3A); the absence of subclusters within each genotype supports cellular homogeneity (Figure 3A). The expression of the core pluripotency factor *POU5F1* was uniform, yet truncating mutants (R168X and R270X) exhibited striking upregulation of *ZFP42*/REX1 (Figure 3B). ZFP42 encodes a zinc-finger protein primarily expressed in undifferentiated stem cells (Masui et al., 2008; Shi et al., 2006) and is enriched in naive relative to primed pluripotency, while remaining detectable at lower levels in primed ESCs (Ghimire et al., 2018; Marks et al., 2012; Takahashi et al., 2017; Theunissen et al., 2016). Consistent with this, WT hESCs show low but measurable *ZFP42* expression, whereas R168X and R270X display a marked increase that is reproduced in two independent CRISPR-edited clones (Figures S3A and S3B). A survey of 32 canonical pluripotency genes revealed no significant dysregulation, excluding a broad shift in pluripotency identity (Figure 3C). InferCNV analysis of the single-cell transcriptomes detected no copy-number alterations, and long-read whole-genome sequencing confirmed that all lines retain the expected on-target genotype (Figures S4A and S5). Thus, elevated *ZFP42* in truncating mutants is best interpreted as a naïve-like transcriptional bias within an

(C) Dot plot of GO terms enriched in Cluster 6. Dot diameter corresponds to the number of genes annotated to the term; color encodes Benjamini-Hochberg adjusted $p$-value(D) Confusion matrix displays random-forest stage predictions for each genotype. Circle size denotes the classifier's maximum posterior probability; color indicates correct or incorrect assignment.

(E) Bar chart of the top 25 genes ranked by mean decrease in accuracy when permuted, obtained from the model trained on WT samples.

(F) Radar plots of the ten most informative transcripts (E) show their median expression at each stage for WT and the three *MECP2*-mutant lines.

(G) Scatterplot compares predicted versus true developmental stages for all samples. Point color denotes genotype; point size reflects the model's confidence (posterior probability). The dashed diagonal marks perfect agreement.

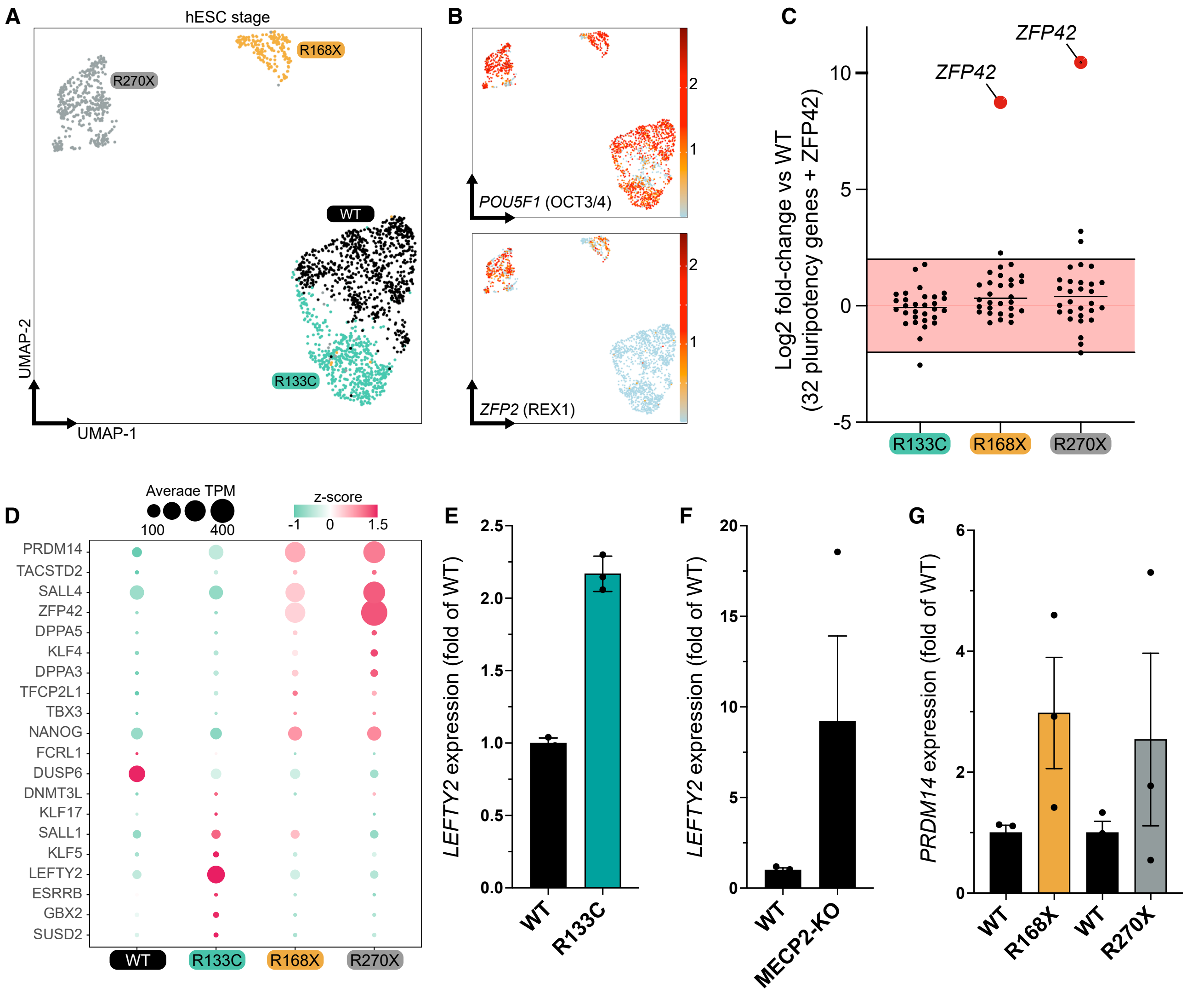


**Figure 3. MECP2 loss drives a naïve-like transcriptional shift and hyperproliferation in human ESCs**

(A) UMAP projection of 2,925 single hESC transcriptomes colored by genotype (WT, R133C, R168X, R270X) at the pluripotent stage.

(B) Feature plots show the per-cell log-normalized expression of the core pluripotency factor *POU5F1*/OCT4 (top) and the naïve-associated marker *ZFP42/REX1* (bottom) on the same UMAP embedding.

(C) Dot plot summarizes $\log_2$ fold-change (mutant vs. WT) for a panel of 32 canonical pluripotency genes (black dots); red dots highlight *ZFP42* values. Computed from the bulk RNA sequencing matrix.

(D) Dot plot displays average expression (dot size) and *Z* score (color scale) of the 20 naïve-enriched stem-cell markers across WT and mutant hESCs. Computed from the bulk RNA sequencing matrix.

(E) Quantitative RT-PCR validation of *LEFTY2* expression in patient-derived isogenic iPSC lines expressing either *MECP2-WT* or *MECP2-R133C* transcripts. Bars represent mean ± s.e.m.

(F) Quantitative RT-PCR validation of *LEFTY2* expression in a WT iPSC line and its isogenic line KO for *MECP2* alleles. Bars represent mean ± s.e.m.

(G) Quantitative RT-PCR validation of *LEFTY2* expression in patient-derived isogenic iPSC lines expressing either *MECP2-WT*, *MECP2-R168X,* or *MECP2-R270X* transcripts. Bars represent mean ± s.e.m.

otherwise intact pluripotent state, rather than as evidence of pluripotency loss or genomic instability.

We next asked whether this naïve-like signature extends beyond ZFP42. Interrogation of the top 20 naïve-enriched markers revealed that most are upregulated in *MECP2*-mutant hESCs, with *PRDM14* and *SALL4* particularly elevated in R168X and R270X, while R133C selectively increases a distinct subset of naïve-associated genes

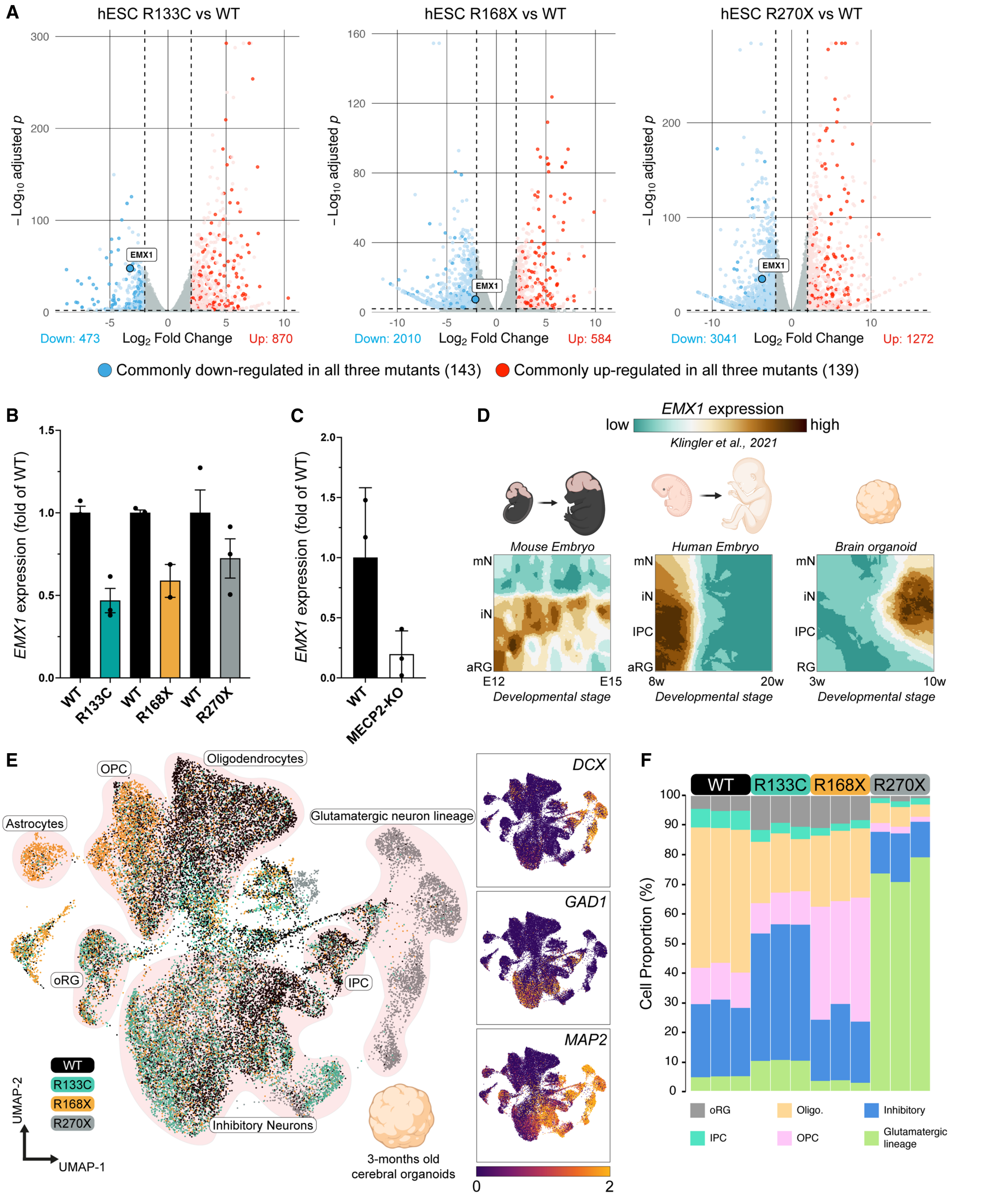


**Figure 4. Convergent EMX1 repression links early defects to reduced excitatory-neuron output in Rett organoids**

(A) Volcano plots of differential expression ($\log_2$ fold-change vs. $-\log_{10}$ adjusted p) for each mutant hESC line relative to WT. Genes down-regulated in all three mutants are highlighted in blue ($n$ = 143) and up-regulated genes in red ($n$ = 139). The cortical progenitor determinant *EMX1* is indicated in each plot. Computed from the bulk RNA sequencing matrix.

*(legend continued on next page)*

(Figure 3D). To test whether these changes generalize across pluripotent backgrounds, we examined naive markers in additional iPSC models. *LEFTY2*, a naïve-associated TGFβ ligand, was increased in R133C relative to its isogenic WT control and similarly elevated in an independent iPSC line in which *MECP2* was knocked out by CRISPR (Figures 3E and 3F). *PRDM14* was likewise upregulated in iPSCs carrying R168X or R270X relative to their respective isogenic controls (Figure 3G). Together, these data indicate that loss of *MECP2*, particularly truncating alleles, reproducibly induces a partial naïve-like transcriptional drift across distinct human pluripotent contexts.

To functionally probe naive identity, we transferred WT and mutant hESCs from standard mTeSR medium to a LIF-based, FGF/TGFβ-free medium that supports self-renewal of bona fide naive human PSCs. Under these conditions, neither WT nor mutant colonies maintained long-term self-renewal: Cells rapidly lost compact morphology and differentiated in all genotypes (Figures S3C and S3D). This confirms that *MECP2*-mutant hESCs do not fulfill functional criteria for naive pluripotency. Finally, live-cell imaging of colony expansion revealed that all three *MECP2*-mutant lines proliferate faster than WT over 72 h, whereas DNA-content flow cytometry showed broadly similar G0/G1, S, and G2/M fractions across genotypes (Figures S4B and S4C). Thus, M*ECP2* loss in hESCs is associated with a modest increase in proliferative capacity and a biased, but incomplete, acquisition of naïve-like transcriptional features, highlighting ZFP42 and related markers as focal nodes for mechanistic investigation.

## Convergent EMX1 repression in MECP2-mutant stem cells associates with cortical lineage imbalance

To pinpoint early, mutation-shared transcriptional defects that could prefigure downstream developmental abnormalities, we first performed bulk differential expression analysis on undifferentiated hESCs carrying the R133C, R168X or R270X *MECP2* alleles. This comparison yielded two compact, convergent gene sets with 143 transcripts down-regulated and 139 up-regulated in all mutants relative to WT (Figure 4A). Among the most strongly repressed genes was *EMX1*, a homeobox transcription factor that specifies dorsal telencephalic progenitors (Figure 4A) (Chou et al., 2009; O'Leary et al., 2007). Quantitative RT-PCR at the ESC stage confirmed significant *EMX1* reduction in all three mutant lines relative to WT, and this pattern was reproduced in two independent CRISPR-edited hESC clone series (Figures 4B and S6). *EMX1* expression was likewise decreased in an isogenic iPSC line in which *MECP2* was fully knocked out (Figure 4C), indicating that *EMX1* downregulation is a recurrent feature of *MECP2* loss across distinct human pluripotent backgrounds. To place EMX1 within a developmental framework, we mined reference single-cell atlases spanning mouse embryonic cortex (E12–E15), human fetal cortex (8–20 weeks), and human brain organoids (10 weeks) (Klingler et al., 2021). In all three systems, *EMX1* transcripts are restricted to apical radial glia and nascent glutamatergic neurons, positioning *EMX1* near the apex of cortical excitatory lineage specification (Figure 4D). This observation prompted us to ask whether early EMX1 dysregulation is accompanied by altered lineage allocation at later stages. We therefore generated three-month unguided cerebral organoids from each genotype and profiled them using single-nucleus RNA sequencing, an age when glutamatergic and GABAergic neurons, as well as multiple glial lineages, are readily detectable. UMAP projection separated nuclei into six canonical populations (outer radial glia (oRG), intermediate progenitor cells (IPCs), oligodendrocyte lineage, oligodendrocyte progenitor cells (OPCs), glutamatergic neuron lineage, and inhibitory neurons) validated by the expression of established markers including *SATB2*, *DCX*, *SLC17A7*, *NEUROD2/6*, *TBR1*, *GAD1,* and *PDGFRA* (Figures 4E and S7). Cell-type quantification revealed pronounced, mutation-specific shifts in lineage allocation compared with WT (Figure 4F). Control organoids were dominated by oligodendrocyte-lineage and OPC populations, with relatively modest fractions of inhibitory and glutamatergic neuron lineage cells. In contrast, R133C organoids exhibited an expanded inhibitory compartment

(B) Quantitative RT-PCR validation of *EMX1* expression in patient-derived isogenic iPSC lines expressing either *MECP2-WT*, *MECP2-R133C*, *MECP2-R168X,* or *MECP2-R270X* transcripts. Bars represent mean ± s.e.m.

(C) Quantitative RT-PCR validation of *EMX1* expression in a WT iPSC line and its isogenic line KO for *MECP2* alleles. Bars represent mean ± s.e.m.

(D) Reference single-cell atlases show the spatial localization of *EMX1* transcripts (teal-brown scale) in mouse embryonic cortex (E12–E15), human fetal cortex (8–20 weeks), and 10-week brain organoids. aRG, apical radial glia; IPC, intermediate progenitor cell; iN, immature neuron; mN, mature neuron.

(E) Left, UMAP embedding of 25,132 single nuclei from three-month cerebral organoids ($n$ = 3 per genotype), colored by genotype and annotated into six major populations: outer radial glia (oRG), intermediate progenitor cells (IPC), excitatory neurons, inhibitory neurons, oligodendrocyte, oligodendrocyte progenitor cells (OPC), and astrocytes. Right, feature plots of lineage markers *DCX* (excitatory), *GAD1* (inhibitory), and *MAP2* (pan-neuronal).

(F) Stacked bar chart displays the proportional composition of each cell type per organoid across WT and the three mutant genotypes.

and reduced oligodendroglial representation; R168X organoids accumulated OPC/oligodendrocyte progenitors while retaining very few glutamatergic lineage cells; and R270X organoids showed a marked predominance of glutamatergic neuron lineage cells with comparatively sparse glial populations. These data indicate that *MECP2* mutations perturb cortical cell-type balance in distinct, allele-specific patterns. Together with the convergent *EMX1* repression observed in pluripotent cells, these findings delineate a coherent trajectory in which early MECP2-dependent transcriptional lesions, including mis-timed *EMX1* expression, are associated with biased cortical lineage outcomes in Rett-associated genotypes.

## DISCUSSION

By integrating stage-resolved bulk and single-cell transcriptomics with functional assays in isogenic human ESCs, Rett patient-derived iPSCs, and long-term cerebral organoids, we identify a shared, MECP2-dependent transcriptional program that is already perturbed at the pluripotent stage. Three convergent features emerge. First, a discrete set of 282 transcripts is consistently mis-regulated across three representative *MECP2* mutations, indicating that MECP2 influences a restricted yet coherent gene network even before neural induction. Second, truncating alleles (R168X, R270X) elicit a partial naïve-like molecular signature, marked by *ZFP42*, *PRDM14,* and *SALL4* elevation and accompanied by increased proliferative capacity, without full conversion to bona fide naive pluripotency. Third, all mutants display an abnormal developmental trajectory of the dorsal telencephalic determinant *EMX1*, with early repression in pluripotent cells and altered expression at later precursor stages, and these early lesions are associated with mutation-specific shifts in glutamatergic, inhibitory, and glial lineage proportions in three-month cerebral organoids. Collectively, our data situate the origin of RTT-relevant defects at the pluripotent/early progenitor stages and outline trajectories through which early transcriptional noise may culminate in cortical-circuit imbalance.

### Early transcriptional disruption and its developmental implications

The detection of genotype-specific variance on principal component 2 as early as the ESC stage (Figure 1B) indicates that MECP2 influences transcription prior to neural induction, in a context where global DNA methylation levels are lower than in differentiated neurons. This is consistent with recent reports that MECP2 can bind enhancer-like hotspots and hypomethylated regions, but our study does not directly measure DNA methylation or MECP2 occupancy and therefore cannot distinguish methylation-dependent from methylation-independent mechanisms (Liu et al., 2024; Mishra et al., 2025). The convergent mis-regulation of S100A6 and SLC17A7 across all stages (Figure 1C) further suggests that cytoskeletal organization, cell-state stability, and glutamatergic signaling are primed for later dysfunction before lineage commitment occurs. Our machine-learning classifiers, trained on bulk RNA-seq trajectories, readily decoded developmental stage and highlighted a subset of neuronal genes whose expression followed an "early-low-late-high" pattern in mutants compared with WT (Figures 2 and S2), suggesting mistimed engagement of neurogenic programs. Such temporal misalignment may help reconcile seemingly contradictory findings in mouse models in which *Mecp2* loss produces both premature and delayed aspects of neuronal maturation (Chen et al., 2015; Smrt et al., 2007). A full understanding of these dynamics will require time-resolved chromatin profiling and base-resolution methylome maps in the same lines, ideally coupled to MECP2 ChIP/CUT&Tag, to test whether the observed transcriptomic shifts reflect direct MECP2 binding or secondary remodeling of the epigenetic landscape.

### A partial shift toward a naïve-like pluripotent state

The up-regulation of *ZFP42*, *PRDM14*, *TACSTD2,* and other naïve-enriched markers, together with the increased growth rate of mutant colonies, points to a partial drift from a purely primed toward a more naïve-like pluripotent state. Naive human PSCs are characterized by the elevated expression of these factors, altered metabolic profiles, and distinct epigenetic features, including global DNA hypomethylation and reduced H3K27me3 deposition (Theunissen et al., 2016). In our models, we observe robust induction of *ZFP42*/REX1 and *PRDM14* in truncating mutants, reproducible across two independent CRISPR-edited hESC clones and mirrored in *MECP2*-KO and mutant iPSC lines (Figures 3, S3, and S6), as well as a modest but consistent proliferation advantage (Figure S4C). At the same time, canonical pluripotency factors remain stable, inferCNV and long-read WGS reveal no recurrent structural abnormalities (Figures S4 and S5), and *MECP2*-mutant hESCs fail to self-renew in LIF-based, FGF/TGFβ-free conditions that support bona fide naive PSCs (Figures S3C and S3D). Taken together, these observations argue that *MECP2* loss induces a naïve-like transcriptional bias within an otherwise intact pluripotent framework, rather than triggering a complete reset to a ground-state pluripotency. Whether this bias reflects a cell-autonomous role of MECP2 in stabilizing the primed state, or arises from subtle culture-selection advantages conferred by increased proliferation, remains to be determined. Future work should combine functional assays of naive competence (e.g., trophectoderm potential, transposon activation profiles), global DNA/histone methylation measurements, and single-cell

lineage tracing to refine how MECP2 shapes the primed-naïve spectrum in human pluripotent cells.

### EMX1 as an early bottleneck for cortical excitatory fate

Among the 143 convergently down-regulated genes, *EMX1* stands out because of its highly restricted expression in apical radial glia and nascent glutamatergic neurons across mouse, human fetal cortex, and organoid atlases (Figure 4D). In our system, *EMX1* transcripts are reduced in *MECP2*-mutant hESCs and in two independent CRISPR-edited clone series, as well as in *MECP2*-KO iPSCs (Figures 4B, 4C, and S6), and follow an abnormal neuronal trajectory compared with WT. Although our current data are limited to mRNA, the consistency of *EMX1* repression across alleles and pluripotent backgrounds suggests that EMX1 lies within a core MECP2-dependent developmental program. Single-nucleus RNA-seq in three-month cerebral organoids further shows that MECP2 mutations are associated with allele-specific but convergent perturbations of cortical lineage composition, with altered proportions of glutamatergic neuron lineage, inhibitory neurons, and oligodendrocyte-lineage cells (Figures 4E, 4F, and S7). These findings are compatible with mouse studies in which *Emx1* loss biases cortical progenitors toward ventral or glial fates (Shinozaki et al., 2002) but do not by themselves prove that EMX1 mediates the organoid phenotypes. We therefore view EMX1 as a candidate early bottleneck for cortical excitatory fate in *MECP2*-deficient cells. Demonstrating causality will require stage-specific *EMX1* gain- and loss-of-function in *MECP2*-mutant ESCs and organoids, coupled to protein-level readouts and long-term lineage tracing. Single-cell multi-omics combining chromatin accessibility, MECP2 occupancy, and EMX1 transcription will also be important to distinguish direct from indirect regulation.

### Translational outlook

On the therapeutic front, the partial naïve-like drift and early EMX1 dysregulation raise the possibility that interventions targeting chromatin state or lineage-bias pathways might complement post-natal gene-replacement strategies. Epigenetic drugs or small molecules that stabilize primed pluripotency, modulate MECP2-regulated enhancer networks, or fine-tune EMX1 and related cortical fate determinants could, in principle, normalize aspects of lineage allocation if applied within appropriate developmental windows. Whether such approaches are feasible and safe *in vivo* remains an open question, particularly given the challenges of timing, mosaic X-inactivation in female patients, and potential off-target effects on other brain regions. Our work provides a framework and candidate molecular nodes (naïve-like regulators and EMX1) for future mechanistic and preclinical studies.

### Limitations of the study

The concept that neurodevelopmental disorders originate from perturbations in pluripotent or early progenitor states is gaining traction in Fragile X, 22q11.2 deletion syndrome, and autism spectrum disorder models (Ardhanareeswaran et al., 2017; C. Li et al., 2023; Schafer et al., 2019). Our findings position RTT within this framework and suggest that therapeutic windows may extend into very early developmental stages. However, translating these insights to patients will require caution. Our work relies on human ESCs, iPSCs and cerebral organoids, which offer genetically controlled systems but do not fully reproduce the *in vivo* environment. Morphogen gradients, vascularization, long-range connectivity, and systemic influences are only partially modeled, and our organoid analyses were performed at a single late time point (three months). Additional time points and *in vivo* validation in models carrying the same *MECP2* alleles will be important to confirm how early the described defects arise and how they evolve over development.

A second limitation is that many of our conclusions are based on transcriptomic readouts. We did not yet combine these datasets with genome-wide measurements of DNA and histone modifications or direct MECP2 occupancy, so the precise epigenetic mechanisms underlying the observed changes remain unresolved. Likewise, EMX1 is currently supported as a candidate early bottleneck for cortical excitatory fate by convergent mRNA-level data across *MECP2*-mutant ESC and iPSC models, including lines with controlled X chromosome inactivation, but we have not yet performed stage-specific EMX1 gain- or loss-of-function, protein-level quantification, or multi-omic assays that would establish causality and direct regulation.

Finally, the naïve-like drift and lineage imbalances we describe are inferred from transcriptional signatures, growth kinetics, and snRNA-seq-based cell-type annotation, without the systematic validation of naive functional properties or quantitative immunohistochemistry on matched organoids. Future studies combining epigenomic profiling, protein-level analyses, functional rescue experiments, and electrophysiological characterization will be required to fully define how early MECP2-dependent lesions translate into cortical circuit dysfunction in RTT.

## RESOURCE AVAILABILITY

### Lead contact

Lead contact: Anthony Flamier, anthony.flamier@umontreal.ca.

### Materials availability

All unique, stable reagents generated in this study are available from the lead contact upon reasonable request, contingent on the completion of a Materials Transfer Agreement.

### Data and code availability

- Genomic data are available through a public repository (bulk RNAseq: GSE303838; scRNAseq: GSE303813; snRNAseq: GSE303977).
- All other raw data and codes are available upon request to the lead contact.

## ACKNOWLEDGMENTS

We thank Dr. Rudolf Jaenisch and his team for sharing key reagents and providing strategic guidance. We thank Drs. Graziella Di Cristo, Elsa Rossignol, and Serge McGraw for their insightful guidance; Basma Benabdallah and the CHU Sainte-Justine iPSC core facility; and Nicholas Geoffrion and the bioinformatics core facility for essential technical support. This work was funded by the Canada Brain Research Fund (CBRF)—a partnership between Health Canada and the Brain Canada Foundation (Future Leaders Program); the Azrieli Foundation; the Canadian Institutes of Health Research; the Canadian Stem Cell Network Jump Start ECR Program; the CHU Sainte-Justine Foundation; the Fonds de Recherche du Québec-Santé (FRQS); and the Rett Syndrome Research Trust (for providing Rett iPSC lines). Additional support was provided by the Fonds UdeM pour le partenariat CHU Sainte-Justine -Institut Imagine en épilepsie de l'enfant. We also thank the International Rett Syndrome Foundation (IRSF) Research Independence Award (to Y.L.).

## AUTHOR CONTRIBUTIONS

A.F. conceived the study, secured funding, and provided overall scientific leadership and project coordination (conceptualization, funding acquisition, project administration). M.G., M.B. (Guillon), J.G., L.L. (Laurent), T.R., E.G., M.B. (Brin), J.K., A.B., L.B., M.V., and A.F. each made substantial contributions to the generation, curation, and quality control of the datasets and to the execution of the experimental work; collectively, they performed the majority of wet-lab experiments, validated key findings, and produced the primary data visualizations and figure materials (investigation, data curation, validation, visualization). Y.L. provided essential reagents and cell resources, maintained and quality-controlled cell lines, and contributed directly to experimental execution (resources, investigation). L.L. (Laurent), L.A., and A.F. provided day-to-day scientific oversight, supervised personnel and experimental workflows, and contributed critical interpretation and strategic guidance throughout the study (supervision). A.F. drafted the manuscript, and all authors contributed to manuscript development by critically revising the text, figures, and interpretation, and approving the final submitted version (writing – original draft; writing – review and editing). All authors read and approved the manuscript.

CRediT taxonomy: conceptualization: A.F. methodology: A.F.; M.G.; M.B. (Guillon); J.G.; L.L. (Laurent); T.R.; E.G.; M.B. (Brin); J.K.; A.B.; L.B.; M.V.; Y.L.; L.A. investigation: M.G.; M.B. (Guillon); J.G.; L.L. (Laurent); T.R.; E.G.; M.B. (Brin); J.K.; A.B.; L.B.; M.V.; Y.L.; A.F. resources: Y.L.; A.F. data curation: M.G.; M.B. (Guillon); J.G.; L.L. (Laurent); T.R.; E.G.; M.B. (Brin); J.K.; A.B.; L.B.; M.V.; A.F. formal analysis: A.F.; M.G.; M.B. (Guillon); J.G.; L.L. (Laurent); T.R.; E.G.; M.B. (Brin); J.K.; A.B.; L.B.; M.V. validation: M.G.; M.B. (Guillon); J.G.; L.L. (Laurent); T.R.; E.G.; M.B. (Brin); J.K.; A.B.; L.B.; M.V.; A.F. visualization: M.G.; M.B. (Guillon); J.G.; L.L. (Laurent); T.R.; E.G.; M.B. (Brin); J.K.; A.B.; L.B.; M.V.; A.F. supervision: A.F.; L.L. (Laurent); L.A. project administration: A.F. funding acquisition: A.F. writing – original draft: A.F. writing – review and editing: All authors.

## DECLARATION OF INTERESTS

A.F. is a co-founder and shareholder of StemAxon. The other authors declare no competing interests.

## DECLARATION OF GENERATIVE AI AND AI-ASSISTED TECHNOLOGIES IN THE WRITING PROCESS

During the preparation of this work, the authors used OpenAI ChatGPT Large Language Model o3 to enhance the clarity of the text. The authors reviewed and edited the content and take full responsibility.

## STAR★METHODS

Detailed methods are provided in the online version of this paper and include the following:

- KEY RESOURCES TABLE
- EXPERIMENTAL MODEL AND STUDY PARTICIPANT DETAILS
- METHOD DETAILS
  - Pluripotent stem cell culture
  - Generation of MECP2 KO iPS cells
  - Neuronal differentiation
  - Cell-growth assay
  - Unguided cerebral-organoid differentiation
  - Reverse-transcription quantitative PCR (RT-qPCR)
  - Bulk RNA sequencing
  - Bulk RNA sequencing analysis
  - Deep learning models
  - Single-cell RNA sequencing
  - Single-nucleus RNA sequencing
  - Single-cell and single-nucleus RNA-seq data processing and analysis
  - Whole-genome long-read sequencing (Nanopore)
  - Spontaneous differentiation assay
  - Western blot
  - Cell cycle analysis
- QUANTIFICATION AND STATISTICAL ANALYSIS
  - Randomization, blinding, and sample-size considerations
  - Inclusion and exclusion criteria
  - Definition of significance

## SUPPLEMENTAL INFORMATION

Supplemental information can be found online at https://doi.org/10.1016/j.stemcr.2026.102895.

Received: February 23, 2026
Revised: March 24, 2026
Accepted: March 25, 2026
Published: April 23, 2026

## REFERENCES


Amir, R.E., Van den Veyver, I.B., Wan, M., Tran, C.Q., Francke, U., and Zoghbi, H.Y. (1999). Rett syndrome is caused by mutations in X-linked MECP2, encoding methyl-CpG-binding protein 2. Nat. Genet. *23*, 185–188. https://doi.org/10.1038/13810.

Ardhanareeswaran, K., Mariani, J., Coppola, G., Abyzov, A., and Vaccarino, F.M. (2017). Human induced pluripotent stem cells for modeling neurodevelopmental disorders. Nat. Rev. Neurol. *13*, 265–278. https://doi.org/10.1038/nrneurol.2017.45.

Armstrong, D., Dunn, J.K., Antalffy, B., and Trivedi, R. (1995). Selective dendritic alterations in the cortex of Rett syndrome. J. Neuropathol. Exp. Neurol. *54*, 195–201. https://doi.org/10.1097/00005072-199503000-00006.

Atkins, A., Xu, M.J., Li, M., Rogers, N.P., Pryzhkova, M.V., and Jordan, P.W. (2020). SMC5/6 is required for replication fork stability and faithful chromosome segregation during neurogenesis. eLife *9*, e61171. https://doi.org/10.7554/eLife.61171.

Bajikar, S.S., Zhou, J., O'Hara, R., Tirumala, H.P., Durham, M.A., Trostle, A.J., Dias, M., Shao, Y., Chen, H., Wang, W., et al. (2025). Acute MeCP2 loss in adult mice reveals transcriptional and chromatin changes that precede neurological dysfunction and inform pathogenesis. Neuron *113*, 380–395.e8. https://doi.org/10.1016/j.neuron.2024.11.006.

Breiman, L. (2001). Random Forests. Mach. Learn. *45*, 5–32. https://doi.org/10.1023/A:1010933404324.

Chahrour, M., Jung, S.Y., Shaw, C., Zhou, X., Wong, S.T.C., Qin, J., and Zoghbi, H.Y. (2008). MeCP2, a key contributor to neurological disease, activates and represses transcription. Science (New York, N.Y.) *320*, 1224–1229. https://doi.org/10.1126/science.1153252.

Chao, H.-T., Zoghbi, H.Y., and Rosenmund, C. (2007). MeCP2 Controls Excitatory Synaptic Strength by Regulating Glutamatergic Synapse Number. Neuron *56*, 58–65. https://doi.org/10.1016/j.neuron.2007.08.018.

Chen, L., Chen, K., Lavery, L.A., Baker, S.A., Shaw, C.A., Li, W., and Zoghbi, H.Y. (2015). MeCP2 binds to non-CG methylated DNA as neurons mature, influencing transcription and the timing of onset for Rett syndrome. Proc. Natl. Acad. Sci. USA *112*, 5509–5514. https://doi.org/10.1073/pnas.1505909112.

Cheret, C., Ganzella, M., Preobraschenski, J., Jahn, R., and Ahnert-Hilger, G. (2021). Vesicular Glutamate Transporters (SLCA17 A6, 7, 8) Control Synaptic Phosphate Levels. Cell Rep. *34*, 108623. https://doi.org/10.1016/j.celrep.2020.108623.

Chou, S.-J., Perez-Garcia, C.G., Kroll, T.T., and O'Leary, D.D.M. (2009). Lhx2 specifies regional fate in Emx1 lineage of telencephalic progenitors generating cerebral cortex. Nat. Neurosci. *12*, 1381–1389. https://doi.org/10.1038/nn.2427.

Dani, V.S., Chang, Q., Maffei, A., Turrigiano, G.G., Jaenisch, R., and Nelson, S.B. (2005). Reduced cortical activity due to a shift in the balance between excitation and inhibition in a mouse model of Rett syndrome. Proc. Natl. Acad. Sci. USA *102*, 12560–12565. https://doi.org/10.1073/pnas.0506071102.

Dobin, A., Davis, C.A., Schlesinger, F., Drenkow, J., Zaleski, C., Jha, S., Batut, P., Chaisson, M., and Gingeras, T.R. (2013). STAR: Ultrafast universal RNA-seq aligner. Bioinformatics *29*, 15–21. https://doi.org/10.1093/bioinformatics/bts635.

Donato, R., Sorci, G., and Giambanco, I. (2017). S100A6 protein: Functional roles. Cell. Mol. Life Sci. *74*, 2749–2760. https://doi.org/10.1007/s00018-017-2526-9.

Ghimire, S., Van der Jeught, M., Neupane, J., Roost, M.S., Anckaert, J., Popovic, M., Van Nieuwerburgh, F., Mestdagh, P., Vandesompele, J., Deforce, D., et al. (2018). Comparative analysis of naive, primed and ground state pluripotency in mouse embryonic stem cells originating from the same genetic background. Sci. Rep. *8*, 5884. https://doi.org/10.1038/s41598-018-24051-5.

Guo, D., Liu, Z., Zhou, J., Ke, C., and Li, D. (2024). Significance of Programmed Cell Death Pathways in Neurodegenerative Diseases. Int. J. Mol. Sci. *25*, 9947. https://doi.org/10.3390/ijms25189947.

Guo, H., Zhu, P., Yan, L., Li, R., Hu, B., Lian, Y., Yan, J., Ren, X., Lin, S., Li, J., et al. (2014). The DNA methylation landscape of human early embryos. Nature *511*, 606–610. https://doi.org/10.1038/nature13544.

Guy, J., Hendrich, B., Holmes, M., Martin, J.E., and Bird, A. (2001). A mouse Mecp2-null mutation causes neurological symptoms that mimic Rett syndrome. Nat. Genet. *27*, 322–326. https://doi.org/10.1038/85899.

Hao, Y., Stuart, T., Kowalski, M.H., Choudhary, S., Hoffman, P., Hartman, A., Srivastava, A., Molla, G., Madad, S., Fernandez-Granda, C., and Satija, R. (2024). Dictionary learning for integrative, multimodal and scalable single-cell analysis. Nat. Biotechnol. *42*, 293–304. https://doi.org/10.1038/s41587-023-01767-y.

Horváth, A., Rona, G., Pagano, M., and Jordan, P.W. (2020). Interaction between NSMCE4A and GPS1 links the SMC5/6 complex to the COP9 signalosome. BMC Mol. Cell Biol. *21*, 36. https://doi.org/10.1186/s12860-020-00278-x.

Ip, J.P.K., Mellios, N., and Sur, M. (2018). Rett syndrome: Insights into genetic, molecular and circuit mechanisms. Nat. Rev. Neurosci. *19*, 368–382. https://doi.org/10.1038/s41583-018-0006-3.

Jurewicz, E., Robaszkiewicz, K., Moraczewska, J., and Filipek, A. (2020). Binding of S100A6 to actin and the actin–tropomyosin complex. Sci. Rep. *10*, 12824. https://doi.org/10.1038/s41598-020-69752-y.

Klingler, E., Francis, F., Jabaudon, D., and Cappello, S. (2021). Mapping the molecular and cellular complexity of cortical malformations. Science *371*, eaba4517. https://doi.org/10.1126/science.aba4517.

Li, A., Shi, D., Xu, B., Wang, J., Tang, Y.-L., Xiao, W., Shen, G., Deng, W., and Zhao, C. (2017). S100A6 promotes cell proliferation in human nasopharyngeal carcinoma via the p38/MAPK signaling pathway. Mol. Carcinog. *56*, 972–984. https://doi.org/10.1002/mc.22563.

Li, C., Cui, Z., Deng, S., Lei, T., Chen, P., and Yang, H. (2023a). Programmed Cell Death Protein 2-like Promotes Inflammation and Oxidative Stress in Vascular Endothelial Cells. ACS Pharmacol. Transl. Sci. *6*, 1453–1470. https://doi.org/10.1021/acsptsci.3c00129.

Li, C., Fleck, J.S., Martins-Costa, C., Burkard, T.R., Themann, J., Stuempflen, M., Peer, A.M., Vertesy, Á., Littleboy, J.B., Esk, C., et al. (2023b). Single-cell brain organoid screening identifies developmental defects in autism. Nature *621*, 373–380. https://doi.org/10.1038/s41586-023-06473-y.

Li, H., Handsaker, B., Wysoker, A., Fennell, T., Ruan, J., Homer, N., Marth, G., Abecasis, G., and Durbin, R.; 1000 Genome Project Data Processing Subgroup (2009). The Sequence Alignment/Map format and SAMtools. Bioinformatics *25*, 2078–2079. https://doi.org/10.1093/bioinformatics/btp352.

Li, Y., Wang, H., Muffat, J., Cheng, A.W., Orlando, D.A., Lovén, J., Kwok, S.-M., Feldman, D.A., Bateup, H.S., Gao, Q., et al. (2013). Global transcriptional and translational repression in human-embryonic-stem-cell-derived Rett syndrome neurons. Cell Stem Cell *13*, 446–458. https://doi.org/10.1016/j.stem.2013.09.001.

Liao, W. (2019). Psychomotor Dysfunction in Rett Syndrome: Insights into the Neurochemical and Circuit Roots. Dev. Neurobiol. *79*, 51–59. https://doi.org/10.1002/dneu.22651.

Liao, Y., Smyth, G.K., and Shi, W. (2014). featureCounts: An efficient general purpose program for assigning sequence reads to genomic features. Bioinformatics *30*, 923–930. https://doi.org/10.1093/bioinformatics/btt656.

Liu, Y., Flamier, A., Bell, G.W., Diao, A.J., Whitfield, T.W., Wang, H.-C., Wu, Y., Schulte, F., Friesen, M., Guo, R., et al. (2024). MECP2 directly interacts with RNA polymerase II to modulate transcription in human neurons. Neuron *112*, 1943–1958.e10. https://doi.org/10.1016/j.neuron.2024.04.007.

Liu, Y., Guo, R., and Jaenisch, R. (2025a). Rett Syndrome: Thinking Beyond Brain Borders. Adv. Exp. Med. Biol. *1477*, 243–263. https://doi.org/10.1007/978-3-031-89525-8_9.

Liu, Y., Whitfield, T.W., Bell, G.W., Guo, R., Flamier, A., Young, R.A., and Jaenisch, R. (2025b). Exploring the complexity of MECP2 function in Rett syndrome. Nat. Rev. Neurosci. *26*, 379–398. https://doi.org/10.1038/s41583-025-00926-1.

Love, M.I., Huber, W., and Anders, S. (2014). Moderated estimation of fold change and dispersion for RNA-seq data with DESeq2. Genome Biol. *15*, 550. https://doi.org/10.1186/s13059-014-0550-8.

Lyst, M.J., Ekiert, R., Ebert, D.H., Merusi, C., Nowak, J., Selfridge, J., Guy, J., Kastan, N.R., Robinson, N.D., de Lima Alves, F., et al. (2013). Rett syndrome mutations abolish the interaction of MeCP2 with the NCoR/SMRT co-repressor. Nat. Neurosci. *16*, 898–902. https://doi.org/10.1038/nn.3434.

Marks, H., Kalkan, T., Menafra, R., Denissov, S., Jones, K., Hofemeister, H., Nichols, J., Kranz, A., Stewart, A.F., Smith, A., and Stunnenberg, H.G. (2012). The Transcriptional and Epigenomic Foundations of Ground State Pluripotency. Cell *149*, 590–604. https://doi.org/10.1016/j.cell.2012.03.026.

Masui, S., Ohtsuka, S., Yagi, R., Takahashi, K., Ko, M.S.H., and Niwa, H. (2008). Rex1/Zfp42 is dispensable for pluripotency in mouse ES cells. BMC Dev. Biol. *8*, 45. https://doi.org/10.1186/1471-213X-8-45.

Mishra, G.P., Sun, E.X., Chin, T., Eckhardt, M., Greenberg, M.E., and Stroud, H. (2025). Interaction of methyl-CpG-binding protein 2 (MeCP2) with distinct enhancers in the mouse cortex. Nat. Neurosci. *28*, 62–71. https://doi.org/10.1038/s41593-024-01808-y.

Moog, U., Smeets, E.E.J., van Roozendaal, K.E.P., Schoenmakers, S., Herbergs, J., Schoonbrood-Lenssen, A.M.J., and Schrander-Stumpel, C.T.R.M. (2003). Neurodevelopmental disorders in males related to the gene causing Rett syndrome in females (MECP2). Eur. J. Paediatr. Neurol. *7*, 5–12. https://doi.org/10.1016/s1090-3798(02)00134-4.

Nan, X., Ng, H.-H., Johnson, C.A., Laherty, C.D., Turner, B.M., Eisenman, R.N., and Bird, A. (1998). Transcriptional repression by the methyl-CpG-binding protein MeCP2 involves a histone deacetylase complex. Nature *393*, 386–389. https://doi.org/10.1038/30764.

Neul, J.L., Fang, P., Barrish, J., Lane, J., Caeg, E.B., Smith, E.O., Zoghbi, H., Percy, A., and Glaze, D.G. (2008). Specific Mutations in Methyl-CpG-Binding Protein 2 Confer Different Severity in Rett Syndrome. Neurology *70*, 1313–1321. https://doi.org/10.1212/01.wnl.0000291011.54508.aa.

Neul, J.L., and Zoghbi, H.Y. (2004). Rett syndrome: A prototypical neurodevelopmental disorder. Neuroscientist *10*, 118–128. https://doi.org/10.1177/1073858403260995.

Nguyen, Q.-A., and Nicoll, R.A. (2018). The GABAA Receptor β Subunit Is Required for Inhibitory Transmission. Neuron *98*, 718–725.e3. https://doi.org/10.1016/j.neuron.2018.03.046.

O'Leary, D.D.M., Chou, S.-J., and Sahara, S. (2007). Area patterning of the mammalian cortex. Neuron *56*, 252–269. https://doi.org/10.1016/j.neuron.2007.10.010.

Petriti, U., Dudman, D.C., Scosyrev, E., and Lopez-Leon, S. (2023). Global prevalence of Rett syndrome: Systematic review and meta-analysis. Syst. Rev. *12*, 5. https://doi.org/10.1186/s13643-023-02169-6.

Raspa, M., Gwaltney, A., Bann, C., von Hehn, J., Benke, T.A., Marsh, E.D., Peters, S.U., Ananth, A., Percy, A.K., and Neul, J.L. (2025). Psychometric Assessment of the Rett Syndrome Caregiver Assessment of Symptom Severity (RCASS). J. Autism Dev. Disord. *55*, 997–1009. https://doi.org/10.1007/s10803-024-06238-0.

Reiss, A.L., Faruque, F., Naidu, S., Abrams, M., Beaty, T., Bryan, R.N., and Moser, H. (1993). Neuroanatomy of Rett syndrome: A volumetric imaging study. Ann. Neurol. *34*, 227–234. https://doi.org/10.1002/ana.410340220.

Schafer, S.T., Paquola, A.C.M., Stern, S., Gosselin, D., Ku, M., Pena, M., Kuret, T.J.M., Liyanage, M., Mansour, A.A., Jaeger, B.N., et al. (2019). Pathological priming causes developmental gene network heterochronicity in autistic subject-derived neurons. Nat. Neurosci. *22*, 243–255. https://doi.org/10.1038/s41593-018-0295-x.

Schindelin, J., Arganda-Carreras, I., Frise, E., Kaynig, V., Longair, M., Pietzsch, T., Preibisch, S., Rueden, C., Saalfeld, S., Schmid, B., et al. (2012). Fiji: An open-source platform for biological-image analysis. Nat. Methods *9*, 676–682. https://doi.org/10.1038/nmeth.2019.

Sharifi, O., Haghani, V., Neier, K.E., Fraga, K.J., Korf, I., Hakam, S.M., Quon, G., Johansen, N., Yasui, D.H., and LaSalle, J.M. (2024). Sex-specific single cell-level transcriptomic signatures of Rett syndrome disease progression. Commun. Biol. *7*, 1292. https://doi.org/10.1038/s42003-024-06990-0.

Shi, W., Wang, H., Pan, G., Geng, Y., Guo, Y., and Pei, D. (2006). Regulation of the pluripotency marker Rex-1 by Nanog and Sox2. J. Biol. Chem. *281*, 23319–23325. https://doi.org/10.1074/jbc.M601811200.

Shinozaki, K., Miyagi, T., Yoshida, M., Miyata, T., Ogawa, M., Aizawa, S., and Suda, Y. (2002). Absence of Cajal-Retzius cells and

subplate neurons associated with defects of tangential cell migration from ganglionic eminence in Emx1/2 double mutant cerebral cortex. Development *129*, 3479–3492. https://doi.org/10.1242/dev.129.14.3479.

Sigel, E., and Steinmann, M.E. (2012). Structure, Function, and Modulation of GABAA Receptors. J. Biol. Chem. *287*, 40224–40231. https://doi.org/10.1074/jbc.R112.386664.

Smrt, R.D., Eaves-Egenes, J., Barkho, B.Z., Santistevan, N.J., Zhao, C., Aimone, J.B., Gage, F.H., and Zhao, X. (2007). Mecp2 deficiency leads to delayed maturation and altered gene expression in hippocampal neurons. Neurobiol. Dis. *27*, 77–89. https://doi.org/10.1016/j.nbd.2007.04.005.

Takahashi, S., Kobayashi, S., and Hiratani, I. (2018). Epigenetic differences between naïve and primed pluripotent stem cells. Cell. Mol. Life Sci. *75*, 1191–1203. https://doi.org/10.1007/s00018-017-2703-x.

Theunissen, T.W., Friedli, M., He, Y., Planet, E., O'Neil, R.C., Markoulaki, S., Pontis, J., Wang, H., Iouranova, A., Imbeault, M., et al. (2016). Molecular Criteria for Defining the Naive Human Pluripotent State. Cell Stem Cell *19*, 502–515. https://doi.org/10.1016/j.stem.2016.06.011.

Wang, Y., Kang, X., Kang, X., and Yang, F. (2023). S100A6: Molecular function and biomarker role. Biomark. Res. *11*, 78. https://doi.org/10.1186/s40364-023-00515-3.

Wogram, E., Sümpelmann, F., Khalil, A., Flamier, A., Fu, D., Bell, G.W., and Jaenisch, R. (2025). Human iPSC-Derived Microglia Integrate Into Cerebral Organoids and Assume an In Vivo-Like Phenotype. Eur J Neurosci. *62*, e70281. https://doi.org/10.1111/ejn.70281.

Yu, G., Wang, L.-G., Han, Y., and He, Q.-Y. (2012). clusterProfiler: An R Package for Comparing Biological Themes Among Gene Clusters. OMICS A J. Integr. Biol. *16*, 284–287. https://doi.org/10.1089/omi.2011.0118.

## STAR★METHODS

### KEY RESOURCES TABLE

| REAGENT or RESOURCE | SOURCE | IDENTIFIER |
|---|---|---|
| **Antibodies** | | |
| Anti-GAPDH antibody EPR16891 | Abcam | ab181602; RRID: AB_2630358 |
| Donkey Anti-Mouse IgG H&L (HRP) | Abcam | ab205724 |
| Donkey Anti-Rabbit IgG H&L (HRP) | Abcam | ab205722; RRID: AB_2904602 |
| MAP2 (D5G1) Rabbit Monoclonal Antibody | Cell signaling | 8707; RRID: AB_2722660 |
| Nestin (E409E) Rabbit Monoclonal Antibody | Cell signaling | 73349 |
| Purified anti-Oct4 (Oct3) Antibody | Bio Legend | 653702; RRID: AB_2561767 |
| **Chemicals, peptides, and recombinant proteins** | | |
| Complete Cell Media w/15% FBS Serum and LIF | Sigma-Aldrich | ES-101-B |
| Corning Matrigel Basement Membrane Matrix, LDEV-free | Corning | 354234 |
| Corning Matrigel Growth Factor Reduced (GFR) Basement Membrane Matrix, Phenol Red-free, LDEV-free | Corning | 356231 |
| Dimethyl sulfoxide | Sigma-Aldrich | D8418 |
| DMEM/F12 | Wisent Inc | 319-090 CL |
| Gentle Cell Dissociation Reagent | Stem Cell Technologies | 100-0485 |
| KnockOut Serum Replacement | Gibco | 10828010 |
| mTeSR Plus | Stem Cell Technologies | 100-0276 |
| mTeSR Plus Supplement | Stem Cell Technologies | 100-0275 |
| Pen Strep Glutamine (100X) | Gibco | 10378-016 |
| Plasmocin Prophylactic | InvivoGen | ant-mpp |
| PowerUp SYBR Green Master Mix for qPCR | Applied Biosystems | A25742 |
| QIAshredder | Qiagen | 79656 |
| qScript cDNA SuperMix | Quantabio | 95048-100 |
| ReleSR | Stem Cell Technologies | 100-0483 |
| STEMDiff SMADI Neural Induction Supplement | Stem Cell Technologies | 08580 |
| STEMDiff Neural Induction | Stem Cell Technologies | 05835 |
| Trypan blue Trypan blue | Thermo Fisher | T10282 |
| UltraPure DNase/RNase-Free Distilled Water | Invitrogen™ | 10977-015 |
| Y-27632 (Dihydrochloride) | Stem Cell Technologies | 72304 |

(*Continued on next page*)

*Continued*

| REAGENT or RESOURCE | SOURCE | IDENTIFIER |
|---|---|---|
| **Critical commercial assays** | | |
| Cell Cycle Analysis Kit | Abcam | ab287852 |
| DNA 1000 Kit | Agilent Technologies | 5067-1504 |
| EVERCODE Cell Fixation v2 | Parse Bioscience Inc | ECF2101 |
| EVERCODE Nuclei Fixation v2 | Parse Bioscience Inc | ECF2103 |
| EVERCODE WT Mini v2 | Parse Bioscience Inc | ECW02115 |
| EVERCODE WT v2 | Parse Bioscience Inc | ECW02135 |
| NEBNext High Input Poly(A) mRNA Isolation Module | New England Bioloabs | E3370S |
| NEBNext Ultra II Directional RNA Library Prep Kit for Illumina | New England Bioloabs | E7760L |
| PureLink RNA Mini Kit | Thermo Fisher Scientific | 12183018A |
| QuantiFluor dsDNA system | Promega Corporation | E2670 |
| RNeasy Mini Kit | Qiagen | 74106 |
| STEMDiff Cerebral Organoid Kit | Stem Cell Technologies | 08570 |
| **Oligonucleotides** | | |
| MECP2-KO sgRNA target sequences - forward | Millipore-Sigma | CATCATACATGGGTCCCCGG |
| MECP2-KO sgRNA target sequences - reverse | Millipore-Sigma | CCGAGTCTCTGTTGTCCTGG |
| qPCR_EMX1_F | Millipore-Sigma | ACCGGGACCCTCTCCATTT |
| qPCR_EMX1_R | Millipore-Sigma | GCTTCTGCCGTTTGTACTTTGT |
| qPCR_ZFP42_F | Millipore-Sigma | AGAAACGGGCAAAGACAAGAC |
| qPCR_ZFP42_R | Millipore-Sigma | GCTGACAGGTTCTATTTCCGC |
| qPCR_XIST_F | Millipore-Sigma | CCAGGCAATCTGCTCTGGAA |
| qPCR_XIST_R | Millipore-Sigma | ATGCTGACTACCCAAAGCCC |
| qPCR_POU5F1_F | Millipore-Sigma | CCCCAGGGCCCCATTTTGGTACC |
| qPCR_POU5F1_R | Millipore-Sigma | ACCTCAGTTTGAATGCATGGGAGAGC |
| qPCR_RPLP0_F | Millipore-Sigma | AGCCCAGAACACTGGTCTC |
| qPCR_RPLP0_R | Millipore-Sigma | ACTCAGGATTTCAATGGTGCC |
| qPCR_LEFTY2_F | Millipore-Sigma | GGCCCAGTATGTAGTCCTGC |
| qPCR_LEFTY2_R | Millipore-Sigma | TCCATGCCGAACACCAGC |
| qPCR_PRDM14_F | Millipore-Sigma | AATCATTGGTGGCGACAACGA |
| qPCR_PRDM14_R | Millipore-Sigma | CCCGTACAGAACGAAGTGCAG |
| **Guide RNAs** | | |
| KO-MECP2 sgRNA-1 | EditCo | UGGUGGGCUGAUGGCUGCAC |
| KO-MECP2 sgRNA-2 | EditCo | UCUUCACCUUUUUAAACUUG |
| KO-MECP2 sgRNA-3 | EditCo | GGAAGAAAAGUCAGAAGACC |

(*Continued on next page*)

*Continued*

| REAGENT or RESOURCE | SOURCE | IDENTIFIER |
|---|---|---|
| **Deposited data** | | |
| RNA sequencing | This study | GEO: GSE303838 |
| Single-nucleus RNA sequencing | This study | GEO: GSE303977 |
| Single-cell RNA sequencing | This study | GEO: GSE303813 |
| **Experimental models: Cell lines** | | |
| hESC-WIBR1 R133C MECP2-GFP | Liu et al., (2024) | N/A |
| hESC-WIBR1 R168X MECP2-GFP | Liu et al., (2024) | N/A |
| hESC-WIBR1 R270X MECP2-GFP | Liu et al., (2024) | N/A |
| hESC-WIBR1 WT MECP2-GFP | Liu et al., (2024) | N/A |
| hiPSC PGP-1 | EditCo | N/A |
| hiPSC WT-M | Wogram et al., (2025) | N/A |
| hiPSC WT-F | This study | N/A |
| hiPSC p.R133C | RSRT iPSC Collection Coriell | N/A |
| hiPSC p.R168X | RSRT iPSC Collection Coriell | N/A |
| hiPSC p.R270X | RSRT iPSC Collection Coriell | N/A |
| **Software and algorithms** | | |
| DESeq2 | Love et al., (2014) | https://bioconductor.org/packages/release/bioc/html/DESeq2.html; RRID:SCR_015687 |
| GraphPad Prism GraphPad Software V.10 | N/A | https://www.graphpad.com/scientific-software/prism/; RRID:SCR_002798 |
| Fiji image processing package.76 | Schindelin et al., (2012) | http://fiji.sc; RRID:SCR_002285 |
| featureCounts | Liao et al., (2014) | https://subread.sourceforge.net/featureCounts.html; RRID:SCR_012919 |
| STAR | Dobin et al., (2013) | https://hbctraining.github.io/Intro-to-rnaseq-hpc-02/lessons/03_alignment.html; (RRID:SCR_004463) |
| FASTQC | N/A | https://www.bioinformatics.babraham.ac.uk/projects/fastqc/; (RRID:SCR_014583) |
| SAMtools | Li et al., (2009) | https://www.htslib.org/; RRID:SCR_002105 |
| Seurat V.5 | Hao et al., (2024) | https://satijalab.org/seurat/; (RRID:SCR_016341) |
| Trim Galore! | N/A | https://github.com/FelixKrueger/TrimGalore; RRID:SCR_011847 |
| clusterProfiler | Yu et al., (2012) | https://bioconductor.org/packages/devel/bioc/html/clusterProfiler.html; (RRID:SCR_016884) |
| RandomForest Package in R | Breiman (2001) | https://cran.r-project.org/web/packages/randomForest/index.html; (RRID:SCR_015718) |

(*Continued on next page*)

*Continued*

| REAGENT or RESOURCE | SOURCE | IDENTIFIER |
|---|---|---|
| Keras | N/A | https://github.com/rstudio/keras; (RRID:SCR_026159) |
| TensorFlow | N/A | https://cran.r-project.org/web/packages/tensorflow/index.html; (RRID:SCR_016345) |

## EXPERIMENTAL MODEL AND STUDY PARTICIPANT DETAILS

This study used established human pluripotent stem cell (hPSC) lines and did not recruit living human participants. Three CRISPR/Cas9-edited male WIBR1 hESC lines carrying *MECP2* Rett-associated variants (R133C, R168X, R270X) were analyzed alongside the isogenic WT parental line. Three female patient-derived iPSC lines harboring distinct *MECP2* variants (R133C, R168X, R270X) were obtained from the RSRT iPSC Collection (Coriell Institute). In addition, two control iPSC lines, one male and one female, were included. The experimental design therefore comprised a male isogenic hESC series together with female patient-derived iPSC lines and male and female control iPSC lines (hiPSC WT-M and hiPSC WT-F). The study was not powered or structured to formally test sex- or gender-associated differences in outcomes; therefore, sex-dependent effects could not be systematically assessed and represent a limitation for generalizability beyond the specific lines analyzed. All experimental procedures involving hPSC lines were reviewed and approved by institutional ethics committee (CHU Sainte-Justine).

## METHOD DETAILS

### Pluripotent stem cell culture

Human embryonic stem cells (hESCs) WIBR1 were maintained under feeder-free conditions. Three CRISPR/Cas9-edited male hESC lines carrying the recurrent Rett-syndrome mutations R133C, R168X, and R270X were used alongside the isogenic wild-type (WT) parental line as previously reported (Liu et al., 2024). In addition, three female patient-derived induced pluripotent stem cell (iPSC) lines harbouring distinct *MECP2* variants (R133C, R168X, R270X) were obtained from the RSRT iPSC Collection (Coriell Institute). For routine culture, cells were thawed onto tissue-culture plates coated with hESC-qualified Matrigel (Corning, #354277) and maintained in mTeSR Plus medium (Basal Medium #100-0274 supplemented 1:5 with mTeSR Plus 5× Supplement, #100-2075; STEMCELL Technologies). Cultures were incubated at 37°C in 5 % $CO_2$ and medium was refreshed every 24 h. Cells were passaged every 3–4 days using ReLeSR (STEMCELL Technologies, #100-0483); 10 μM Y-27632 ROCK inhibitor (STEMCELL Technologies, #72308) was added for 24h after passaging to enhance survival. All cell lines were used between passages 20-35 and cultured at a split ratio of 1:6 to 1:10. Freezing and thawing was done using mFreSR reagent (STEMCELL Technologies, #05855) according to manufacturer protocol. Cell lines were monitored daily for evidence of visible contamination and tested every 10 passages for mycoplasma contamination (PCR assay).

### Generation of MECP2 KO iPS cells

PGP-1 iPS cells have been transfected with RNP complex using a mix of 3 gRNA from Editco according to their protocol. Briefly, RNP complexes were assembled during 10 min at room temperature with 7.5:1 sgRNA to Cas9 ratio (i.e. 250pmol sgRNA and 34pmol Cas9). Then 500.000 PGP-1 iPS cells were mixed with the RNP in Lonza P3 Nucleofector solution (Lonza; #V4XP-3032) and transfected using the 4D-nucleofector. Cells were then transferred in a 6-well plate coated with Matrigel hESC (corning; #354277) with mTeSR supplemented with rock inhibitor (10μM). Media was change 24-hr post nucleofection and cells were incubated for 4 days.

DNA were extracted using Quick Extract solution (Mandel scientific; #QE0905T) and amplified by PCR using primers designed against regions of MECP2 flanking the sgRNA target sequences to generate an amplicon of 417 bp. PCR products were sequenced using Sanger Sequencing (Genome Quebec) and indels were identified using ICE analysis software (ICE CRISPR Analysis. 2025. v3.0. EditCo Bio).

### Neuronal differentiation

hESCs were plated on three well of a 6-well plate coated with Growth Factor Reduced Matrigel using ReLeSR (STEMCELL Technologies, #100-0483). After 24h (around 70% confluence) cells were incubated with Neural induction media with SMAD inhibitors (STEMCELL Technologies, #08580) with daily full media change. Differentiated cells were stopped 3 days (Neuroectodermal stage), 7 days (Neural stem cell stage) and 21 days (Neural progenitor cell stage) post induction.

## Cell-growth assay

Individual hESC lines were seeded onto 6-well plates pre-coated with hESC-qualified Matrigel (Corning, #354277). After a 24-h attachment period, plates were transferred to an Incucyte S3 live-cell imager and kept under standard culture conditions (37°C, 5 % $CO_2$). Nine non-overlapping phase-contrast images per well were captured every 4 h for 72 h. For each well three independent colonies of comparable initial size across genotypes were manually selected, and its perimeter was measured at every time point in ImageJ. Growth curves were generated by plotting the perimeter of the same colony over the 72-h imaging window.

## Unguided cerebral-organoid differentiation

Unguided cortical organoids were generated from WT, R133C, R168X and R270X hESCs with the STEMdiff Cerebral Organoid Kit (STEMCELL Technologies, #08570). Briefly, hESCs were dissociated to single cells with ReLeSR, counted, and seeded at $9 \times 10^3$ cells per well in low-attachment, V-bottom 96-well plates supplied with the kit (day 0). Aggregates were cultured in Embryoid Body Medium for 5 days, with half-medium changes on days 2 and 4, to promote uniform spheroid formation. On day 5, spheroids were transferred to neural induction medium in ultra-low-attachment 24-well plates (three spheroids per well). After 48 h (day 7), each spheroid was embedded in a 30-µl dome of growth-factor-reduced Matrigel and moved to maturation medium in a Celtron orbital shaker (INFORS HT, #I69222) operating at 75 rpm inside a humidified incubator (37°C, 5 % $CO_2$). Medium was exchanged every 3–4 days for the duration of the culture. To limit central hypoxia and necrosis, organoids were sectioned under sterile conditions beginning at day 60. Using a Leica VT1000 S vibratome fitted with a sterile blade, each organoid was bisected or trisected (300–400 µm slices) every 2–3 weeks and individual fragments were returned to the shaker in fresh maturation medium. Cultures were harvested at 4 months for downstream single-nucleus RNA-seq and histological analyses.

## Reverse-transcription quantitative PCR (RT-qPCR)

Total RNA was isolated from each hiPSC line in triplicate (n = 3 biological replicates per genotype) with the RNeasy Mini Kit (Qiagen, #74106) on a QIAcube automated workstation. For cDNA synthesis, 500 ng of RNA were reverse-transcribed using qScript cDNA SuperMix (Quantabio, #95048-100) on a SimpliAmp thermal cycler, following the manufacturer's protocol. The resulting cDNA was diluted 1:8 with nuclease-free water and used as template for SYBR Green qPCR. Reactions (10 µL total) were assembled in 96-well plates as follows: 5 µL PowerUp SYBR Green Master Mix (Thermo Fisher Scientific), 0.5 µL forward primer (10 µM), 0.5 µL reverse primer (10 µM), 3 µL UltraPure DNase/RNase-free distilled water, and 1 µL diluted cDNA.

Thermal cycling conditions followed the Master Mix guidelines (two-step protocol with melt-curve analysis). All samples were run in technical triplicate, and relative expression was calculated by the ΔΔCt method after normalization to the geometric mean of *RPLP0*.

## Bulk RNA sequencing

Total RNA was extracted in biological triplicate (n = 3 per line) with the PureLink RNA Mini Kit (Invitrogen, #12183018A) at four matched time points: day 0, day 3, day 7 and day 21 for WT and the three point-mutant lines. Polyadenylated RNA was purified from each triplicate with the NEBNext Poly(A) mRNA Magnetic Isolation Module (New England Biolabs, #E3370S). One microlitre of the eluate was quantified on a NanoDrop spectrophotometer. In total, 48 barcoded libraries were prepared with the NEBNext Ultra II Directional RNA Library Prep Kit for Illumina (NEB, #E7760L) according to the manufacturer's instructions. Libraries were sequenced on Illumina NovaSeq X Plus instruments using paired-end 50-bp reads (PE50) with at least 40 million reads per sample.

## Bulk RNA sequencing analysis

Raw paired-end FASTQ files were quality-checked with FastQC (v0.11.9) and summarised with MultiQC. Adapter sequences and bases with Phred < 20 were removed using Trimmomatic (v0.39; settings ILLUMINACLIP:2:30:10, SLIDINGWINDOW:4:20, MINLEN:36). Cleaned reads were aligned to the GRCh38/hg38 reference (Ensembl 109 annotation) with STAR (v2.7.11a) in two-pass mode (–twopassMode Basic) and sorted BAMs were produced (–outSAMtype BAM SortedByCoordinate). Gene-level counts were obtained with featureCounts (v2.0.2; options -p -B -C –primary -s 0) and imported into R (4.3.2). Low-abundance features (row-sums $\leq$ 1) were discarded, and a DESeq2 (v1.40.2) object was created with the design formula ~ Genotype + Time. After dispersion estimation and Wald testing, contrasts were extracted for each mutant versus WT at each time point; genes with $log_2FC > 2$ and Benjamini–Hochberg–adjusted $p < 0.01$ were considered differentially expressed. Variance-stabilised counts (vst, blind = FALSE) were used for

principal-component analysis (plotPCA) and sample–sample correlation heat-maps (Pearson r, ggplot2 v3.4.4). Differential-expression result tables were exported per comparison, summarised, and filtered lists were subjected to Gene-Ontology over-representation analysis with clusterProfiler (v4.8.3; enrichGO, OrgDb = org.Hs.eg.db v3.17.0, ont = "ALL", padj < 0.05). Transcript-per-million (TPM) values were calculated from raw counts and gene lengths (featureCounts "Length" column) and $log_2$-transformed for marker-gene heat-maps (pheatmap v1.0.12, viridis palette). In Figure 3C, the 32 canonical pluripotency genes based on Ghosh et al., 2020 are: *C1orf210*, *CLDN6*, *ESRP1*, *GLB1L3*, *GYLTL1B*, *HES3*, *KCNG3*, *L1TD1*, *LCK*, *LIN28A*, *MATK*, *NANOG*, *POU5F1*, *PPP1R16B*, *PRDM14*, *PRSS8*, *SCNN1A*, *SLC7A3*, *TDGF1*, *TRIM71*, *VRTN*, *ZSCAN10*, *AC007326.1*, *APELA*, *BEND4*, *CAMKV*, *DPPA4*, *HLA-DOA*, *HTR3A*, *KCNK5*, *PTPRZ1*, *SYT6*.

## Deep learning models

*K-means clustering*. Gene-level TPM values were imported into R (v4.3.2) and filtered to the 10 000 most variable transcripts across all samples (coefficient of variation). Expression values were $log_2$-transformed and centred before clustering with the base kmeans() function, specifying k = 6, nstart = 25 and the default Euclidean distance. Cluster membership was merged with sample metadata for downstream visualisation of cluster-specific trajectories and Gene-Ontology enrichment.

*Random-forest stage classifier*. For supervised classification, only WT samples were used for training. The TPM matrix was transposed so that rows corresponded to samples and columns to genes; metadata columns (Sample, Genotype, Stage) were removed from the feature set. A factor response vector encoded the four developmental stages (ESC, NE, NSC, NPC). A random-forest model was trained with the randomForest package (v4.7-1.1) using ntree = 500, default mtry, and importance = TRUE. Model performance was inspected via the out-of-bag error rate and a confusion matrix. Variable importance (mean decrease in accuracy) was extracted and plotted for the top 25 genes. The trained classifier was applied to mutant samples (R133C, R168X, R270X) to obtain both hard stage calls and class-probability distributions; results were visualised with ggplot2 (v3.4.4). Mis-classifications were summarized and prediction confidence values (maximum class probability) were compared across genotypes.

*Feed-forward neural-network classifier*. A deep neural network was implemented with the keras R interface (keras v2.13.0; TensorFlow v2.14 backend). WT TPM values were $log_2(x + 1)$ transformed, gene-wise z-scored (mean subtraction, division by s.d.), and clipped to ±5. The network architecture comprised an input layer matching the number of genes, two hidden dense layers (64 and 32 ReLU units) each followed by 0.30 dropout, and a 4-unit soft-max output layer. Categorical stage labels were one-hot encoded. The model was compiled with categorical cross-entropy loss, the legacy Adam optimizer (learning rate = $5 \times 10^{-4}$), and accuracy as the metric. Training proceeded for up to 200 epochs with batch_size = 4, using a 25 % validation split; early-stopping (patience = 10) and ReduceLROnPlateau (patience = 5) callbacks prevented over-fitting. After convergence, the model weights with the lowest training loss were retained. Mutant samples were pre-processed with the same scaling parameters and predicted to yield both class labels and confidence scores.

## Single-cell RNA sequencing

Human ESCs (WT, R133C, R168X and R270X) were harvested at the pluripotent stage, dissociated with Gentle Cell Dissociation (Stem Cell Technologies) and resuspended in ice-cold PBS + 0.04 % BSA. For each genotype, cells from three independent wells were pooled, yielding four fixed suspensions in total. Fixation was performed immediately with the EVERCODE Cell Fixation v2 Kit (Parse Biosciences, #ECF2101) according to the manufacturer's protocol. Cell density and viability were assessed by mixing 10 μL of the suspension with 10 μL 0.4 % Trypan Blue (Thermo Fisher, #T10282) and counting on a Countess 3 Automated Cell Counter; all samples exceeded 90 % viability and were adjusted to $1 \times 10^6$ cells $mL^{-1}$. Two single-cell libraries were constructed from the four fixed samples using the EVERCODE WT Mini v2 Kit (Parse Biosciences, #ECW02115), which employs a split-pool combinatorial indexing workflow comprising four rounds of barcoding followed by cDNA synthesis and amplifications. Final libraries were purified with AMPure XP beads, and double-stranded DNA concentration was measured fluorometrically with a Qubit 4 and the QuantiFluor dsDNA System (Promega, #E2670). Both libraries were sequenced on an Illumina NovaSeq 6000 S4 flow-cell using paired-end 150-bp reads (PE150).

## Single-nucleus RNA sequencing

Cerebral organoids were harvested on day 90 from three independent cultures per genotype, and all reagents, consumables and centrifuges were pre-cooled to 4°C. Organoids were transferred to a chilled Dounce containing 700 μL homogenization buffer (NIM1: 250 mM sucrose, 25 mM KCl, 5 mM $MgCl_2$, 10 mM Tris-HCl pH 8, plus 1 mM DTT, 0.40 U $\mu L^{-1}$ RNase-In, 0.20 U $\mu L^{-1}$ Superase-In and 0.1 % Triton X-100) and gently dissociated with 5 loose-pestle and 10 tight-pestle strokes,

then adjusted to 1 mL with the same buffer. Lysis efficiency was confirmed by 1:1 Trypan Blue staining, after which lysates were passed through a 40 μm strainer into pre-cooled 15 mL tubes, centrifuged (600 g, 4 min, 4°C), washed once in PBS containing 0.2 U $\mu L^{-1}$ RNase-In, and re-pelleted under the same conditions. Nuclei were resuspended in 200 μL PBS/RNase-In, re-filtered (40 μm), counted with a Countess 3 Automated Cell Counter (10 μL nuclei + 10 μL 0.4 % Trypan Blue; Thermo Fisher #T10282), and immediately fixed using the Evercode Nuclei Fixation v2 kit (Parse Biosciences, #ECF2103). Eight single-nucleus libraries were then prepared with the EVERCODE™ WT v2 kit (Parse Biosciences, #ECW02135) according to the manufacturer's split-pool protocol. Library yield was quantified with a Qubit fluorometer and QuantiFluor dsDNA system (Promega #E2670), and fragment size distribution verified on an Agilent 2100 Bioanalyzer using the DNA 1000 kit (#5067-1504). Libraries were sequenced on an Illumina NovaSeq 6000 S4 flow-cell using paired-end 150-bp reads (PE150).

### Single-cell and single-nucleus RNA-seq data processing and analysis

Raw paired-end FASTQ files from hESC single-cell (EVERCODE WT-Mini v2) and 3-month organoid single-nucleus (EVERCODE WT v2) libraries were inspected with FastQC (v0.11.9). Demultiplexing, read trimming, alignment to GRCh38/hg38 and transcript counting were performed with the Parse Biosciences command-line pipeline (commit 2024-02-20); the merge module combined the four sub-libraries per run to produce a gene–cell count matrix (.mtx), feature list and cell-level metadata.

Down-stream analyses were conducted in R (4.3.2) with Seurat (v5.0.1). Matrices were imported with ReadParseBio, and cells with >15,000 detected genes, >100,000 UMIs or >15 % mitochondrial UMIs were removed. Counts were log-normalised (NormalizeData, scale.factor = 10,000), 2,000 variable features were selected (FindVariableFeatures, method = "vst"), and expression values were centered and scaled (ScaleData). Dimensionality reduction used PCA (RunPCA); the first 30 principal components (PCs) were retained based on the elbow plot. A shared-nearest-neighbour graph was constructed (FindNeighbors, dims = 1:30) and Louvain clusters identified at resolution = 0.30 (FindClusters). Clusters were reordered by hierarchical tree building (BuildClusterTree) and visualised with UMAP (RunUMAP, dims = 1:30). Cluster markers were identified with FindAllMarkers (min.pct = 0.25, logfc.threshold = 0.25) and visualised by violin, dot, and feature plots. To assess large-scale genomic integrity in the hESC single-cell datasets, inferred copy-number variation (CNV) profiles were generated with inferCNV (R package, default parameters) using WT hESCs as the reference "normal" population. Genes were ordered by genomic position, expression values were smoothed across neighbouring genes, and denoised heatmaps were inspected for clonal gains or losses along chromosomes.

### Whole-genome long-read sequencing (Nanopore)

Genomic DNA was extracted from hESC pellets using the DNeasy Blood & Tissue Kit (Qiagen) according to the manufacturer's instructions. High-molecular-weight DNA (≈1–2 μg per sample) was used to prepare Oxford Nanopore sequencing libraries with the appropriate ligation-based library preparation kit, including native barcoding to allow multiplexing. Four barcoded samples were pooled and loaded on a single P2 flow cell (Oxford Nanopore Technologies) for sequencing. Basecalling was performed with the ONT Guppy pipeline using high-accuracy settings, and reads with low quality were discarded. Filtered reads were aligned to the GRCh38/hg38 reference genome with minimap2, and structural variants as well as large-scale copy-number changes were surveyed using standard long-read SV calling tools.

### Spontaneous differentiation assay

hESC lines were seeded onto 2 wells of a 12-well plate previously coated with hESC-qualifed Matrigel (Corning, #354277). After a 72-h growth period with medium changes, the medium was removed for either Complete Cell Media w/15% FBS Serum and LIF (Sigma-Aldrich, #ES-101-B) or mTeSR Plus medium (Basal Medium #100-0274 supplemented 1:5 with mTeSR Plus 5× Supplement, #100-2075; STEMCELL Technologies). Then, the plate was transferred to an Incucyte S3 live-cell imager with culture condition as described previously. Nine-overlapping phase-contrast images per well were capture every 30 minutes for 6 h.

### Western blot

Total proteins were extracted from WT, R133C, R168X and R270X hES cells at 0 day (ESC), 3 days (NE), 7 days (NSC) and 21 days (NPC) post induction using a RIPA Lysis Buffer, 10X (Sigma-Aldrich, #20-188) containing protease inhibitor cocktail (Sigma #S8820). Denatured proteins (10 μg for NESTIN and 20 μg for MAP2 and POU5F1) were separated by 3-8% Tris-Acetate or 4-12% Bis-Tris gel electrophoresis followed by transfer to nitrocellulose membranes. The membranes were blocked in 5% skim milk for one hour at room temperature, followed by incubation with an anti-NESTIN (CST #73349; 1/1,000), anti-MAP2 antibody (CST #8707; 1/1,000), or anti-POU5F1 antibody (Bio Legend 653702;

1/500) overnight at 4°C. The membranes were then incubated with the secondary antibody, donkey anti-rabbit (ab205722 at 1/10,000) or donkey anti-mouse (ab205724 at 1/10,000) for one hour at room temperature. The band intensities were quantified by densitometry using Image Lab 5.1 software (Bio-Rad). Protein levels were expressed as a ratio of protein-specific band density to GAPDH protein level (Abcam #ab181602 at 1/200,000).

### Cell cycle analysis

The cell cyle of each hESC cell lines was performed using the Cell Cycle Analysis Kit (Abcam, ab287852). Briefly, the hESC cells were harvest using ReLeSR (Stem Cell Technlologies, #100-0483) and centrifuge at 400 x g for 5 minutes. The cell pellet was washed in cold 1X Cell Cycle Assay Buffer, then centrifuge again at 400 x g for 5 min. The cells were fixed using 70% Ethanol and incubate for 30 min at 4°C, then centrifuge at 400 x g for 5 min. The cell pellet was washed in cold 1X Cell Cycle Assay Buffer and centrifuge at 400 x g for 5 min. The pellets were resuspended in the Staining solution and incubate for 30min. The cell cycle stage was analysed with a BD FACSAria™ Fusion Cell Sorter (Plateforme cytométrie at Sainte-Justine hospital).

## QUANTIFICATION AND STATISTICAL ANALYSIS

All statistical procedures, n values, definitions of center, dispersion metrics, and exact *P* or adjusted *P* values are reported in the corresponding figure legends, main text, or Method subsections. A consolidated summary of analytical strategies is provided below.

| Experiment | Software | Statistical test or model | Definition of *n* | Measures of center ± dispersion | Multiple-test correction | Location |
|---|---|---|---|---|---|---|
| RT-qPCR (Figures 3D and 4B) | Prism 10 (GraphPad) | One-way ANOVA with Dunnett post-hoc (mutant vs WT) | Independent iPSC or hESC cultures | Mean ± s.e.m. | — | Figures 3D and 4B legend |
| Cell-growth (Figure 3H) | Incucyte S3 software + Prism 10 | Two-way repeated-measures ANOVA | Colonies tracked over time (9 fields × 3 wells × 3 experiments per genotype) | Mean ± s.e.m. | — | Figure 3H legend |
| Cell-cycle phase counts (Figure 3G) | Seurat 5.0.1 | Pearson $\chi^2$ goodness-of-fit vs WT | Single cells passing QC (exact cell counts in legend) | Proportion (% of cells) | — | Figures 3F and 3G legend |
| Bulk rna-seq DE (Figures 1 and 4) | DESeq2 1.40.2 | Wald test (mutant vs WT) | Independent differentiations (3 per genotype & stage) | $\log_2$ fold-change | Benjamini–Hochberg padj < 0.01 & | $\log_2$FC |
| GO enrichment (Figures 1C and 2C) | clusterProfiler 4.8.3 | Hypergeometric test | Gene universe = all expressed genes | GeneRatio, $-\log_{10}$ padj | Benjamini–Hochberg padj < 0.05 | Methods → "Bulk RNA-seq analysis" |
| Machine learning (Figure 2) | R 4.3.2: stats:: kmeans, randomForest 4.7-1.1, keras 2.13/TensorFlow 2.14 | — | See below | OOB error, prediction probability | 5-fold CV for RF; 25 % validation split for NN | Figure 2 legends |

### Randomization, blinding, and sample-size considerations

hESC and iPSC lines were plated and differentiated in parallel under identical conditions; wells were assigned randomly to imaging positions in Incucyte assays, and image acquisition/analysis were automated. Organoid batches were generated from separate vials per genotype to preserve biological replication. Investigators were not blinded to genotype during cell culture but downstream bioinformatic pipelines were scripted and executed without manual intervention. For exploratory *in vitro* systems we did not perform *a priori* power calculations; instead, sample sizes (three independent differentiations per condition, ≥2 900 single cells per scRNA-seq sample, three organoids per genotype for snRNA-seq) reflect field standards and our prior experience achieving reproducible effect sizes.

### Inclusion and exclusion criteria

For RT-qPCR and growth assays all biological replicates were included. sc/snRNA-seq data were filtered to retain nuclei with <15 % mitochondrial RNA and 500 ≤ nFeature_RNA ≤ 15 000; doublets detected by index hopping were excluded. Bulk RNA-seq genes with ≤1 read across all samples were removed prior to DESeq2.

### Definition of significance

Unless otherwise specified, $P < 0.05$ (after correction where applicable) was considered significant. Exact P, padj, or FDR values are provided in figure panels or legends.

**Stem Cell Reports, Volume *21***

## Supplemental Information

# *MECP2* mutations rewire human ESC fate and bias cortical lineage commitment

**Marion Guillon, Margaux Brin, Elodie Gabet, Justine Gromaire, Mathéa Bernard, Laetitia Laurent, Théo Rabin, Lisa Bianchin, Marie Veziano, Julie Kloda, Alexia Bernard, Laila Asali, Yi Liu, and Anthony Flamier**

## SUPPLEMENTAL INFORMATION

**Document S1. Supplemental Figures S1–S7.**

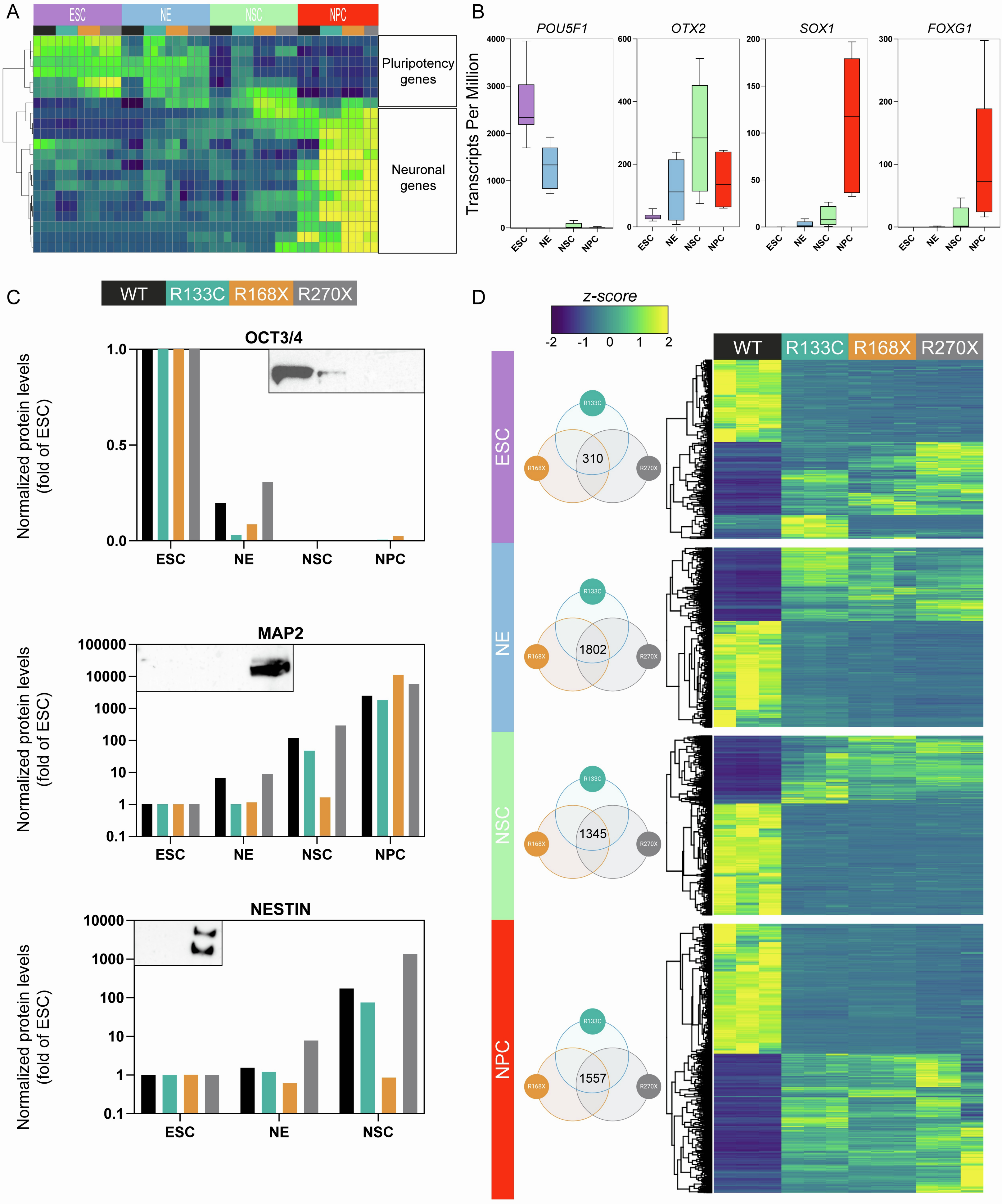

A
ESC
NE
NSC
NPC
Pluripotency genes
Neuronal genes
B
POU5F1
OTX2
SOX1
FOXG1
Transcripts Per Million
C
WT
R133C
R168X
R270X
OCT3/4
MAP2
NESTIN
Normalized protein levels (fold of ESC)
D
z-score
-2 -1 0 1 2
310
1802
1345
1557


Figure S1

**Figure S1.**

**A.** Z-score heat-map of curated pluripotency (top block) and neuronal (bottom block) marker genes across the four differentiation stages (ESC, NE, NSC, NPC; color bar above) for WT and the three mutant hESC lines (key below). Replicate columns are ordered by hierarchical clustering (Ward's method, Euclidean distance).

**B.** Average expression (TPM value) for four stage specific markers, extracted from bulk RNA-seq data.

**C.** Average protein abundance by Western-blot for three stage specific markers for each stage of differentiation.

**D.** For each stage, Venn diagrams (left) enumerate significantly dysregulated genes common to all mutants (Wald test, DESeq2; $log_2FC > 2$, $padj < 0.01$). The corresponding union set is visualized as a heat-map (right; same scaling as in A) with rows clustered by Pearson correlation.

A

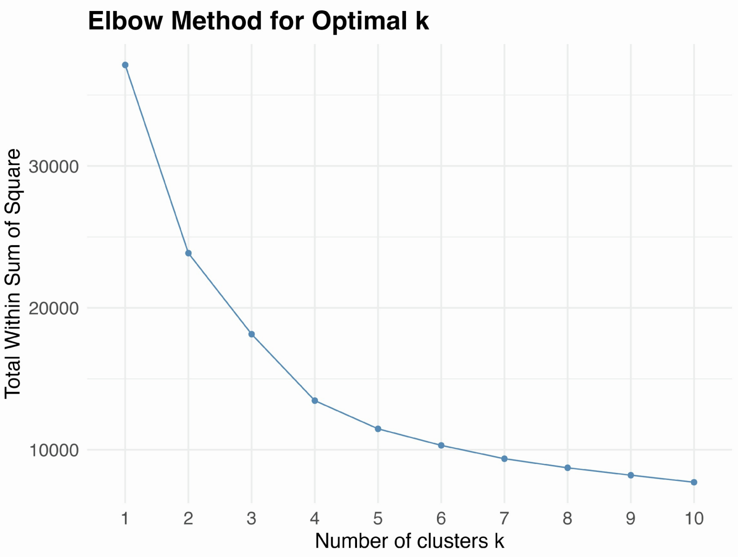

Elbow Method for Optimal k
Total Within Sum of Square
30000
20000
10000
1
2
3
4
5
6
7
8
9
10
Number of clusters k


B

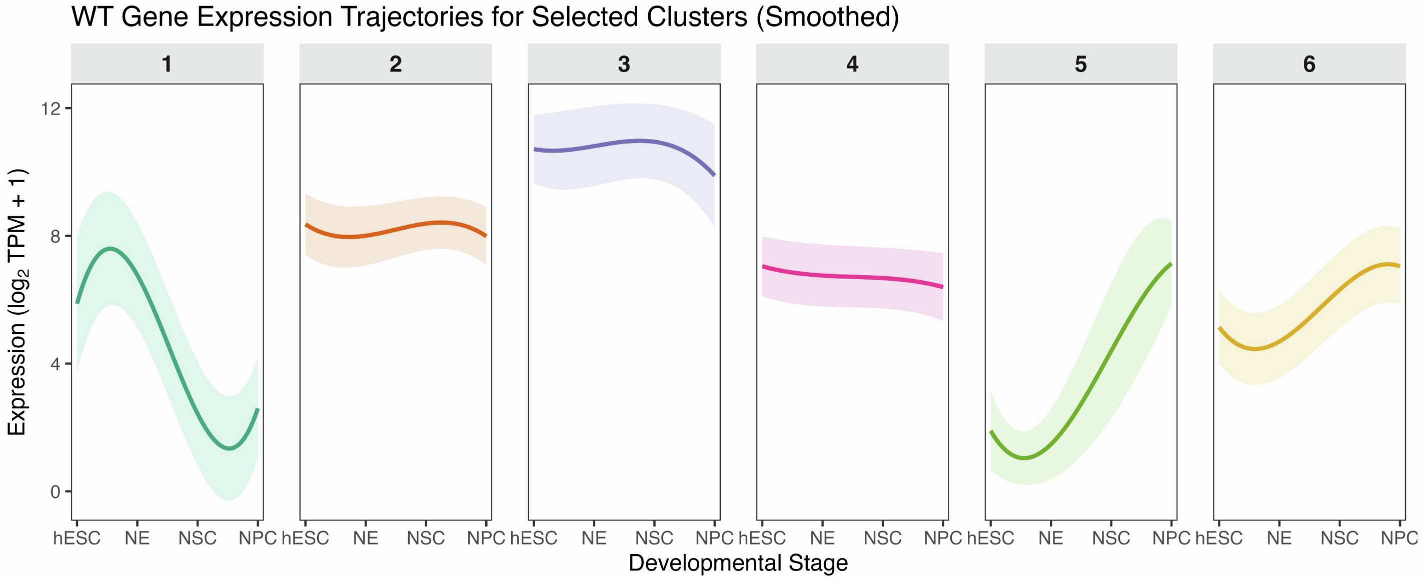

WT Gene Expression Trajectories for Selected Clusters (Smoothed)
1
2
3
4
5
6
12
8
4
0
Expression ($log_2$ TPM + 1)
hESC
NE
NSC
NPC
Developmental Stage


Figure S2

**Figure S2.**
**A.** Elbow plot of total within-cluster sum of squares across k = 1–10; k = 6 was chosen as the inflection point for all further k-means analyses.
**B.** Smoothed (loess) expression trajectories of WT genes belonging to the six selected clusters (shaded 95 % confidence band; y-axis, $\log_2$ TPM + 1; x-axis, developmental stage).

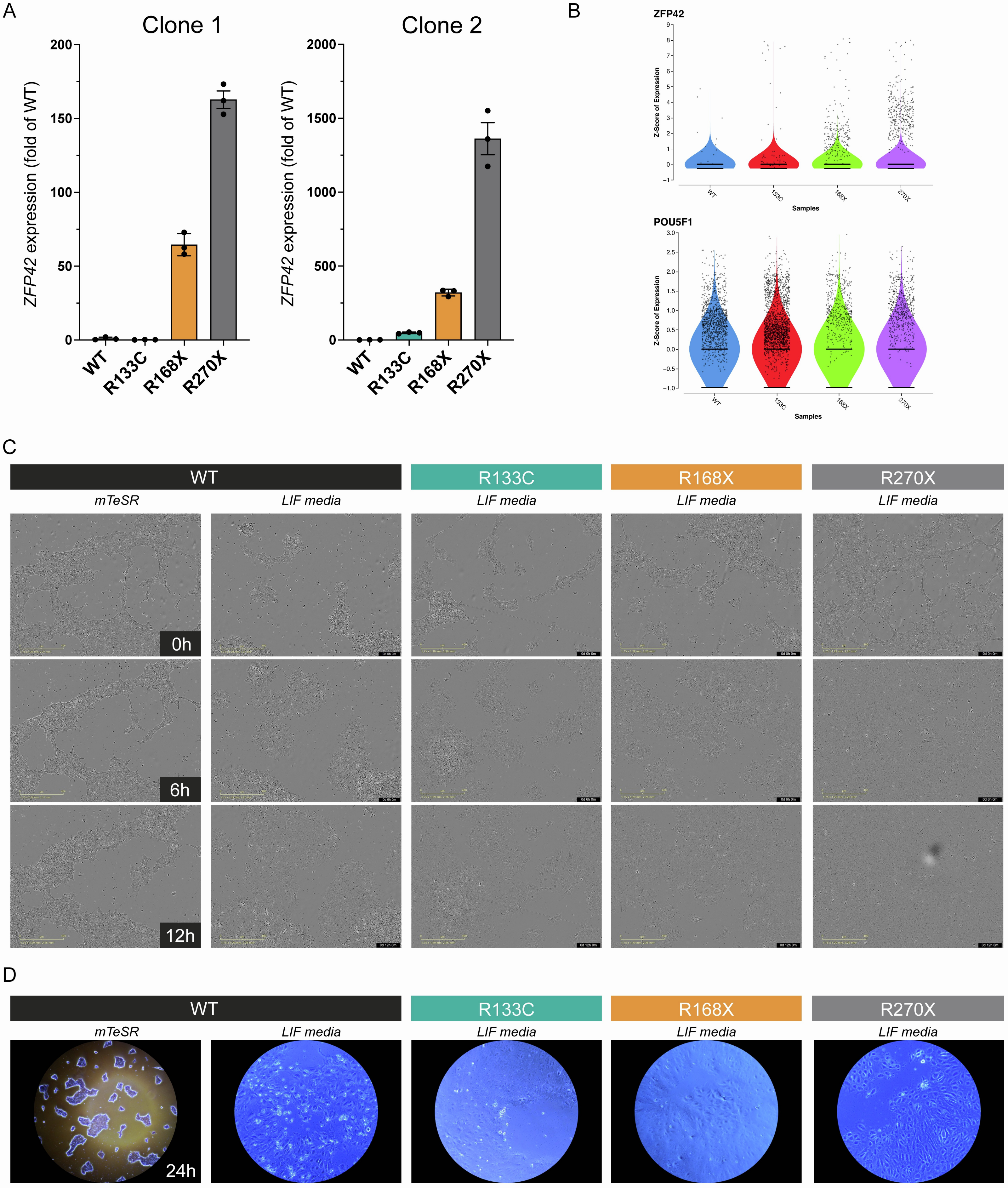
A
Clone 1
ZFP42 expression (fold of WT)
200
150
100
50
0
WT
R133C
R168X
R270X
Clone 2
ZFP42 expression (fold of WT)
2000
1500
1000
500
0
WT
R133C
R168X
R270X
B
ZFP42
Z-Score of Expression
Samples
WT
133C
168X
270X
POU5F1
Z-Score of Expression
Samples
WT
133C
168X
270X
C
WT
R133C
R168X
R270X
mTeSR
LIF media
LIF media
LIF media
LIF media
0h
6h
12h
D
WT
R133C
R168X
R270X
mTeSR
LIF media
LIF media
LIF media
LIF media
24h

Figure S3

**Figure S3.**

**A.** Quantitative RT–PCR validation of *ZFP42/REX1* expression in two independent CRISPR-edited hESC clone series (Clone 1 and Clone 2) expressing *MECP2-WT, MECP2-R133C, MECP2-R168X* or *MECP2-R270X*. Expression is shown as fold change relative to the corresponding WT line (set to 1). Bars represent mean ± s.e.m.

**B.** Violin plots showing single-cell expression distributions of *ZFP42* and *POU5F1* across WT, R133C, R168X and R270X hESCs from scRNA-seq; each dot represents one cell.

**C.** Phase-contrast images of WT hESCs maintained in mTeSR and MECP2-mutant hESCs transferred to LIF-based medium, taken 0, 6 and 12 h after medium switch, illustrating loss of compact pluripotent colony morphology under LIF conditions.

**D.** hESC colonies after 24 h in mTeSR (WT) or LIF-based medium (WT and MECP2-mutant lines), showing failure of both WT and mutant hESCs to sustain naïve-like self-renewal in LIF conditions.

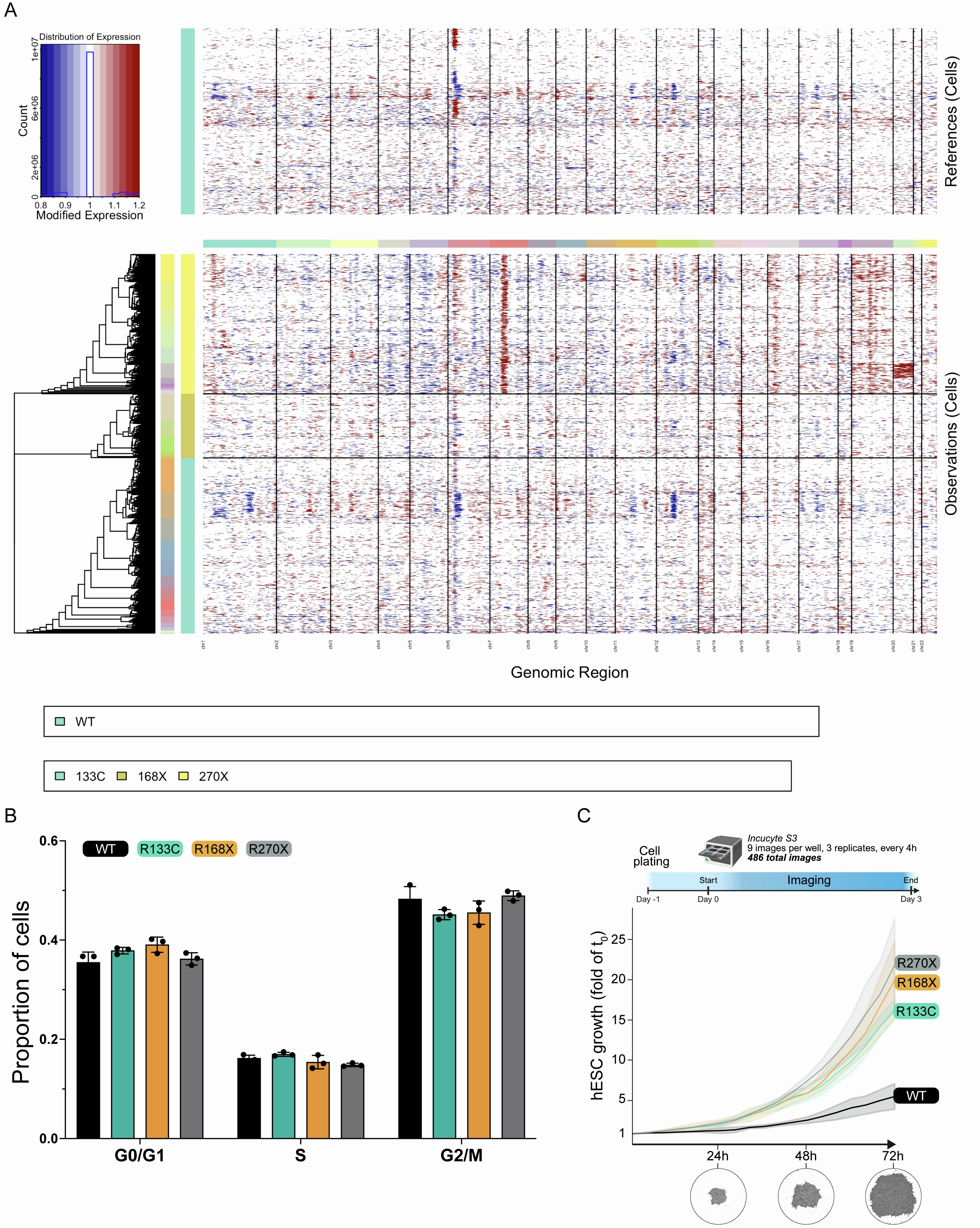
A
Distribution of Expression
Count
1e+07
6e+06
2e+06
0
0.8
0.9
1
1.1
1.2
Modified Expression
References (Cells)
Observations (Cells)
chr1
chr2
chr3
chr4
chr5
chr6
chr7
chr8
chr9
chr10
chr11
chr12
chr13
chr14
chr15
chr16
chr17
chr18
chr19
chr20
chr21
chr22
Genomic Region
WT
133C
168X
270X
B
WT
R133C
R168X
R270X
Proportion of cells
0.6
0.4
0.2
0.0
G0/G1
S
G2/M
C
Cell plating
Incucyte S3
9 images per well, 3 replicates, every 4h
486 total images
Start
Imaging
End
Day -1
Day 0
Day 3
hESC growth (fold of $t_0$)
25
20
15
10
5
1
R270X
R168X
R133C
WT
24h
48h
72h

Figure S4

**Figure S4.**

**A.** Top panel, reference WT cells; bottom panel, combined R133C, R168X and R270X observations. Each column represents a genomic region ordered chromosomally from 1 to Y (black tick marks) and each row a single cell, clustered by Euclidean distance. Red denotes inferred gains, blue denotes losses (scale bar, left). Color strips mark genotype identity of observation cells. Dendrogram shows hierarchical relationships among mutant cells.

**B.** Cell-cycle distribution of WT, R133C, R168X and R270X hESCs determined by flow cytometry. Cells were fixed, DNA content was measured by FACS, and the proportions of cells in G0/G1, S and G2/M phases were quantified. Bars represent mean ± s.e.m. of independent experiments.

**C.** Live-cell imaging proliferation assay, growth curves of hESC colonies over 72 h recorded on an Incucyte S3 (9 images/well, N=3); inset photographs illustrate colony morphology at 24, 48 and 72 h.

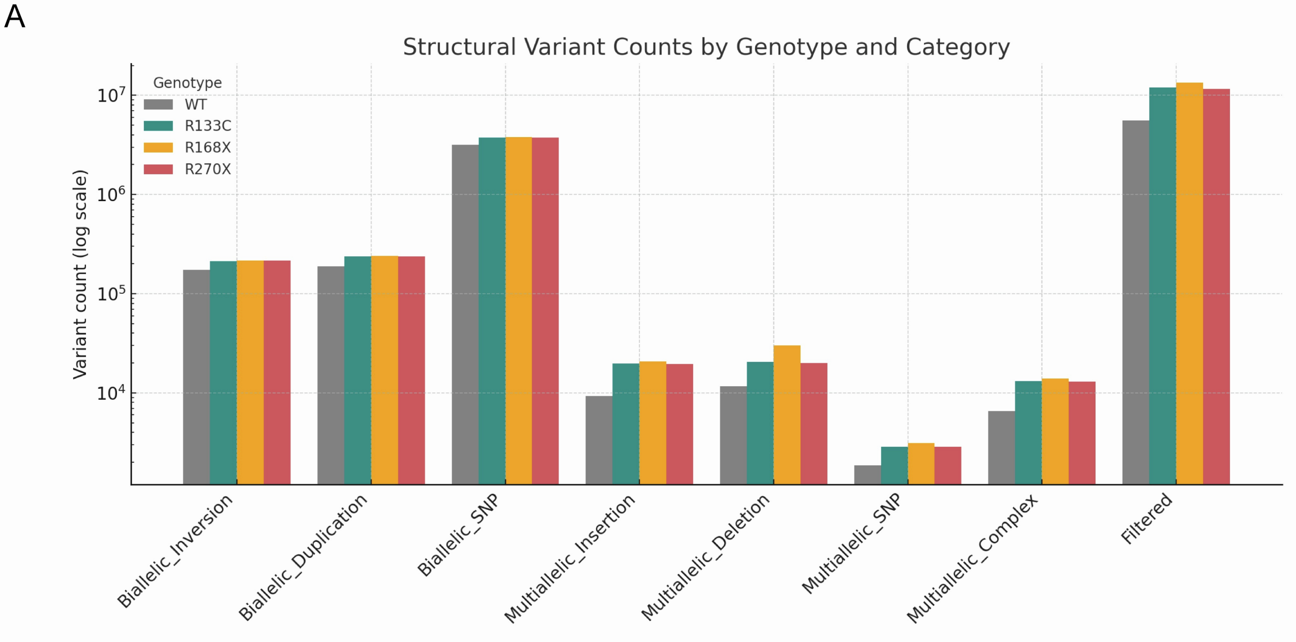
A
Structural Variant Counts by Genotype and Category
Genotype
WT
R133C
R168X
R270X
Variant count (log scale)
10^7
10^6
10^5
10^4
Biallelic_Inversion
Biallelic_Duplication
Biallelic_SNP
Multiallelic_Insertion
Multiallelic_Deletion
Multiallelic_SNP
Multiallelic_Complex
Filtered

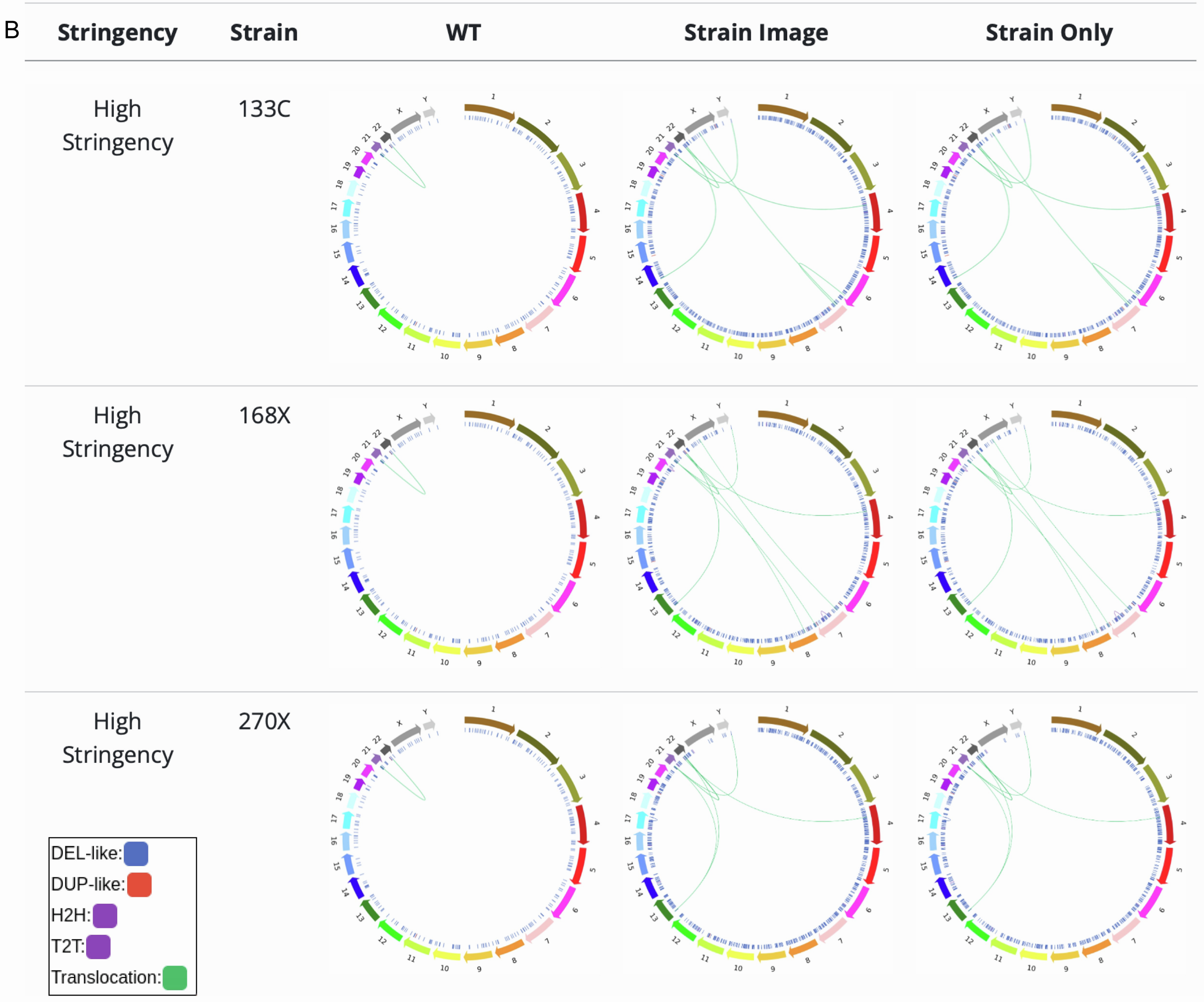
B
Stringency
Strain
WT
Strain Image
Strain Only
High Stringency
133C
High Stringency
168X
High Stringency
270X
DEL-like:
DUP-like:
H2H:
T2T:
Translocation:

Figure S5

**Figure S5.**
**A.** Grouped bar chart of structural-variant (SV) counts binned by type ($\log_{10}$ scale) for WT and mutants at high-stringency Sniffles2 settings (≥8 supporting reads, ≥150 bp).
**B.** Circos plots of SVs retained after comparison with the WT call-set ("Strain Only") for each mutant. Outer track, chromosomes 1–22, X, Y; inner chords, high-confidence SVs colored by class (legend, bottom left). Central column shows WT reference, confirming removal of shared variants.

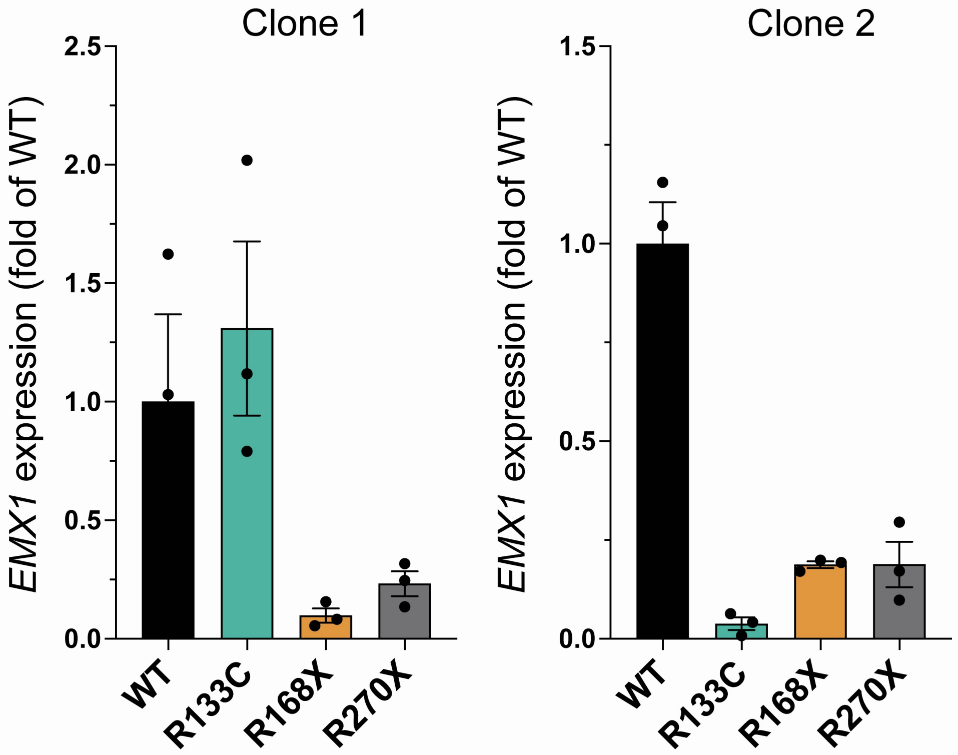
Clone 1
Clone 2
EMX1 expression (fold of WT)
2.5
2.0
1.5
1.0
0.5
0.0
WT
R133C
R168X
R270X

Figure S6

**Figure S6.**

Quantitative RT–PCR validation of *EMX1* expression in two independent CRISPR-edited hESC clone series (Clone 1 and Clone 2) expressing *MECP2-WT, MECP2-R133C, MECP2-R168X* or *MECP2-R270X* at the ESC stage. Expression is shown as fold change relative to the corresponding WT line (set to 1). Bars represent mean ± s.e.m.

A

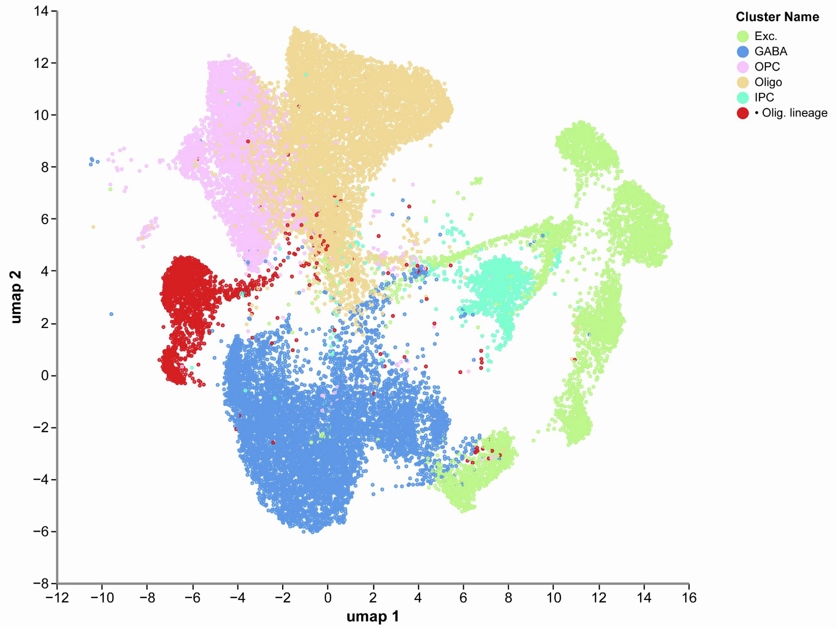
Cluster Name
Exc.
GABA
OPC
Oligo
IPC
• Olig. lineage
umap 1
umap 2

B

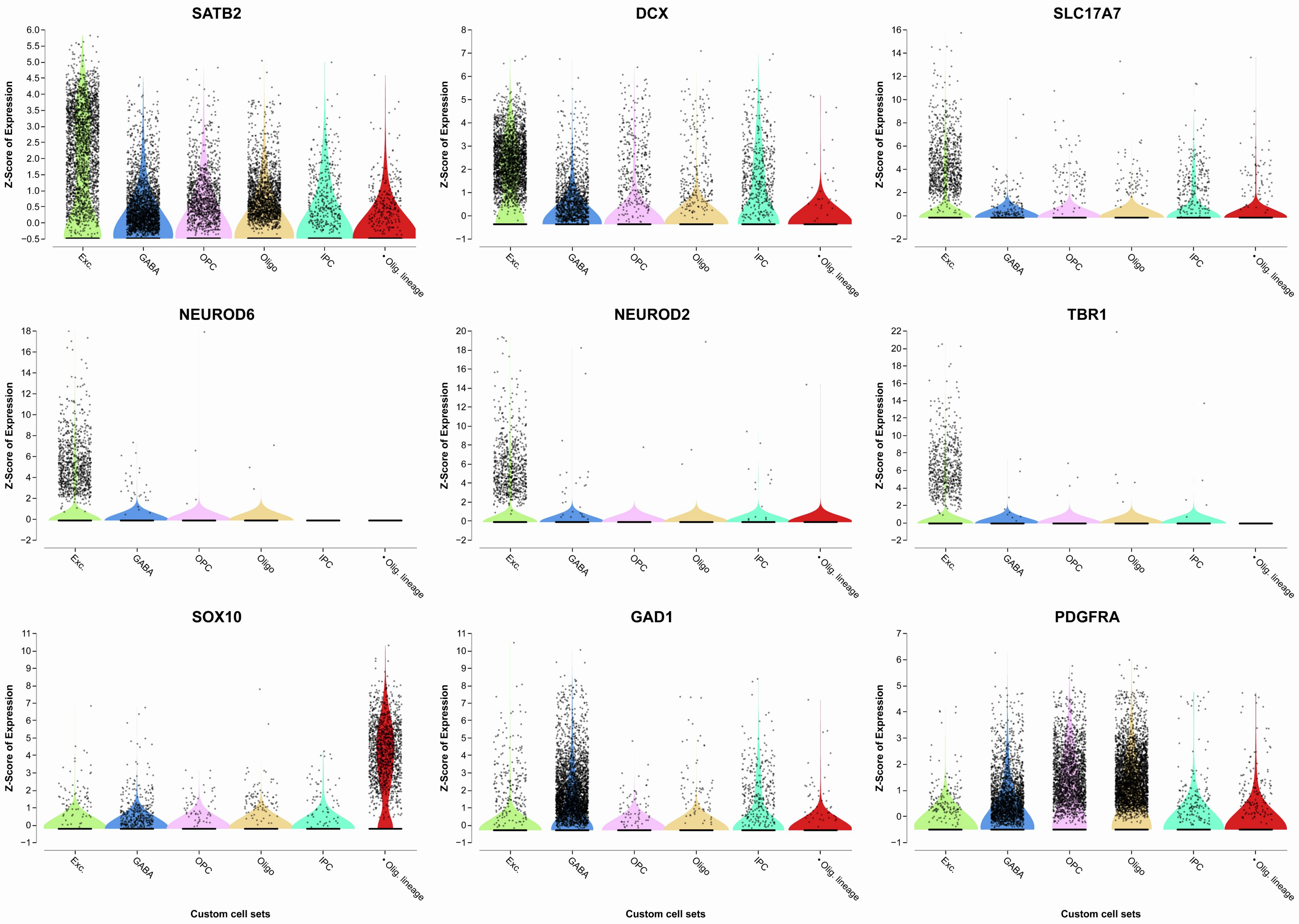
SATB2
DCX
SLC17A7
NEUROD6
NEUROD2
TBR1
SOX10
GAD1
PDGFRA
Z-Score of Expression
Exc.
GABA
OPC
Oligo
IPC
• Olig. lineage
Custom cell sets

Figure S7

**Figure S7.**

**A.** UMAP visualization of single-nucleus RNA-seq profiles from 3-month unguided cerebral organoids (all genotypes combined). Cells are colored according to the final cell-type categories used in Figure 4 (glutamatergic/excitatory neuron lineage, inhibitory/GABAergic neurons, oligodendrocyte progenitor cells, oligodendrocyte lineage and broader glial/precursor populations).

**B.** Violin plots showing expression of canonical marker genes used to assign these identities: *SATB2*, *DCX* and *SLC17A7* for glutamatergic neuron lineage, *NEUROD6*, *NEUROD2* and *TBR1* for cortical excitatory projection neurons, *SOX10* and *PDGFRA* for oligodendrocyte/OPC lineages, and *GAD1* for inhibitory/GABAergic neurons. Each dot represents a single nucleus.
